# A Target Capture-Based Method to Estimate Ploidy From Herbarium Specimens

**DOI:** 10.3389/fpls.2019.00937

**Published:** 2019-07-24

**Authors:** Juan Viruel, María Conejero, Oriane Hidalgo, Lisa Pokorny, Robyn F. Powell, Félix Forest, Michael B. Kantar, Marybel Soto Gomez, Sean W. Graham, Barbara Gravendeel, Paul Wilkin, Ilia J. Leitch

**Affiliations:** ^1^Royal Botanic Gardens, Kew, Richmond, United Kingdom; ^2^Laboratori de Botànica, Facultat de Farmàcia i Ciències de l’Alimentació, Universitat de Barcelona, Barcelona, Spain; ^3^Department of Tropical Plant and Soil Sciences, University of Hawai’i at Mânoa, Honolulu, HI, United States; ^4^Department of Botany, University of British Columbia, Vancouver, BC, Canada; ^5^UBC Botanical Garden & Centre for Plant Research, University of British Columbia, Vancouver, BC, Canada; ^6^Naturalis Biodiversity Center, Endless Forms, Leiden, Netherlands; ^7^Institute of Biology Leiden, Leiden University, Leiden, Netherlands; ^8^Science and Technology Faculty, University of Applied Sciences Leiden, Leiden, Netherlands

**Keywords:** crop wild relatives, *Dioscorea*, flow cytometry, phylogenomics, polyploidy, sequence capture, whole genome duplication, yams

## Abstract

Whole genome duplication (WGD) events are common in many plant lineages, but the ploidy status and possible occurrence of intraspecific ploidy variation are unknown for most species. Standard methods for ploidy determination are chromosome counting and flow cytometry approaches. While flow cytometry approaches typically use fresh tissue, an increasing number of studies have shown that recently dried specimens can be used to yield ploidy data. Recent studies have started to explore whether high-throughput sequencing (HTS) data can be used to assess ploidy levels by analyzing allelic frequencies from single copy nuclear genes. Here, we compare different approaches using a range of yam (*Dioscorea*) tissues of varying ages, drying methods and quality, including herbarium tissue. Our aims were to: (1) explore the limits of flow cytometry in estimating ploidy level from dried samples, including herbarium vouchers collected between 1831 and 2011, and (2) optimize a HTS-based method to estimate ploidy by considering allelic frequencies from nuclear genes obtained using a target-capture method. We show that, although flow cytometry can be used to estimate ploidy levels from herbarium specimens collected up to fifteen years ago, success rate is low (5.9%). We validated our HTS-based estimates of ploidy using 260 genes by benchmarking with dried samples of species of known ploidy (*Dioscorea alata*, *D. communis*, and *D. sylvatica*). Subsequently, we successfully applied the method to the 85 herbarium samples analyzed with flow cytometry, and successfully provided results for 91.7% of them, comprising species across the phylogenetic tree of *Dioscorea*. We also explored the limits of using this HTS-based approach for identifying high ploidy levels in herbarium material and the effects of heterozygosity and sequence coverage. Overall, we demonstrated that ploidy diversity within and between species may be ascertained from historical collections, allowing the determination of polyploidization events from samples collected up to two centuries ago. This approach has the potential to provide insights into the drivers and dynamics of ploidy level changes during plant evolution and crop domestication.

## Introduction

Whole genome duplication (WGD), or polyploidization, creates opportunities for gene functional diversification and novel genetic architectures, which may provide a basis for adaptive traits and speciation (Mayrose et al., 2011; Panchy et al., 2016; Van de Peer et al., 2017; Wendel et al., 2018). Polyploidy is common in many plant lineages. It is now recognized that all angiosperm lineages have not only undergone at least one polyploidization event during their evolutionary history, but that polyploidization is frequent and ongoing in many extant species (Otto and Whitton, 2000; Jiao et al., 2011; Wendel et al., 2016). Recent and ancient polyploidization events are also pervasive across ferns (Barker, 2013; Vanneste et al., 2015; Schneider et al., 2017; Li et al., 2018).

Ongoing polyploidization episodes continue to shape the evolutionary trajectory of many plant lineages, arising through processes ranging from genome duplication within a species (autopolyploidy) to the fusion of genome copies from independent taxa (hybridization) followed by genome multiplication (allopolyploidy; Kihara and Ono, 1926). Changes in ploidy level may play a pivotal role in various evolutionary trends in plants, with effects measured, for example, through alterations in the diversification rates in some angiosperm lineages following a WGD event (Landis et al., 2018). Polyploidy has also been linked to increased levels of speciation (Zhan et al., 2016) in angiosperms (e.g., asterids, rosids, monocots; Wood et al., 2009) and in ferns (e.g., *Deparia*, Athyriaceae; Kuo et al., 2016). However, the opposite trends have also been observed in both angiosperms and ferns, where diploids may exhibit faster diversification rates while neopolyploids suffer higher extinction rates (Mayrose et al., 2011; Schneider et al., 2017). In addition, polyploidization plays a key role in plant domestication because it can bestow favorable agricultural traits and contribute to adaptive plasticity (Salman-Minkov et al., 2016). A high proportion (84%) of the most important cultivated species are polyploid, including some of the most extensively cultivated crops worldwide (i.e., wheat, maize, soybean; Bennett, 2004; Renny-Byfield and Wendel, 2014).

Polyploidization is currently understood as a cyclic process, as WGD events typically are followed by diploidization (Sémon and Wolfe, 2007; Wendel, 2015; Qiao et al., 2019). Diploidization involves a diversity of processes, including chromosome rearrangements leading to reductions in chromosome numbers (dysploidy), and the non-random elimination or retention of duplicated genes and repetitive sequences (Comai, 2005; Wendel, 2015; Mandáková et al., 2017; Wendel et al., 2018). Diploidization following WGD may also involve discarding extra copies of dosage-sensitive single copy nuclear genes (De Smet et al., 2013), possibly reflecting selective pressures that favor single-copy status (Paterson et al., 2006), as has been suggested for housekeeping genes conserved throughout angiosperm (De Smet et al., 2013) and gymnosperm evolution (Lu et al., 2014; Salas-Leiva et al., 2014). Duplicated genes from WGD events can also be retained, for example by neo- or sub-functionalization (Innan and Kondrashov, 2010; Wei et al., 2018). Some attempts have been made to classify duplicated genes into large gene families (e.g., Maere et al., 2005; Wall et al., 2008); indeed, these genes can be used to estimate phylogenetic relationships among taxa (Yuan et al., 2009; Ness et al., 2011; Mandel et al., 2015; Moore et al., 2018). More directly, single copy nuclear genes can be used to resolve phylogenetic relationships (e.g., Chamala et al., 2015), avoiding uncertainty in paralog interpretation (Duarte et al., 2010). The number of single copy nuclear gene copies in the genome is expected to correlate with ploidy level because of the temporary excess of single copy nuclear gene duplicates. These copies arise following a WGD event independent of the fate of duplicated gene families resulting from either more ancient polyploidization events (Wendel, 2015; Panchy et al., 2016), or subgenome duplication events such as tandem duplications, segmental duplications or the activity of repetitive elements (McCann et al., 2018).

Detecting WGD is challenging (Otto and Whitton, 2000; Vanneste et al., 2012; Tiley et al., 2018), thus large-scale compilation of information on ploidy levels has lagged behind other types of data, despite methods being developed to identify paleopolyploidy events within specific species from molecular data using, for example, synonymous divergence (Ks) values as estimates of gene duplications or comparisons of gene trees (Li et al., 2015a; Tiley et al., 2018). For many years, karyological studies (i.e., chromosome morphometric characteristics) have been commonly used as the main method to reveal recent ploidy and hybridization events (e.g., Díaz-Lifante et al., 2009). As a WGD event results in a proportional increase in genome size (Wendel, 2015), at least initially, flow cytometry can also be used to estimate ploidy levels and to screen for the occurrence of multiple cytotypes in recent polyploids (Doležel et al., 2007). Flow cytometry estimates the relative DNA content present in cell nuclei, providing a microscopy-free approach to ploidy estimation. An advantage of flow cytometry is that ploidy can be estimated from both fresh and recent silica gel-dried plant tissue, assuming that a reference is available with a known chromosome number and genome size (Suda et al., 2006; Servick et al., 2015). This approach has also been shown to work on up to 12-year-old herbarium specimens (Šmarda, 2008), although success rates can vary considerably between species (Suda and Trávníček, 2006).

Herbarium collections constitute an enormous and highly valued source of information and material for botanical and agricultural research. The use of herbaria as a source of material for traditional Sanger DNA sequencing has led to substantial advances in our understanding of plant systematics and evolution. Nevertheless, such methods typically require substantial effort to amplify and sequence plastid genome targets (e.g., Viruel et al., 2016; Couto et al., 2018) or to amplify or clone a small number of nuclear markers from recently dried herbarium specimens (e.g., Särkinen et al., 2012; Viruel et al., 2018). In addition, genomic DNA extracted from herbarium materials is usually highly degraded, often requiring more complex extraction protocols to increase yields (Doyle and Dickson, 1987). While degraded DNA is not suitable for generating high-quality genome assemblies (Staats et al., 2013), recent advances in high-throughput sequencing (HTS) approaches offer the possibility of using degraded DNA to generate short-insert-size genomic libraries (e.g., 350 bp long) from natural history collection material (Dentinger et al., 2015; Bakker, 2017), and even archaeological samples (Pedersen et al., 2015). Coupled to these advances in sequencing technologies, novel bioinformatic pipelines have been developed to estimate ploidy and aneuploidy levels using whole genome sequencing data, as shown for yeast (Weiß et al., 2018), cancer cell lines (Favero et al., 2014), and plants (Tan et al., 2016).

The presence of multiple genomes complicates the estimation of gene dosage (e.g., Margarido and Heckerman, 2015; Kyriakidou et al., 2018). Some methods base ploidy level estimation on the observed allelic read depths in relation to the expected ratios predicted for different ploidy levels (Corrêa dos Santos et al., 2017). These single nucleotide polymorphisms (SNPs) methods have been applied to species with relatively simple genomes such as *Phytophthora infestans* (Yoshida et al., 2013) and yeast (Weiß et al., 2018), but also to the larger and more complex genomes of plants (Margarido and Heckerman, 2015; Zohren et al., 2016). However, to date this approach has not been optimized for analyzing HTS data obtained from museum collections.

The yam genus (*Dioscorea* L., Dioscoreaceae) is a relatively species-rich group comprising ∼625 species. It has diversified extensively across the globe (Viruel et al., 2016), and includes species that are major crops in numerous tropical countries (Tamiru et al., 2017). To date, phylogenetic studies based on ca. 25% of the species, have identified ten major clades with strong geographic signal for clade composition (Viruel et al., 2016; Couto et al., 2018). *Dioscorea* likely originated in the Laurasian Palaearctic between the Late Cretaceous and Early Eocene (Viruel et al., 2016), and the impact of both recent and ancestral polyploidization and dysploidy events may have shaped extant chromosome numbers. *Dioscorea* provides an ideal model system to study ploidy changes in evolutionary, population-level, and agronomic contexts due to the diversity of ploidy levels that have been uncovered from chromosome counts, which are available for ca. 15% of the species. These data show that haploid numbers (i.e., *n* = number of chromosomes per gamete) range from *n* = 7 to 72 (Viruel et al., 2008). Most of this diversity is due to polyploidy, with the majority of the species reported so far being tetraploids, although ploidy levels can reach 14*x* (Essad, 1984). Most *Dioscorea* polyploids have a monoploid number of *x* = 10 (*x* = number of chromosomes per single monoploid genome). Dysploid reductions, which can be followed by subsequent rounds of polyploidy, have also been reported in some New World (*x* = 7 and *x* = 9; Viruel et al., 2008, 2010) and Mediterranean taxa (*x* = 6 and *n* = 12, 24 or 48 bivalents at meiosis; Heslot, 1953; Al-Shehbaz and Schubert, 1989). The latter difference between the monoploid number of chromosomes per cell (*x*) and the number of chromosomes contained in gametes (*n*) was explained by an ancestral amphiploidy based on the existence of up to four alleles per microsatellite markers with a disomic inheritance (Catalán et al., 2006). While many of the chromosome counts are based on the analysis of a single specimen, multiple ploidy levels (cytotypes) have been revealed when more than one individual of a species is analyzed, as reported for several cultivated yam species (e.g., *D. alata* L., *n* = 10, 15, 20, 25, 30, 35 or 40; Viruel et al., 2008 and references therein). This is not an uncommon occurrence in plants with many species showing a ploidy series (Stebbins, 1950; Levy and Feldman, 2002; Husband et al., 2013). Genome size also varies considerably in the genus, with a 6.8-fold range in 1C-values for 23 species, from 0.35 pg/1C (Obidiegwu et al., 2009), to 2.4 pg/1C (Bharathan et al., 1994). Chromosome counts alone do not necessarily allow the prediction of ploidy levels in *Dioscorea*. While counts of 2*n* = 40 (2*n* = number of chromosomes in a somatic cell) in the two main yam crops (*D. alata* and *D. rotundata* Poir.) predicted they were tetraploid, diploid-like patterns were observed in microsatellite (Nemorin et al., 2013) and SNP data (Tamiru et al., 2017; Cormier et al., 2019), supporting the hypothesis that they are diploidized polyploids.

Knowing the ploidy level of a species and quantifying within-species cytotype diversity is essential for enhancing our understanding of the systematics and evolution of plants (Corrêa dos Santos et al., 2017). Here, we use *Dioscorea* as a model system to: (i) determine the extent to which ploidy level can be estimated from herbarium specimens using flow cytometry, and (ii) explore and optimize a bioinformatics approach using HTS Hyb-Seq data (Weitemier et al., 2014) generated from targeted baits developed from low and single copy nuclear (LSCN) genes for discovering recent polyploids that have yet to fully diploidize their genomes.

## Materials and Methods

### Plant Material Used for Estimating Ploidy Level by Flow Cytometry

Specimens used for chromosome counts and ploidy level analysis by flow cytometry are listed in Tables 1A, 1B, and Supplementary Table S1. Root tips for chromosome counts were obtained from freshly germinated seeds. Ploidy levels from three different types of plant material were analyzed using flow cytometry to increase information on ploidy diversity across the genus, and from plants dried under three different regimes to determine how different preservation methods impact our ability to predict ploidy levels. These three material types were: (i) fresh leaf material obtained from mature plants growing in the collections at the Royal Botanic Gardens, Kew (RBGK) or from germinated seedlings (Table 1A), (ii) silica gel-dried specimens, and (iii) 85 herbarium specimens from the herbaria of RBGK (K) and Leiden (L) collected between 1831 and 2011 (Table 1B and Supplementary Table S1).

**TABLE 1A:** Fresh plant material used in the present study for chromosome counts and flow cytometry analyses in *Dioscorea*.

				**Flow cytometry analyses**						
**Species name**	**Sample information**	**Chr. no. (2*n*)**	**Clade**	**CV sample**	**CV standard**	**1C (pg)**	**Predicted ploidy based on flow cytometry**	**1Cx**	**Predicted ploidy based on HTS data**	**M**
*D. alata* L.	RBGKLiv 1982-1316	20 to 80	Enantiophyllum	3.39	2.77	0.84	6*x*	0.28		
	RBGKLiv PalmH. (R89)			4.55	3.53	0.56	4*x*	0.28	≥4*x*	2.8
	RBGKLiv 1987-1993			4.51	3.48	0.59	4*x*	0.30		
*D. altissima* Lam.	RBGKLiv 2005-1233 (R92)		CL	4.50	3.76	0.68	4*x*	0.34	≥4*x*	2.4
*D. antaly* Jum. and H.Perrier	RBGKLivMSB-406347		CL	5.44	1.93	0.33	2*x*	0.33		
	RBGKLiv 1998-523			3.44	1.79	0.31	2*x*	0.31		
	RBGKLiv 2014-641			4.23	2.48	0.33	2*x*	0.33		
	RBGKLiv 1980-2270 (R85)			3.10	2.50	0.70	4*x*	0.35	≥4*x*	2.5
*D. bemarivensis* Jum. and H.Perrie	MSB 339267		Malagasy	4.67	2.81	0.84	^*^6*x*	0.28	≥4*x*	3.0
*D. brownie* Schinz	MSB 508171	**20**	Africa	5.12	1.78	0.38	2*x*	0.38	2*x*	1.2
*D. bulbifera* L.	RBGKLiv JodrellN	36–100	CL	4.48	1.83	0.36	2*x*	0.36		
	RBGKLiv 2000-2561			2.18	2.47	1.57	8*x*	0.39		
*D. rotundata* Poir.	RBGKLiv 1920-76.01470 (R88)	36–140	Enantiophyllum	3.00	2.20	0.72	4*x*	0.36	≥4*x*	2.7
*D. caucasica* Lipsky	RBGKLiv 2014-1847	20	Stenophora	5.02	2.78	0.51	2*x*	0.51	2*x*	1.4
*D. communis* (L.) Caddick and Wilkin	RBGKLiv 1969-19666 (R84)	48	Mediterranean	4.09	2.52	1.08	6*x*	0.36	6*x*	2.7
	RBGKLiv JLMN182 (R86)			4.45	2.92	0.49	4*x*	0.33	4*x*	2.5
	LivSpec (R01)	**ca. 36**		3.80	1.83	0.97	6*x*	0.32	6*x*	2.7
*D. composita* Hemsl.	RBGKLiv 1969-11715	36	NWI	2.89	1.80	0.47	^*^2*x*	0.47		
	RBGKLiv 1978-1830 (S44)			2.37	1.60	0.48	^*^2*x*	0.48	2*x*	1.5
*D. decipiens* Hook.f.	RBGKLiv 1998-4297		Enantiophyllum	3.70	1.90	0.74	^*^4*x*	0.37	≥4*x*	2.9
*D. deltoidea* Wall. ex Griseb.	RBGKLiv 1963-26702 (R83)	20, 40	Stenophora	4.30	2.70	0.65	^*^4*x*	0.33	≥4*x*	2.3
*D. dumetorum* (Kunth) Pax	RBGKLiv 1984-8045b	36 to 54	CL	6.80	3.40	0.41	^*^2*x*	0.41		
	RBGKLiv 1984-8045 (S48)			6.00	3.50	0.41	^*^2*x*	0.41	2*x*	1.7
*D. elephantipes* (L’Hér.) Engl.	RBGKLiv POW		Africa	10.00	2.70	0.48	2*x*	0.48	2*x*	1.3
				9.20	2.72	0.46	2*x*	0.46		
*D. galeottiana* Kunth	MSB 780962	**40**	NW				4*x*		≥4*x*	2.9
*D. glabra* Roxb.	RBGKLiv 1996-4312 (S43)	40	Enantiophyllum	3.80	3.5	0.69	^*^4*x*	0.35	2*x*	1.4
*D. membranacea* Pierre ex Prain and Burkill	RBGKLiv 1998-4292		Stenophora	5.50	3.60	0.81	^*^4*x*	0.41		
	RBGKLiv 1998-4294			4.80	3.30	0.86	^*^4*x*	0.43		
*D. minutiflora* Engl.	RBGKLiv 1960-1001 (S45)	>120	Enantiophyllum	3.90	1.90	0.64	^*^4	0.32	2*x*	1.5
*D. pentaphylla* L.	RBGKLiv Jod	40 to 144	CL	3.00	2.90	1.14	^*^8*x*	0.29		
	RBGKLiv 1996-4313 TN (S52)			4.20	3.00	1.15	^*^8*x*	0.29	≥4*x*	2.9
	RBGKLiv 1996-4313bis TB			3.70	2.60	1.19	^*^8*x*	0.30		
	RBGKLiv 1996-4313 Jod			4.60	3.40	1.29	^*^8*x*	0.32		
*D. pteropoda* Boivin ex H.Perrier	MSB 459767	**20**	Malagasy				2*x*		2*x*	1.8
*D. polystachya* Turcz.	RBGKLiv Jod (R71)	140	Enantiophyllum	4.30	3.70	1.75	10*x*	0.35	≥4*x*	3.0
*D. praehensilis* Benth.	RBGKLiv 1960-1002 (R87)	40, 80	Enantiophyllum	3.30	2.80	0.62	4*x*	0.31	≥4*x*	2.7
	MSB 171063			4.55	3.08	0.78	5*x*	0.31		
*D. preussii* Pax	RBGKLiv 1968-57006 (R90)	40, 54	Birmanica	3.70	3.60	1.14	^*^8*x*	0.29	≥4*x*	2.5
*D. rockii* Prain and Burkill	RBGKLiv 1996-4307 (R82)		Stenophora	4.60	2.30	0.92	^*^6*x*	0.31	≥4*x*	2.2
*D. sansibarensis* Pax	RBGKLiv 1969-5387	40	Malagasy	6.90	3.50	0.36	2*x*	0.36	2*x*	1.3
*D. saxatilis* Poepp.	MSB 350565		NWI	3.94	1.94	1.04	^*^6*x*	0.35	≥4*x*	3.0
*D. soso* Jum. and H.Perrier complex	RBGKLiv 2014-1312		Malagasy	4.60	2.20	1.04	^*^6*x*	0.35		
	RBGKLiv 2008-3097A			9.70	4.50	1.28	^*^8*x*	0.32		
	RBGKLiv 2008-3097B			3.90	3.30	1.23	^*^8*x*	0.31		
	RBGKLiv 2008-3097C (R91)			4.10	1.90	1.24	^*^8*x*	0.31	≥4*x*	2.3
	RBGKLiv 2008-3097D (S47)			3.20	2.50	1.22	^*^8*x*	0.31	≥4*x*	2.9
	RBGKLiv 2005-1802b (S50)			5.20	4.60	0.61	^*^4*x*	0.31	2*x*	1.4
	RBGKLiv 2005-1802			4.70	3.20	0.69	^*^4*x*	0.35		
*D. strydomiana* Wilkin	MSB 565198		Africa	5.80	5.80	0.43	2*x*	0.43	2*x*	1.8
*D. sylvatica* Eckl.	RBGKLiv 1963-26705		Africa	9.40	4.10	0.49	2*x*	0.49		
	RBGKLiv 2011-447 (S49)			7.10	4.44	0.51	2*x*	0.51	2*x*	1.3
	MSB 564412			5.64	3.31	0.41	2*x*	0.41		

RBGKLiv, Living collection at Royal Botanic Gardens, Kew. MSB, Millennium Seed Bank. Chromosome counts done in this study are highlighted in bold, the remaining were obtained from Viruel et al. (2008) and references therein. Clade names follow Viruel et al. (2016): CL = compound leaf, N1 and II = New World 1 and II. The predicted ploidy is based on approximate 1C-value estimated for the species; or ^*^ compared with the closest relative where a robust chromosome number and genome size have been estimated. For HTS data, ploidy level was estimated through the HTS-based method described in this study; and *M*, the median value of the allelic ratio.

**TABLE 1B:** Estimation of ploidy level in six *Dioscorea* species from five herbarium samples from RBG Kew (K) and one silica dried sample (S) using flow cytometry.

			**Cytometry analyses**							
**Species name**	**Clade**	**Sample information**	**Type of material**	**CV sample**	**CV standard**	**Approx. 1C (pg)**	**Predicted ploidy based on flow cytometry**	**1Cx**	**Predicted ploidy based on HTS data**	**M**
*D. communis* (L.) Caddick and Wilkin	Mediterranean	Viruel S80 – 2015	S	10.83	3.47	0.41	3*x*	0.27	3*x*	2.0
		Chase et al. 7100 – S42	S	12.56	3.60	0.54	4*x*	0.27	4*x*	2.6
		Médail R28 – 2016	S	6.77	1.90	0.59	4*x*	0.30	4*x*	2.2
		Viruel 2016 – Pop Maroc1	S	7.09	1.92	1.29	8*x*	0.32		
		Christenhurz et al. 7100 (2017) R27	S	8.40	1.20	1.29	8*x*	0.32	8*x*	2.8
		Viruel R29 – 2016	S	12.07	2.55	1.37	8*x*	0.34	8*x*	2.8
		Médail R30 – 2016	S	11.21	2.21	1.36	8*x*	0.34	8*x*	2.8
*D. modesta* Phil.	NWI	K001150677 (Casado INIA-LP 015. Chile. 2002) (W73)	K	22.01	2.78	0.50	^*^2*x*	0.50	2*x*	1.5
		“	K	18.66	3.11	0.50	^*^2*x*	0.50		
*D. soso* Jum. and H.Perrier complex	Malagasy	K000062164 (Ranarivelo et al. RLI952. Madagascar) 2008 (W63)	K	22.01	2.63	0.66	^*^4*x*	0.33	2*x*	1.3
		“	K	20.25	2.46	0.64	^*^4*x*	0.32		
*D. multiflora* Mart. ex Griseb.	NWI	K001171677 (Iganci J.R.V. et al. 813-Brazil) 2011 (W59)	K	16.50	3.22	0.45	^*^2*x*	0.45	2*x*	1.3
*D.* “ovy-valiha”	Malagasy	K000523550 (Ranirison 714. Madagascar) 2004 (W81)	K	2.83	3.28	2.05	^*^12*x*	0.34	≥4*x*	3.0
			K	3.60	3.00	2.06	^*^12*x*	0.34		
*D. trilinguis* Griseb.	NWI	K000579751 (Zappi et al. 953. Brazil) 2008 (Z74)	K	19.18	3.46	0.70	^*^4*x*	0.35	≥4*x*	2.3
			K	14.79	2.83	0.67	^*^4*x*	0.34		

*For clade names and information on data in columns see Table 1A.*

The three drying methods were implemented by first analyzing ploidy level from fresh leaves of 36 specimens belonging to 20 species, and then drying additional fresh leaves from the same individuals (i) in silica gel, (ii) pressed between herbarium sheets and kept at room temperature in a plant press using newspaper and absorbent blotting paper, and (iii) pressed as (ii) between herbarium sheets, dried in an oven at 60°C for 48 h and then kept at room temperature (Supplementary Table S2). Six of the dried samples from (i) to (iii) were analyzed using flow cytometry after three months, and all of them after 20 months.

### Chromosome Counts

Seeds were germinated on agar plates, and newly emerging roots were treated following Viruel et al. (2008). Root tips were soaked in 2 mM 8-hydroxyquinoline for 4 h at room temperature (Tjio and Levan, 1950), fixed in Carnoy I (3:1 (v/v) ethanol:acetic acid), and then stored at 4°C until staining. Chromosome counts were made from metaphase plates which were prepared by staining roots in propidium iodide (PI, Sigma) (1 mg/mL) and 4′,6-diamidino-2-phenylindol (DAPI, 1 mg/ml) in a combined PI-DAPI (CPD) staining solution (Andras et al., 2000), and then squashed in 45% acetic acid and observed using a Leica DM6000 microscope. Leica Application Suite X (LAS X) software was used to capture images and to count chromosomes.

### Ploidy Estimation by Flow Cytometry

Ploidy levels were estimated using a CyFlowSL Partec flow cytometer (Partec GmbH, Göttingen, Germany) following the one-step protocol of Doležel et al. (2007) with minor modifications as described in Clark et al. (2016). We selected parsley, *Petroselinum crispum* (Mill.) Fuss “Champion Moss Curled,” which is a diploid with 2*n* = 2*x* = 22 and has a genome size of 2C = 4.50 pg (Obermayer et al., 2002), as the internal calibration standard for ploidy screening in *Dioscorea*. We used the “CyStain PI Absolute P kit” (Sysmex, United Kingdom) nuclei isolation buffer, following the manufacturer’s instructions. An improved “Cystain PI OxProtect” (Sysmex, United Kingdom) buffer was released during this study and we used this version for samples dried 20 months ago (see below). For some herbarium samples it was necessary to analyze up to three times as much plant material (i.e., approximately 2 cm^2^ of tissue) compared to analyses using fresh leaf material, in order to obtain enough nuclei for analysis. The ploidy level of each species that generated peaks of sufficient quality in the resulting flow histograms was estimated by comparing the 1C-value estimate obtained in this study with either that of the same species with a published genome size and known ploidy level, or, if not available, with the genome size and ploidy level of the closest diploid relative (Tables 1A, 1B; Pellicer and Leitch, 2014; Viruel et al., 2016). We also compiled published information on chromosome counts and genome size estimates from flow cytometry for the studied *Dioscorea* species (Viruel et al., 2008; Bennett and Leitch, 2014; Rice et al., 2015), summarized in Tables 1A, 1B.

### Plant Material Used for Estimating Ploidy Level Bioinformatically From HTS Hyb-Seq Data

DNA was extracted from 95 samples, including herbarium specimens, representing three species (Supplementary Table S3) to implement the HTS Hyb-Seq method described below for the analysis of ploidy level in herbarium samples. These comprised: (i) *Dioscorea sylvatica* Eckl., an African wild species that is predicted to be diploid based on preliminary flow cytometry analyses (27 samples collected between 1917 and 2013), (ii) *D. communis* (L.) Caddick and Wilkin, a Mediterranean wild species reported to be polyploid with 2*n* = 4*x* = 48 (58 samples collected between 1893 and 2018), and (iii) *D. alata*, a crop species reported to have all possible chromosome counts in multiples of the monoploid number *x* = 10 from 2*n* = 20 up to 80 (10 samples collected between 1836 and 2017). Specimens were obtained from Kew, Leiden, and Sevilla herbaria. Full details of the origin and age of each analyzed specimen are shown in Supplementary Table S3.

### Bioinformatic Ploidy Estimation From HTS Hyb-Seq Data

We validated a pipeline combining Hyb-Seq data (Weitemier et al., 2014) and existing bioinformatic tools (Corrêa dos Santos et al., 2017; Weiß et al., 2018) to estimate ploidy level using data obtained from the three species noted above (*D. sylvatica*, *D. communis*, and *D. alata*). Total DNA was extracted using a modified CTAB protocol for herbarium specimens (Doyle and Doyle, 1987). A Dioscoreaceae RNA-baits capture kit was designed to enrich 260 LSCN genes (De Smet et al., 2013; Soto Gomez et al., 2019). Genomic libraries were prepared using NEBNext^®^ Ultra^TM^ II DNA Library Prep Kit for Illumina^®^ (New England Biolabs, Ipswich, MA, United States) with AMPure XP magnetic beads and NEBNext^®^ Multiplex Oligos for Illumina^®^ (Dual Index Primer Sets I and II) as barcodes for simultaneous sequencing. Enriched libraries were prepared following the myBaits^®^ kit manual v3.02 (Arbor Biosciences). Library quality was evaluated using a Quantus^TM^ fluorometer (Promega Corp.) and an Agilent 4200 TapeStation (Agilent Technologies, Santa Clara, CA, United States). Multiplexed libraries were then sequenced on a HiSeq X platform (Illumina, Inc.) lane.

Trimmomatic v0.35 (Bolger et al., 2014) was used to remove low-quality reads and adapter sequences flagged by FastQC v0.11.7 (Andrews, 2010). Burrows-Wheeler Aligner (BWA; Li and Durbin, 2009) was used to map reads against the reference genes from three *Dioscorea* transcriptomes of the studied species (*D. alata*, *D. communis*, and *D. sylvatica*). Mapped reads were visualized using ploidyNGS software (Corrêa dos Santos et al., 2017) to perform a preliminary exploration of allele frequencies in each sample.

The nQUIRE software (Weiß et al., 2018) was used to estimate the ploidy model that best fits the distribution of SNPs found among mapped reads for each sample. This method estimates the allelic frequency of biallelic SNP states and assumes that different allelic ratios will be generated for diploids (0.5/0.5), triploids (0.33/0.66), and tetraploids (0.25/0.75 and 0.5/0.5). It also discards noise by removing frequencies below 0.2, as recommended by Corrêa dos Santos et al. (2017). We followed a three-factor validation to estimate the ploidy level of each sample: (i) direct observation of the histograms showing the distribution of allele frequencies, (ii) best fit between ideal and empirical histograms (i.e., low sum of squared residuals (SSR), positive slope (*y*–*y*) values, low standard error, and high *R*^2^), and (iii) the lowest delta likelihood values after comparing experimental data against the maximized log-likelihood of the free model. The percentage of polymorphic sites for each sample was calculated as the percentage of SNP positions and the total number of base pairs.

Additional allelic frequency peaks would be expected for higher ploidy levels as simulated by Corrêa dos Santos et al. (2017). To explore the existence of higher ploidy levels, the ratio per SNP of the allelic coverage was calculated and plotted, assuming, for example, that a diploid would have a range centered around a ratio of one (1:1), and a tetraploid would have two modal values at one (2:2) and at three (3:1) (see Supplementary Table S5 and Supplementary Data Sheet S1). This estimation aims to balance the height of the peaks recovered for different ploidy levels by decreasing the double peaks that 0.5 allelic frequencies generate, and to reduce the number of peaks in the graphs. Higher ploidy levels would contain proportions that would be over-shadowed by the sequencing noise, i.e., those allelic frequencies below 0.2. For example, a hexaploid specimen would contain SNPs with the ratios of 5:1, 4:2, and 3:3; however, the 5:1 ratio requires allelic frequencies of 0.2 that cannot be distinguished from allelic frequencies of SNPs created by sequencing errors. The number of possible ratios, and the number of the shadowed ratios, increase at higher ploidy levels (see Supplementary Table S5 and Supplementary Data Sheet S1), complicating the assignment of a sample to a ploidy level greater than 4*x*.

From the data analyzed in this study, density plots, histograms, and boxplots were estimated for each sample and then compared to the expected density plots for each ploidy level (Supplementary Table S5 and Supplementary Data Sheet S1), which were determined using the standard deviations calculated from 1,000 bootstrap replicates from known diploid density curves using a custom script in R (R Core Team., 2018) using the package *bootstrap*. Allelic ratios were also estimated for each gene and SNP per sample.

## Results

### Ploidy Estimation Using Chromosome Counts and Flow Cytometry

Chromosome counts were obtained for four species (Table 1A), two diploid (2*n* = 2*x* = 20; *D. brownii* Schinz and *D. pteropoda* Boivin ex H.Perrier), one tetraploid (2*n* = 4*x* = 40; *D. galeottiana* Kunth) and one hexaploid (2*n* = 6*x* = ca. 36; *D. communis*).

Ploidy estimates using flow cytometry were obtained for all fresh material samples, with a mean coefficient of variation (CV) of 4.7 ± 1.7% (Table 1A). For *D. bulbifera* L., *D. communis*, and *D. soso* Jum. and H.Perrier, analysis of several individuals revealed intraspecific variation in ploidy level. Based on previously published genome size data for comparison, the flow cytometry data obtained here suggest that the analyzed species comprise a range of ploidy levels from diploids in several species to the predicted dodecaploid in *D.* “ovy-valiha” (Table 1B).

Only five of the 85 herbarium samples (5.9%) processed using flow cytometry (Supplementary Table S1) were of high enough quality to estimate ploidy level (Table 1B). The oldest sample that produced analyzable results was *D. modesta* Phil., collected in 2002 (Table 1B), with the data suggesting it is diploid. Seven silica gel-dried samples of *D. communis* collected in 2015 also yielded data that indicated they comprised one triploid, two tetraploid and four octoploid individuals (Table 1B).

Seven samples representing six species were analyzed using flow cytometry three months after each was dried under three different regimes (Supplementary Table S2, see section “Materials and Methods”). We obtained data from four silica-dried samples, five oven-dried samples, and five dried at room temperature (Supplementary Table S2). Regardless of the increasing CV values, ploidy level estimates for each dried sample for which flow cytometry was successful were the same as those estimated from fresh material of the same individual after three months (Supplementary Table S2). Most of the silica-dried samples (91.2%) produced analyzable results 20 months after having been dried, however, only 29% of oven-dried and 41% of room-temperature dried material produced results. An observed increase in the average estimated 1C-values differed among treatments (silica-dried samples 29.4%, oven-dried 16.7% and room-temperature dried 7.9%), even though the increase in CV values was similar among treatments (CV dried/CV fresh: silica-dried 3.3, oven-dried 2.9 and room-temperature dried 3.4, on average).

### Bioinformatic Ploidy Estimation From HTS Hyb-Seq Data

On average, 353,250 ± 71,195 bp of sequence data were obtained for each *Dioscorea* sample, which resulted in an average retrieval of 86.8% of the total length of the 260 reference genes. No differences in the length of sequences were found between the three species, *D. sylvatica*, *D. alata*, and *D. communis*, whose ploidy levels were analyzed using the nQuire pipeline. The total number of SNPs per sample ranged from 641 to 12,721, with an average coverage of 147*x* (range 25*x*–1,030*x*) across the 260 target genes. A wider range in the number of SNPs was found in *D. communis* samples (547–12,721) compared with *D. alata* (1,056–5,298) and *D. sylvatica* (1,413–3,441). By contrast, *D. sylvatica* samples had the highest coverage values (72*x*–1030*x*) compared with *D. communis* (25*x*–547*x*) and *D. alata* (55*x*–166*x*).

**FIGURE 1:**
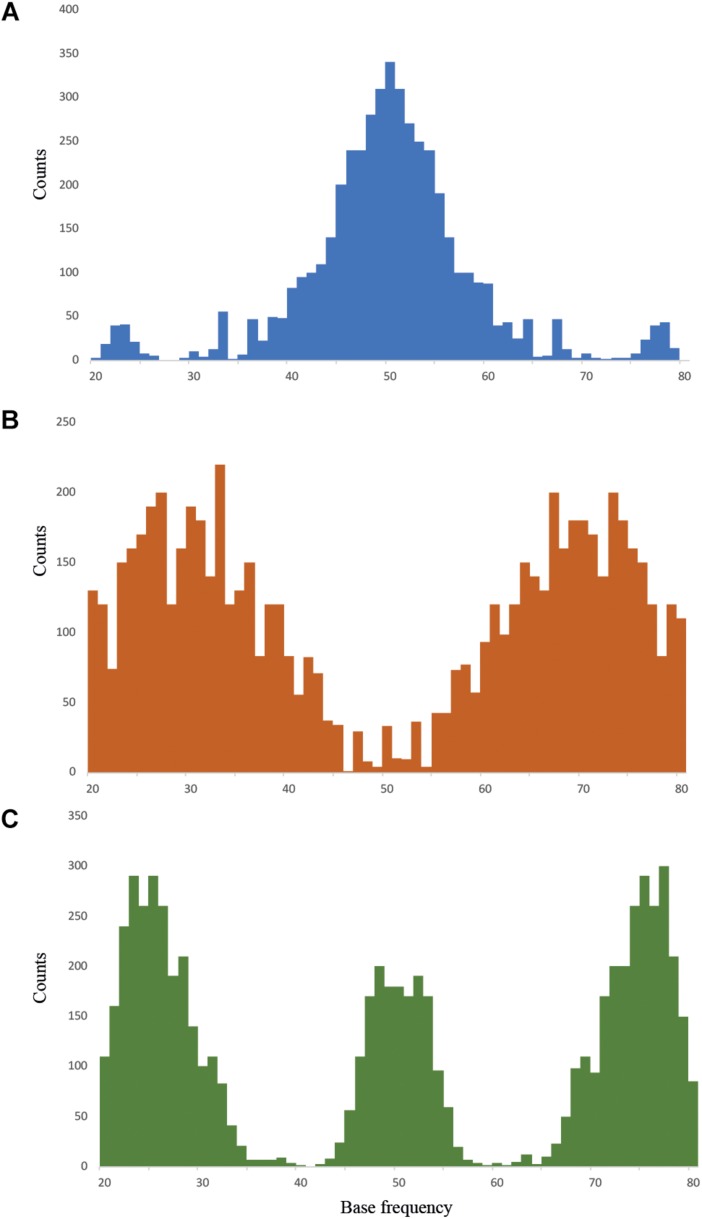
Allelic frequency patterns found in **(A)** diploid, in blue (*Dioscorea sylvatica* R104), **(B)** triploid, in orange (*D. alata* T38), and **(C)** tetraploid, in green (*D. communis* P06), models using nQuire.

The *Dioscorea* samples analyzed here included individuals that fit one of the three ploidy models (di-, tri-, and tetraploids) for which the nQuire pipeline specifically tests. The diploid pattern fit with the allelic frequencies around a value of 50%, the triploid pattern showed two peaks at 33% and 67%, and the tetraploid model had three peaks: the diploid 50% allelic frequency, together with an extra 25% and 75% (Figure 1). Of the 95 samples analyzed, nQuire was able to fit one of the three ploidy models to 92 samples (i.e., 96.8%; Supplementary Table S3). The three samples that could not be assigned to a ploidy model were from *D. communis*. The samples estimated as diploid, triploid or tetraploid showed different patterns for all the statistics calculated for the three models: i.e., lower Δlog*L* and a good fit between ideal and empirical histograms or low SSR scores (highlighted in bold in Supplementary Table S3). Aside from a visual inspection of the distribution of allelic frequencies in the histograms, the parameters that best defined diploid patterns were, in no particular order, Δlog*L*, SSR, *y*–*y* slope, SE, and *R*^2^ (Supplementary Table S3).

One of the 27 *D. sylvatica* samples showed a tetraploid pattern (R103), while the 26 remaining samples all produced histograms with diploid allelic frequency distributions (Supplementary Table S3). Eight of the 58 *D. communis* samples were estimated to be diploid (Supplementary Table S3); this was supported by a positive *R*^2^ value similar to the patterns found in diploid *D. sylvatica* samples. Most *D. communis* samples (47 of 58) were predicted to be tetraploids, and the remaining three samples remained inconclusive. However, two of the individuals that were inconclusive had Δlog*L* values that fit both triploid and tetraploid patterns and rejected the diploid model (R95 and S71). Therefore, 84.5% of *D. communis* samples were defined as polyploids by nQuire. In addition to a visual inspection of the histograms showing the distribution of the allelic frequencies, the parameters that best-defined tetraploid patterns for this species were, in no particular order, Δlog*L*, Norm SSR and *y*–*y* slope (Supplementary Table S3). *Dioscorea alata* recovered patterns consistent with four diploids, five tetraploids and one triploid (Supplementary Table S3). The diploid and tetraploid samples produced similar histograms and statistics to those observed in diploid and tetraploid samples of *D. sylvatica* and *D. communis*. The sample predicted to be triploid (T38) was based on visual interpretation of the histogram showing allelic frequency patterns, the lowest Δlog*L* score out of the three ploidy models, and the most positive *y*–*y* slope.

### Factors Influencing Ability to Predict Ploidy Level in nQuire

*(a) Impact of heterozygosity*Heterozygosity could be different in polyploids compared to diploids due to the existence of additional monoploid genomes in the nucleus (i.e., considering allele variation across all homeologous instances of a locus). To explore this the percentage of polymorphic sites was determined for each sample by calculating the relative proportion of SNP counts against the total number of base pairs, to test whether this measure of heterozygosity contributed to distinguishing polyploids from diploids (Supplementary Table S3). The average percentage of polymorphic sites in *D. sylvatica* (0.54% ± 0.13) differed significantly (U de Mann–Whitney, p < 0.005) from that of *D. communis* (0.72% ± 0.77) and *D. alata* (1.37% ± 0.75); the latter two species had more predicted polyploids than diploids. However, this method shows a high dependency on the number of SNPs, as the three samples for which the ploidy level could not be accurately assigned also showed the lowest heterozygosity values. For *D. communis* there was a general tendency of higher percentages of polymorphic sites for tetraploids when results were split into ploidy levels (diploid: 0.61% ± 0.66; tetraploid: 0.79% ± 0.81), although several predicted tetraploids did have lower percentages of polymorphic sites compared to some diploids (Supplementary Table S3). For *D. alata*, the four diploid samples had the highest percentage of polymorphic sites (1.55% ± 0.66) compared to the triploid specimen (0.83%) and four tetraploid samples (1.33% ± 0.90). Given these inconsistent trends, it seems clear that the percentage of polymorphic sites is not linked to the ploidy level status of a sample and should not be considered a reliable parameter to estimate ploidy level.

*(b) Impact of sequence coverageDioscorea sylvatica* and *D. communis* samples had higher average coverage values (88–1348*x* and 35–1460*x*, respectively) than *D. alata* (66–287*x*). Samples for which nQuire could not define the ploidy level had coverage values similar to those for which ploidy level was resolved, although the relative heterozygosity was lower in most of the undefined samples (Supplementary Table S3). Overall, the ability to distinguish ploidy levels was not dependent on the level of sequence coverage in our data.

### Distribution of Allelic Ratios

**FIGURE 2:**
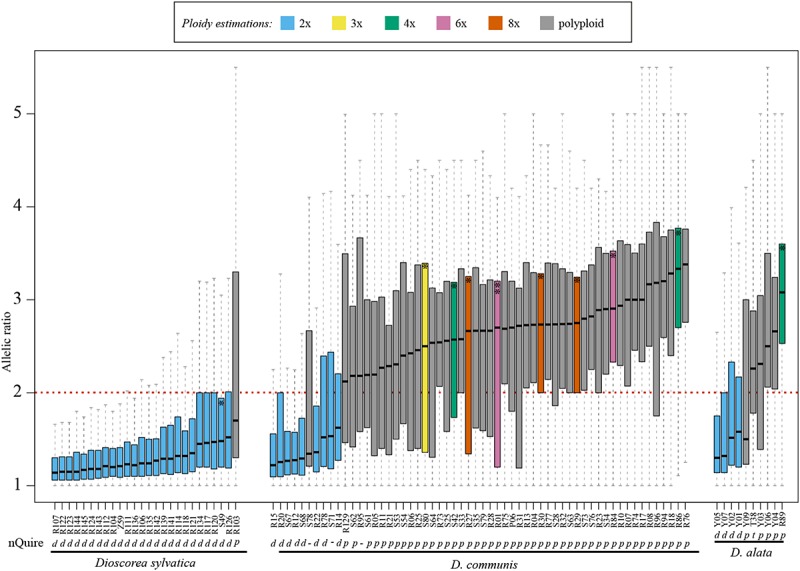
Boxplots of the allelic ratios calculated using Hyb-Seq data based on 260 low and single copy nuclear (LSCN) genes for 27 samples of *Dioscorea sylvatica*, 58 samples of *D. communis* and 10 samples for *D. alata*. Blue and gray colors represent the predicted ploidy level based on the median values of the allelic ratios: diploid and polyploid, respectively. The sample IDs correspond to those detailed in Supplementary Table S3 ordered by increasing median. One asterisk in a boxplot indicates samples with an estimated 1C-value (see Table 1A), while the *D. communis* sample with two asterisks (R01) indicates that both a 1C-value and chromosome count were performed (Table 1A). The ploidy estimations obtained from nQuire are shown below the sample IDs: d, diploid; t, triploid; p, polyploid; -, unclear (i.e., the third model determined by nQuire is tetraploid, however, our results suggest that other ploidy levels fit with this model).

The distribution of allele ratios (i.e., number of reads of the most frequent allele divided by number of reads of least frequent) for each sample was clearly different between diploids and polyploids in all three species (Figure 2), and even within predicted polyploids, distinctive distributions were observed (Figure 2). We calculated all expected allelic ratios of read frequencies among SNP biallelic states for each ploidy level up to 16*x* in order to further explore and understand how and why these allele ratios varied across ploidy levels (Supplementary Table S5 and Supplementary Data Sheet S1). Note that some of these ratios would not be detected in our analyses because they would fall within the allelic frequencies that are artificially created by sequencing errors and are therefore removed by the nQuire pipeline as sequencing noise (see section “Materials and Methods” and Supplementary Table S5 and Supplementary Data Sheet S1). These predicted allelic ratio patterns were compared to those observed in both the histograms (Supplementary Table S3) and density plots for each sample (Supplementary Table S4). In all cases, diploid samples of *D. sylvatica* showed a single and clear peak around the allele ratio value of one (Supplementary Table S4).

The median value for the allelic ratios of each sample varied between and within species (Figure 2). All samples predicted to be diploid had median allelic ratio values <2 (1.13–1.52 in *D. sylvatica*; 1.22–1.62 in *D. communis*, and 1.30–1.58 in *D. alata*; Figure 2 and Supplementary Table S4). All samples identified as tetraploids by nQuire had double the median values (>2, range 2.00–3.00) of predicted diploid samples. However, in *D. sylvatica* the median value of allelic ratios for the single predicted tetraploid specimen was 1.72, which could be explained by it being a recent autopolyploid (see below). In *D. communis* it ranged between 2.13 and 3.38, and in *D. alata* between 2.31 and 3.07; the predicted triploid for the latter species had a median allelic ratio of 2.26. All the samples defined as tetraploids and two of the undefined by nQuire showed polyploid patterns based on allelic ratios (Figure 2 and Supplementary Table S4).

For some samples of *D. communis*, data from flow cytometry meant it was possible to compare the median allelic ratios with ploidy levels estimated from the 1C-values (Table 2, see boxplots labeled with an asterisk in Figure 2). Samples with estimated 1C-values indicative of triploid (0.41 pg) had a median allelic ratio of 2.50; while samples predicted to be tetraploid (range 0.49–0.59 pg) had median allelic ratios of 2.57 and 3.33; those predicted to be hexaploid with 1C-values 0.97–1.08 pg had median ratios of 2.68 and 2.90, and predicted octoploid samples with 1C-values of 1.29–1.37 pg had median ratios of 2.66, 2.73, and 2.75.

**TABLE 2:** Relationships between the allelic ratio statistics (median and quartiles) shown in Figure 2 and the known ploidy levels of *Dioscorea communis* samples based on flow cytometry and chromosome counts.

				**Allelic ratio statistics**		
**Sample code (Figure 2)**	**Chr. number (2*n*)**	**Approx. 1C (pg)**	**Ploidy**	**Median**	**1st quartile**	**3rd quartile**
S80		0.41	3x	2.0	1.4	3.0
R28		0.59	4x	2.2	1.4	3.0
R86		0.49	4x	2.5	1.8	3.5
S42		0.54	4x	2.6	2.0	3.1
R01	ca. 36	0.97	6x	2.7	1.6	3.3
R84		1.08	6x	2.7	2.2	3.5
R30		1.36	8x	2.8	2.0	3.4
R27		1.29	8x	2.8	2.0	3.4
R29		1.37	8x	2.8	2.3	3.5

**FIGURE 3:**
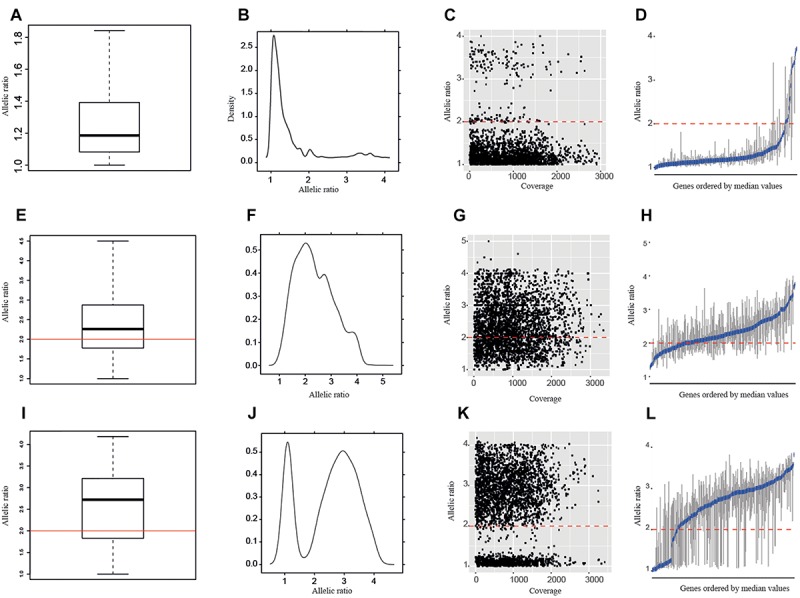
Allelic ratios of the samples represented in Figure 2: R104, a *D. sylvatica* diploid **(A–D)**; T38, a *D. alata* triploid **(E–H)**; and P06, a *D. communis* tetraploid **(I–L)** according to nQuire estimations. **(A,E,I)** Boxplots of allelic ratios per sample; **(B,F,J)** density plots of allelic ratios per sample; **(C,G,K)** allelic ratios per SNP; and **(D,H,L)** boxplots of allelic ratios per gene. Red lines represent an allelic ratio of two. Blue lines in allelic ratio per gene graphic represent the median values.

In addition to the overall boxplots and the allelic ratios calculated for each SNP position, we studied the allelic ratio distribution per gene and sample (Figure 3 and Supplementary Table S4). In all diploid samples, most of the genes showed boxplots with median values >2 and >65% of the SNPs with allelic ratios <2 (Supplementary Table S5 and Supplementary Data Sheet S1). By contrast, polyploid samples were characterized by showing a wide range of genes with median values of the allelic ratios >2 (Figure 3, Supplementary Table S5, and Supplementary Data Sheet S1) and <50% of SNPs with allelic ratios <2. The only tetraploid sample of *D. sylvatica* (R103), the sample S89 of *D. communis* for which ploidy level could not be determined by nQuire, and the sample Y09 of *D. alata*, would fit with being a recent autopolyploid in which most of the genes are still in equal proportions in the genome (e.g., 2:2 vs. 3:1 in a 4*x*), in which a high percentage of the genes showed median values below 2, and the percentage of SNPs with allelic ratios <2 was 61.7 and 59.1, respectively (Supplementary Table S5 and Supplementary Data Sheet S1).

## Discussion

Our study explored whether two approaches, flow cytometry coupled with chromosome counts and a bioinformatic pipeline that uses LSCN from HTS Hyb-Seq data, can estimate ploidy levels from a range of samples, including herbarium specimens. The effectiveness of flow cytometry was mostly limited to fresh and recently collected (and dried) material and proved to be unsuccessful in determining ploidy levels for herbarium samples older than 15 years old. Flow cytometry can be used to estimate ploidy levels from herbarium specimens collected up to fifteen years ago, but success rate is low (5.9%). The bioinformatic pipeline, based on the analysis of 260 LSCN genes, yielded ploidy information from samples collected up to 236 years ago, and provided results for 91.7% of these herbarium samples (Supplementary Table S1, results not shown). It may therefore offer novel opportunities for the use of herbarium materials in cytogenetic studies, increasing their value beyond the generation of DNA sequence data. Because this research used *Dioscorea* species as test material, the new estimates of ploidy level also substantially increased our knowledge of the diverse range of ploidy levels across three economically important yam species. This method relies on a set of LSCN genes, but could be used with other bait sets, and has some dependency on heterozygosity (i.e., percentage of polymorphic sites), but not coverage, at least for the samples covered in our study, for estimating the ploidy level of the specimens.

### Use of Flow Cytometry to Estimate Ploidy of Dried Samples Is Limited by Sample Age

Multiple studies have explored the use of various types of dried material to estimate ploidy levels in plants (e.g., Suda and Trávníček, 2006) and produced highly variable results that depend on factors such as the drying method, the age of material and the species. Recently collected plant samples that were dried in silica gel have often been shown to be suitable for ploidy analysis using flow cytometry. For example, flow cytometry was successfully used to estimate ploidy levels in all 139 leaf samples of *Gagea reticulata* (Liliaceae) from Iran that were dried in silica-gel for one year (Zarrei et al., 2012), and a similar level of success was achieved in an analysis of over 1,000 silica gel-dried leaf samples of *Oxalis obtusa* (Oxalidaceae) from the Cape Floristic Region of southern Africa that were analyzed within 2 months of being collected (Krejcíková et al., 2013).

Likewise, although to a lesser extent, the estimation of ploidy levels from herbarium material using flow cytometry has been successful for some species [e.g., six-year-old specimens of *Vaccinium* from Czechia (Suda, 2004); two-year-old specimens of *Rosa canina* from the United Kingdom, Roberts, 2007; 5.5-year-old specimens of *Festuca* from Romania, Šmarda, 2006; Šmarda and Stančík, 2006]. However, the factors influencing success rate are poorly understood and often appear to be species-dependent. Our study also showed a species-dependent pattern of flow cytometry success using herbarium material for *Dioscorea*, and these samples had higher CVs in their flow histograms compared to measures obtained from fresh material. Overall, the level of success was low, with only 5 of 85 (5.9%) herbarium samples yielding analyzable data (Table 1B and Supplementary Table S1). This may partly reflect the age (collected between 1831 and 2011) of the 85 analyzed specimens. The successful estimation of ploidy level from a *Dioscorea* herbarium sample collected in 2002 (Table 1B) represents the oldest herbarium material to yield ploidy level data using flow cytometry to date; the previous oldest herbarium material that produced analyzable flow cytometry data was a 12-year-old *Festuca* dried leaf (Šmarda, 2008).

Suda and Trávníček (2006) explored various drying approaches to mimic the preparation of herbarium samples in the field, looking at 60 species from 58 genera and 38 families across angiosperms. Flow cytometry was able to estimate ploidy in 86% of the species nine months after collection, and 72% of these still produced peaks after 20 months. The factors influencing the ability to predict ploidy after drying were however, unclear, although species known to be difficult to analyze with fresh material (e.g., those with high levels of tannin or mucilaginous compounds) also did not work well after drying. They noted that while certain types of material did not desiccate easily, such as waxy leaves, this did not necessarily mean they performed worse than other material types, like thinner leaves that dry faster. For some species the use of parts of the plant other than leaves yielded better results. For example, nuclei isolated from the stems of dehydrated species of *Pimpinella*, inflorescence apices of *Veronica* or involucral bracts for some Asteraceae were shown to give better results than nuclei extracted from leaves (Suda and Trávníček, 2006).

Our analysis on the effect of three different drying methods (silica-gel, drying by pressing at room temperature, and drying in a press in an oven at 60°C) on the ability of flow cytometry to deliver ploidy data, showed no significant differences between the methods and three months after having been dried. All three methods showed significant increases in CV values compared with fresh material (Supplementary Table S2). After 20 months, while silica-dried samples still produced analyzable results in 91.2% of the samples, herbarium dried materials mostly failed (29% oven-dried and 41% dried at room temperature). In all cases, the estimated 1C-values increased over time, although the % increase varied between treatments (i.e., silica-dried samples ±29.4%, oven-dried ±16.7% and room temperature dried ±7.9%, on average). In contrast, the increase in CV values were broadly similar between the different drying treatments (CV dried/CV fresh: silica-dried 3.3, oven-dried 2.9 and room temperature dried 3.4, on average). These results support the findings of a previous, larger study by Suda and Trávníček (2006). It is unknown whether these findings reflect, in part, the tissue preparation method used for flow cytometry. Increases in CV values have been reported in previous studies using the chopping method for preparing herbarium material, whereas the use of a bead-beating method to prepare 24 months old herbarium material of *Rosa canina* for flow cytometry analysis did not alter the CV values compared to those obtained from fresh leaves (Roberts, 2007). Such observations suggest that adopting the bead-beating approach may increase the age range of herbarium samples that are suitable for ploidy analysis by flow cytometry, at least for some species.

### Novel Uses of Herbarium Specimens: HTS-Based Ploidy Estimation

Allelic frequencies estimated from HTS data were initially used to estimate ploidy levels of cell samples from tumors in humans (e.g., Amarasinghe et al., 2014) and more recently, this approach has been applied to other model organisms like yeast (Corrêa dos Santos et al., 2017; Weiß et al., 2018). For each of these studies a tetraploid upper limit was assumed in the bioinformatic pipelines. For example, EST-SSR nuclear markers have been used to distinguish between diploid and tetraploid individuals of the fish *Misgurnus anguillicaudatus* (Feng et al., 2018) and RAD-seq data were used to explore ploidy levels in *Betula* species (Zohren et al., 2016).

Here, we showed that our HTS-based pipeline successfully distinguishes between diploids, triploids, and polyploids that are tetraploids or higher. However, it is not yet possible to determine the exact ploidy level in samples in the last category (tetraploid or higher). This is in part due to the noise associated with sequencing errors generated by the HTS platforms, which lead to the need to discard low frequency SNPs (Corrêa dos Santos et al., 2017; also see Supplementary Table S5 and Supplementary Data Sheet S1). For example, a hexaploid organism is expected to have SNPs at ratios of one (3:3), two (4:2) and five (5:1); and the latter 5:1 corresponds with an allelic frequency of 0.2 for the less frequent allele. However, the density plots shown in Supplementary Table S4 for some samples of *D. communis* (e.g., sample R75) are different from those of a “typical” tetraploid (e.g., sample P06) with peaks at allelic ratios of approximately one and four, and so higher ploidy levels are suspected. We have verified the polyploidy of the studied samples by the global average mean value of the allelic ratio, and by studying this allelic ratio by gene and by SNP (Supplementary Table S5 and Supplementary Data Sheet S1).

Both heterozygosity and coverage were previously described as key factors that shape allelic frequency distributions (Corrêa dos Santos et al., 2017), although only coverage was tested as a factor that might influence ploidy estimation parameters by nQuire (Weiß et al., 2018) using *Saccharomyces cerevisiae* and *P. infestans* whole genome sequences as models. Weiß et al. (2018) explored the threshold coverage needed to reliably predict ploidy levels using the Δlog*L* value and determined that minimum coverage should be 20–40*x*. In our study, the impact of coverage was less clear. For example, fifteen samples of *D. communis* showed coverage values below 50*x* (Supplementary Tables S3, S4), and yet only two could not be assigned a ploidy level by nQuire (S53, S61). The remaining samples each generated significantly different Δlog*L* values to support tetraploid models. In contrast, nQuire was unable to determine ploidy levels in nine samples with coverage greater than 50*x* (e.g., *D. communis* S78 with a coverage of 126*x*, Supplementary Table S3). Differences in the impact of coverage between our study and that of Weiß et al. (2018) may be because here, we use data that has been specifically enriched for LSCN genes. These are likely to include genes that are typically returned to single copy during diploidization following WGD (De Smet et al., 2013; Mandáková et al., 2017) and thus our ploidy level estimations are only likely to detect more recent polyploidization events. More ancient WGDs that have gone through extensive diploidization are unlikely to be detected by our set of LSCN genes. It is also possible that the use of LSCN could reduce the impact of low coverage on ploidy level estimations compared with studies such as Weiß et al. (2018) which used whole genomic data.

To explore the impact of heterozygosity on the ability to predict ploidy levels, we used the percentage of polymorphic sites to compare levels of heterozygosity between samples (see Methods). Using this approach, nQuire was unable to assign a ploidy level to most samples where the percentage of polymorphic sites was below 0.50. This finding supports a previous study that failed to detect a recent autopolyploid based on allelic frequencies estimated from read counts of HTS data (Zohren et al., 2016) and suggests that our approach may be unable to correctly identify recent autopolyploids that are predicted to have low percentages of polymorphic sites and equal allelic frequencies compared with older, more divergent autopolyploids and allopolyploids. One further factor that might limit the ability of our approach to assign ploidy levels is the presence of aneuploidy. Currently this can only be estimated if reads can be mapped onto a fully assembled reference genome (Weiß et al., 2018).

The presence of numerous WGD events throughout the evolutionary history of land plants (Van de Peer et al., 2017) make it challenging to identify potentially orthologous nuclear genes to reconstruct phylogenetic relationships or for mapping molecular markers (Duarte et al., 2010). One approach to overcome the problems associated with the presence of paralogous genes that can generate misleading topologies (due to misinterpretation of orthology) that do not reflect species relationships, is to identify sets of LSCN genes for phylogenetic studies (e.g., Wickett et al., 2014). Indeed, a growing number of studies have characterized and used LSCN genes for different groups, e.g., across all seed plant lineages (Li et al., 2018), and angiosperm-wide (De Smet et al., 2013), and there are now several target-capture kits which have been developed to sequence LSCN genes in plants (e.g., Johnson et al., 2018). Our approach has the potential to identify multiple copy genes in species for which a maximum of two copies per specimen are expected (i.e., diploids) by calculating the median value of the allelic frequencies for each gene.

### Large Ploidy Variation Is Found Among and Within *Dioscorea* Species

*Dioscorea* shows a diverse range of ploidy levels both among and within species (Tables 1, 2). Understanding such diversity is important, as it has implications for plant conservation and the utility of plant collections for cross-compatibility and for breeding improved varieties. Our estimates of ploidy level in multiple samples of the three focal species corroborate and extend previous studies, highlighting the existence of two extremes in ploidy diversity between *Dioscorea* species. Some species studied here had mostly homogeneous ploidy levels, at least for the material examined (e.g., South African *D. sylvatica*, where nearly all individuals were estimated to be diploid), while others showed high levels of ploidy variation (e.g., Mediterranean *D. communis*, where diploids were in the minority, and ploidy levels of at least 8*x* were predicted) (Figure 2 and Supplementary Table S2).

The flow cytometry analysis of the three fresh samples of *D. sylvatica* gave a mean estimated 1C-value of 0.47 pg (Table 1) indicative of a diploid ploidy level. Although this value is somewhat lower than a previous genome size estimate 0.85 pg/1C by Bharathan et al. (1994), it is noted that no chromosome count could be obtained in either study. Thus, it is possible that the sample analyzed by Bharathan et al. was a tetraploid, as also predicted for one of the samples here (R103). Using the HTS-based approach (Supplementary Table S3) 25 out of 26 herbarium samples were predicted to be diploid. Notably, both flow cytometry (Table 1) and HTS-based estimations of the same sample (i.e., RBGKLiv2011-249 = S49) predicted it to be diploid, providing confidence in the ability of the Hyb-Seq approach to predict ploidy level.

Defining the ploidy level of *D. communis* has been unclear. Chromosome counts of this species (2*n* = 48, Al-Shehbaz and Schubert, 1989) imply it is a tetraploid based on *x* = 12, but the allopolyploid patterns observed using microsatellite (simple sequence repeat; SSR) markers in the sister group *Borderea* suggest that *D. communis* could also be considered an octoploid based on *x* = 6 (Segarra-Moragues et al., 2003). Similarly, the Macaronesian sister species *D. edulis* was reported as octoploid based on chromosome counts (2*n* = 96), and 16-ploid based on SSR-based estimation (Segarra-Moragues et al., 2003).

For one sample of *D. communis* in our study (R01) we obtained (a) a chromosome count of 2*n* = ca. 36, (b) an estimated 1C-value of 0.97 pg, and (c) a median allelic ratio of 2.7 using the HTS data (Table 2) leading us to suggest that this sample is hexaploid. We then used this as a reference to determine the ploidy levels of all the other samples, and report for the first time the existence of multiple ploidy levels within *D. communis* (Figure 2). Seven samples fit a diploid pattern (Figure 2), and the origin of these samples suggest there is a strong geographic signal in the distribution of diploid cytotypes. Most of the remaining samples are polyploids. We will explore the phylogeography and the ploidy variability across the Mediterranean for the *Tamus* group in further studies. Nevertheless, our study highlights the challenge of defining what a polyploid is given the contrasting information provided by genome size, chromosome number, meiotic behavior, and genetic and evolutionary data.

Because of its economic importance, several studies have explored genome size variation in *D. alata* using flow cytometry and uncovered a large variation of 1C-values ranging from 0.45 to 1.30 pg/1C (Arumuganathan and Earle, 1991; Hamon et al., 1992; Marie and Brown, 1993; Dansi et al., 2005; Arnau et al., 2009; Obidiegwu et al., 2009). Such genome size diversity is consistent with the many different chromosome counts reported for this species, ranging from 2*n* = 20 to 80, in multiples of 10 (reviewed in Viruel et al., 2008).

As for *D. communis*, there is also a lack of clarity in the literature regarding the ploidy level of *D. alata*, in which the 2*n* = 40 cytotypes have been recognized as diploids (i.e., *x* = 20, instead of *x* = 10) based on SSR marker patterns (Arnau et al., 2009). Nevertheless, chromosome counts of 2*n* = 20 have been reported (Martin and Ortiz, 1963), indicating that samples with 2*n* = 40 should be regarded as tetraploids. The genome size of 2*n* = 40 cytotypes ranged from 0.5–0.6 pg/1C and the 2*n* = 80 cytotypes were 1.06 pg/1C (Dansi et al., 2005). Our HTS-based procedure generated tetraploid patterns in samples matching a 2*n* = 40 cytotype (i.e., an estimated 1C-value of 0.56 and 0.59, Table 1). We therefore consider that the previously reported 2*n* = 40 cytotypes (ranging from 0.5–0.6 pg/1C) are cytologically tetraploids, based on *x* = 10. Nevertheless, a recent study describing a reference high-density genetic map for *D. alata* (Cormier et al., 2019) – obtained from crosses of a female breeding line (74F, 1C = 0.51 pg), and a male Caribbean landrace (Kabusa; 1C = 0.43 pg), both with genome size suggestive of 2*n* = 40 – proposed that the samples analyzed were genetically diploid. For example, the number of linkage groups obtained ranged from 21 to 26, which does not correspond to a chromosome base number of *x* = 10. Autotetraploid specimens can show biallelic genotypes that mask their cytological polyploid status (e.g., with SSR and SNP markers), and as shown for some *Betula* samples (Zohren et al., 2016), while ploidy estimates based on allelic frequencies (as used here) reveal the ploidy level predicted from the chromosome counts.

The frequency of different ploidy levels in *D. alata* seems to vary depending on the origin of the samples studied. For example, Egesi et al. (2002) used flow cytometry to analyze 53 accessions, mainly from West Africa, and found that most (84.9%) were hexaploid, while all remaining samples (i.e., 15.1%) were tetraploid. Girma et al. (2017) also used flow cytometry to analyze 139 accessions from West Africa and found that a slim majority were octoploid (53%), followed by tetraploids (43%), and a few hexaploids (3%). A broad geographical analysis of 384 *D. alata* accessions from the South Pacific, Asia, Africa, and the Caribbean found considerable geographical structure in the distribution of tetraploids, hexaploids, and octoploids in this species, with tetraploids and octoploids being more widely distributed and more common than hexaploids (Arnau et al., 2017). Our results confirm that polyploid forms are frequent in *D. alata* based on both flow cytometry (Table 1) and our HTS-based estimations (Supplementary Table S3), at least for the predominantly Asian samples analyzed here. Thus, due attention to ploidy determination is recommended in breeding and genomic studies of this species.

### Applicability of the Hyb-Seq Approach to Other Eukaryotic Lineages

Since coverage appears to be less of an issue when using HTS data generated from a targeted baiting approach to infer ploidy (see above), this approach has the potential to be applied to the growing number of plant groups where HybSeq methods and bait kits are being applied (e.g., Villaverde et al., 2018).

Whole genome duplications have likely played a major role in the evolution of other lineages, including insects (Li et al., 2015b, 2019; Nakatani and McLysaght, 2019), teleost fish (Berthelot et al., 2014) and diatoms (Parks et al., 2018). Due to the growing availability of genomic resources for these groups, the approaches outlined here could also be applied to explore polyploid evolution using phylogenomic data compiled to reconstruct the tree-of-life. Since our pipeline detects recent polyploidization events and the ploidy level estimation is comparable among related species, it is envisaged that it could also be complemented by other genomic methods focused on detecting more ancient polyploidization events (e.g., Vanneste et al., 2015; Roodt et al., 2017; Qiao et al., 2019). Currently, there is limited understanding of the frequency and impact of polyploidy in fungi (Albertin and Marullo, 2012; Campbell et al., 2016; Todd et al., 2017). While recent polyploid events have been documented across the fungal tree of life, robust evidence for the existence of ancient WGDs is currently limited to the yeast genus *Saccharomyces* (estimated to have occurred ca. 100 Mya; Wolfe and Shields, 1997; Campbell et al., 2016) and the saprophytic fungus *Rhizopus* (reviewed in Campbell et al., 2016). In part, our limited knowledge of polyploidy in fungi is hampered by the lack of genomic data, but this is likely to change with the rapid growth of whole genome sequence data for fungi (Leitch et al., 2018). Such data are likely to be suitable for applying the approaches outlined in this paper, including data generated from fungarium samples, given the ability to extract and sequence DNA from such voucher specimens (Dentinger et al., 2016). Such approaches should therefore considerably extend the ability to explore whether polyploidy is indeed more common in fungi than currently recognized.

The methods presented here are likely to be of interest for many plant groups, particularly those where polyploidy is common. For example, Wood et al. (2009) reviewed the extent of recent polyploidization throughout the angiosperms, and highlighted genera with a high incidence of WGD (measured as the percentage of polyploid species calculated using the number of species with chromosome count data): 92.3% in *Perideridia* (Apiaceae), 95% in *Senecio* (Asteraceae), 91.4% in *Campanula* sect. *Isophylla* (Campanulaceae), 96.7% in *Cerastium* (Caryophyllaceae), 88.9% in *Aponogeton* (Aponogetonaceae), 92.3% in *Alpinia* (Zingiberaceae) and 80% in *Geum* allies (Rosaceae).

Documenting polyploidy in herbarium samples is likely to have applications in plant breeding and horticulture. For example, many popular commercial hybrids of the orchid genus *Phalaenopsis* are tetraploids (Chen et al., 2014) yet many of these hybrids have a narrow genetic base, which makes it difficult to transfer favorable genes from diploid wild species to commercial hybrids because of the difference in ploidy levels. Hybrids produced by artificial crosses between these species are usually sterile. Estimating the ploidy level of herbarium samples will provide new and useful information to orchid breeders for selecting promising parental varieties to accelerate the breeding of novel varieties. Similarly, it will clarify the contribution of recent polyploidy events to the intraspecific phenotypic variation of *Vanilla planifolia* accessions (Bory et al., 2008).

Enormous interest exists on revealing the ploidy levels of lineages where gametophytes are the dominant life phase as, for example, in the haplodiploid life cycle of many algae (Klinger, 1993) and since some polyploidization events have been reported in most major groups of algae (Nichols, 1979). Indeed, unusual polyploidization forms have been proposed within the life cycle of some algal species estimated through flow cytometry and microsatellite markers (Varela-Álvarez et al., 2018). Several attempts have been made to optimize ploidy estimation with dried material using flow cytometry (e.g., for fungi, Todd et al., 2018) or tyramide signal amplification-fluorescence *in situ* hybridization (TSA-FISH) for LSCN (e.g., alga, Vazac et al., 2018) for different organisms. Our approach provides a more straightforward way to estimate ploidy levels, especially with material obtained from herbarium samples.

## Conclusion

Novel HTS techniques have revolutionized the amount of genomic resources available for non-model organisms and enabled the generation of genomic data from degraded DNA samples (e.g., Besnard et al., 2014; Hart et al., 2016) that would not be available using more traditional molecular approaches such as PCR. It is particularly noteworthy that the bioinformatic pipeline optimized here demonstrates that it is now possible to efficiently gain insights into the ploidy status of a species even from herbarium samples up to two centuries old for which flow cytometry and/or chromosome counts are not possible. Our study therefore provides a further demonstration of the vital importance of herbaria as a rich source of material to provide novel insights into the genomic make up of plants that were previously intractable.

## Data Availability

The datasets generated for this study can be found in SRA repository, PRJNA525269.

## Author Contributions

JV, PW, IL, FF, BG, MSG, MK, and SG secured the funding for the project. LP advised on methods and analyses. JV, MSG, MC, OH, and RP conducted the experimental work. JV conducted the bioinformatics analyses. All authors contributed to writing and revising the manuscript.

## Conflict of Interest Statement

The authors declare that the research was conducted in the absence of any commercial or financial relationships that could be construed as a potential conflict of interest.
