# Supplementary material for: A Target Capture-Based Method to Estimate Ploidy From Herbarium Specimens
Source: Front Plant Sci. 2019 Jul 24;10:937. doi: 10.3389/fpls.2019.00937 (PMC6667659; doi:10.3389/fpls.2019.00937)
Supplement: Supplementary file 1 [file Table_1.pdf]

Supplementary Table 1. List of the 85 herbarium samples analysed from the Kew and Leiden herbaria to test whether flow cytometry was able to estimate ploidy level. The five species that yielded sufficiently good flow cytometry data to estimate ploidy are in bold (see Table 1B). Many of these species were also used to test the robustness of the Hyb-Seq approach to estimate ploidy. This is indicated in the column labelled Hyb-Seq, Y: Yes - same species and accession used, N: No - same species and accession did not produce high quality sequencing results; * used other specimen of the same species in Hyb-Seq.

Code	Species	Collector	Collection number	Locality	Date collected	K barcode	Hyb Seq
m105	*D. bridgesii*	Cruickshanks	173 bis	Chile	<1867	K001150655	Y
m210	*D. blumei*	H. Reinwardt	s.n.	?	1831	L.1471472	Y
m214	*D. wrightii*	Herb Wright	s.n.	Malaysia	1866	L.1465560	Y
m367	*D. sericea*	André	1790	Colombia	1876	K001150233	Y
m376	*D. ternata*	s. coll.	14348	Brazil	1882	K001149756	Y
m370	*D. mandonii*	Bang	1296	Bolivia	1891	K001150271	Y
m217	*D. madiuensis*	Koorders	29205	Java	1897	L.1464939	Y
m269	*D. chondrocarpa*	Williams	464	Bolivia	1901	K001150537	Y
m381	*D. psilostachya*	Britton	230	Cuba	1903	K001149319	N
m206	*D. merillii*	Elmer	193	Philippines	1909	L.1465047	N
m267	*D. demourae*	Weberbauer	7862	Peru	1929	K001150388	*
m178	*D. zingiberensis*	Wang	22659a	China	1930	K001141304	Y
m196	*D. fordii*	Tsang	27025	Vietnam	1936	K001144012	*
m316	*D. guianensis*	Sandwith	1436	Guyana	1937	K001149591	Y
m108	*D. obtusifolia*	Worth	16112	Chile	1938	K001150666	Y
m306	*D. cyanisticta*	Skutch	4221	Costa Rica	1939	K001149023	Y
m271	*D. multiflora*	Haught	2781	Colombia	1939	K001150600	*
m375	*D. tamoidea*	Morton	3440	Cuba	1941	K001149234	Y
m215	*D. esculenta*	Taleon	38847-2	Philippines	1954	L.1471109	Y
m221	*D. grata*	Sulit	5475	Philippines	1954	L.1470982	N
m177	*D. zentaroana*	Creech	148	Japan	1958	K001141389	Y
m364	*D. linearicordata*	Tsang	26780	China	1963	K001141814	*
m317	*D. hirtiflora* subsp. *hirtiflora*	Adames	470	Liberia	1964	K001145640	Y
m277	*D. acanthogene*	Prance	5163	Brazil	1968	K001140150	*
m205	*D. alata*	Henty	41956	Papua N Guinea	1969	L.1471533	Y
m270	*D. incayensis*	Lugo	1126	Ecuador	1969	K001171665	Y
m074	*D. belophylla*	Malhotra	42644	India	1970	K001142803	*
m390	*D. faringdomiana*	Bosser	19984	Madagascar	1970	P00231412(K)	Y
m374	*D. mangenotiana*	Wit	33	Nigeria	1971	K001145992	Y
m106	*D. saxatilis*	Mahu	9633	Chile	1973	K001150661	Y
m274	*D. nitida*	Hatschbach	37332	Brazil	1975	K001149640	*
m373	*D. syringifolia*	Lindeman	651	Suriname	1975	K001149699	Y
m383	*D. macrothyrsa*	Harley	18107	Brazil	1977	K001149987	*
m216	*D. fordii*	K'Tung	78	China	1978	L.1471021	Y
m025	*D. keduensis*	Vogel	6187	Indonesia	1979	K001144217	Y
m207	*D. kjellbergii*	Balgooy	3734	Indonesia	1979	L.1465047	N
m098	*D. quinquefolia*	Leiva	48335	Cuba	1982	K001149402	Y
m295	*D. cinnamomifolia*	Chautems	89	Brazil	1983	K001150207	*
m382	*D. pynaertii*	Thomas	7197	Cameroon	1987	K000809884	Y
m114	*D. andina*	Gardner	4029	Chile	1988	K001150713	Y
m046	*D. esculenta*	Hopkins	2030	Papua N Guinea	1990	K001144935	Y
m281	*D. grandiflora*	Lughadha	50575	Brazil	1991	K001149723	Y
m302	*D. loheri*	Stone	434	Philippines	1991	K001144898	Y
m229	*D. plumifera*	Vazquez	1370	Mexico	1991	K001149019	Y
m058	*D. gracillima*	Nie	92143	China	1992	K001141376	Y
m368	*D. martiana*	Kallunki	324	Brazil	1993	K001149909	Y
m225	*D. matagalpensis*	Fuentes	591	Costa Rica	1993	K001149034	Y
m200	*D. katrica*	Boyce	898	Thailand	1994	K001144001	Y
m200	*D. kratica*	Boyce	898	Thailand	1994	K001144001	Y
m158	*D. lanata*	Thulin	8792	Yemen	1994	K001141166	Y
m366	*D. marginata*	Guedes	1151	Brazil	1994	K001149791	*
m380	*D. pilosiuscula*	Espinosa	MB489	Panama	1994	K001150238	Y
m333	*D. sambiranensis* subsp. *bardotiae*	Lewis	1134	Madagascar	1994	K000524246	Y
m296	*D. coriacea*	Lewis	2849	Ecuador	1996	K001150191	Y
m384	*D. melastomatifolia*	Costa	719	Brazil	1996	K001149785	Y
m273	*D. rumicoides*	Wood	10997	Bolivia	1996	K001150447	*

m328	*D. tricantha*	Caddick	308	Madagascar	1997	K001148373	Y
m265	*D. kamoonensis*	Wilkin	997	Thailand	1998	K001143800	Y
m337	*D. bako*	Wilkin	1145	Madagascar	2000	K000523601	N
m325	*D. bosseri*	Wilkin	1144	Madagascar	2000	K000523659	Y
m239	*D. mexicana*	McPherson	20536	Panama	2000	K001150578	Y
m329	*D. sterilis*	Wilkin	1150	Madagascar	2000	K001148388	*
m199	*D. kimiae*	Poncy	1535	Madagascar	2001	K000523541	Y
m334	*D. sambiranensis* subsp. *ambrensis*	Block	1331	Madagascar	2001	K000523590	Y
m343	*D. bemandry*	Rajaonah	MT605	Madagascar	2002	K001171776	Y
m309	*D. hispida*	Medum	115	Indonesia	2002	K001144379	*
**m109**	***D. modesta***	**Casado**	**INIA 015**	**Chile**	**2002**	**K001150677**	**Y**
**m389**	***D. ‘ovy-valiha’***	**Ranirison**	**714**	**Madagascar**	**2004**	**K000523550**	**Y**
m356	*D. trifida*	Granville	16363	French Guiana	2004	K001149766	N
m339	*D. orangeana*	Randrianaivo	1148	Madagascar	2005	K000523812	Y
m345	*D. hombuka*	Antilahimena	4869	Madagascar	2006	K001171807	*
m377	*D. therezopolensis*	Paula-Souza	5890	Brazil	2006	K001149929	Y
m365	*D. tsaratananensis*	Rakotovao	3424	Madagascar	2006	K000062189	Y
m392	*D. decaryana*	Ralimanana	890	Madagascar	2008	(K)	Y
**m320**	***D. mollis***	**Ranarivelo**	**RLI952**	**Madagascar**	**2008**	**K000062164**	**Y**
**m371**	***D. trilinguis***	**Zappi**	**953**	**Brazil**	**2008**	**K000579751**	**Y**
m331	*D. heteropoda*	Ralimanana	1141	Madagascar	2009	K000312425	Y
m301	*D. nitida*	Wood	26327	Bolivia	2009	K001150251	Y
m346	*D. dregeana*	Lukhele	UPP104	South Africa	2010	K000984849	Y
m319	*D. calvescens*	Gautier	LG5568	Madagascar	2011	K00062192	Y
**m293**	***D. multiflora***	**Iganci**	**813**	**Brazil**	**2011**	**K001171677**	*****
m393	*D. acuminata*	s. coll.	1623	Madagascar	?	(K)	N
m294	*D. ovata*	?	?	?	?	K001274736	*
m391	*D. rakotonasoloi*	Wilkin	1682	Madagascar	?	(K)	Y
