# Supplementary material for: A Target Capture-Based Method to Estimate Ploidy From Herbarium Specimens
Source: Front Plant Sci. 2019 Jul 24;10:937. doi: 10.3389/fpls.2019.00937 (PMC6667659; doi:10.3389/fpls.2019.00937)
Supplement: Supplementary file 2 [file Table_2.pdf]

Supplementary Table 2. Effect of different drying techniques on the ability to estimate ploidy levels in *Dioscorea* species. Material refers to fresh (F) or dried samples, the latter in silica (S), oven (O) or herbarium dried at room temperature (H) followed by the number of months since it was dried (3 and 20) - see Materials and Methods for details. Chromosome numbers are taken from Viruel et al. (2008) and references therein. Clade names follow Viruel et al. (2016): CL = Compound leaf, NW1 and II = New World 1 and II.

Species	Sample information	Material	CV sample	Increase ratio CV	CV standard	Approx. 1C (pg)	% change
D. alata L.	RBGKLiv 1982-1316	F	3.39		2.77	0.84	
	"	S - 20	14.78	4.4	2.02	1.12	33.3
	"	O - 20	-	-	-	-	-
	"	H - 20	22.00	6.5	2.88	1.03	22.6
	RBGKLiv PalmH. (R89)	F	4.55		3.53	0.56	
	"	S - 20	8.68	1.9	1.73	0.69	23.2
	"	O - 20	-	-	-	-	-
	"	H - 20	-	-	-	-	-
	RBGKLiv 1987-1993	F	4.51		3.48	0.59	
	"	S - 20	11.70	2.6	1.83	0.70	18.6
	"	O - 20	5.42	1.2	1.86	0.57	3.40
	"	H - 20	-	-	-	-	-
D. altissima Lam.	RBGKLiv 2005-1233	F	4.50		3.76	0.68	
	"	S - 20	7.24	1.6	2.02	0.57	16.2
	"	O - 20	-	-	-	-	-
	"	H - 20	13.43	3.0	1.65	0.79	16.2
D. antaly Jum. & H.Perrier	RBGKLivMSB-406347	F	5.44		1.93	0.33	
	"	S - 20	24.72	4.5	1.71	0.42	27.3
	"	O - 20	-	-	-	-	-
	"	H - 20	14.31	2.6	2.02	0.57	72.7
	RBGKLiv 1998-523	F	3.44		1.79	0.31	
	"	S - 20	21.65	6.3	1.42	0.53	71.0
	"	O - 20	-		-	-	
	"	H - 20	21.01	6.1	2.41	0.40	29.0
	RBGKLiv 2014-641	F	4.23		2.48	0.33	
	"	S - 20	-	-	-	-	-
	"	O - 20	-	-	-	-	-
	"	H - 20	-	-	-	-	-
D. bulbifera L.	RBGKLiv JodrellN	F	4.48		1.83	0.36	
	"	S - 3	15.00	3.3	3.50	0.40	11.1
	"	O - 3	20.40	4.6	3.30	0.47	30.6
	"	H - 3	20.60	4.6	4.00	0.53	47.2
	"	S - 20	13.29	3.0	1.61	0.40	11.1
	"	O - 20	18.09	4.0	1.89	0.45	25.0
	"	H - 20	-	-	-	-	-
	RBGKLiv 2000-2561	F	2.18		2.47	1.57	
	"	S - 20	13.01	6.0	1.18	1.85	17.8
	"	O - 20	9.99	4.6	1.96	1.77	12.7
	"	H - 20	7.94	3.6	2.68	1.87	19.1
D. caucasica Lipsky	RBGKLiv 2014-1847	F	5.02		2.78	0.51	
	"	S - 20	15.19	3.0	0.82	1.85	262.7
	"	O - 20	-	-	-	-	-
	"	H - 20	-	-	-	-	-
D. cayenensis subsp. *rotundata* (Poir.) J.Miège	RBGKLiv 1920-76.01470	F	3.00		2.20	0.72	
	"	S - 20	11.60	3.9	1.64	0.85	18.1
	"	O - 20	-	-	-	-	-
	"	H - 20	-	-	-	-	-
D. composita Hemsl.	RBGKLiv 1969-11715	F	2.89		1.80	0.47	
	"	S - 20	18.37	6.4	1.67	0.54	14.9
	"	O - 20	19.32	6.7	1.77	0.57	21.3
	"	H - 20	9.69	3.4	1.44	0.60	27.7
	RBGKLiv 1978-1830	F	2.37		1.60	0.48	

	“	S - 20	15.88	-	2.06	0.52	8.3
	“	O - 20	-	-	-	-	-
	“	H - 20	-	-	-	-	-
D. communis (L.) Caddick & Wilkin	RBGKLiv JLMN182	F	4.45		2.92	0.49	
	“	S - 20	14.74	3.3	2.43	0.67	36.7
	“	O - 20	-	-	-	-	-
	“	H - 20	8.68	2.0	2.06	0.58	18.4
D. deltoidea Wall. ex Griseb.	RBGKLiv 1963-26702	F	4.30		2.70	0.65	
	“	S - 20	12.43	2.9	1.68	0.68	4.6
	“	O - 20	19.22	4.7	2.07	0.78	20.0
	“	H - 20	16.05	3.7	2.30	0.77	18.5
D. dumetorum (Kunth) Pax	RBGKLiv 1984-8045b	F	6.80		3.40	0.41	
	“	S - 20	17.24	2.5	1.73	0.44	7.3
	“	O - 20	-	-	-	-	-
	“	H - 20	-	-	-	-	-
	RBGKLiv 1984-8045	F	6.00		3.50	0.41	
	“	S - 20	14.10	2.4	1.20	0.44	7.3
	“	O - 20	-	-	-	-	-
	“	H - 20	-	-	-	-	-
D. glabra Roxb.	RBGKLiv 1996-4312	F	3.80		3.5	0.69	
	“	S - 3	-		-	-	
	“	O - 3	19.00	5.0	3.20	0.81	17.4
	“	H - 3	20.20	5.3	4.60	0.70	1.5
	“	S - 20	16.16	4.3	2.03	1.30	88.4
	“	O - 20	-	-	-	-	-
	“	H - 20	6.77	1.8	1.90	0.59	14.5
D. membranacea Pierre ex Prain & Burkill	RBGKLiv 1998-4294	F	4.80		3.30	0.86	
	“	S - 3	8.92	1.9	2.10	0.88	2.3
	“	O - 3	13.70	2.9	4.40	0.99	15.1
	“	H - 3	11.90	2.5	2.50	0.82	4.7
	“	S - 20	12.25	2.6	1.55	0.93	8.1
	“	O - 20	-	-	-	-	-
	“	H - 20	9.43	2.0	1.88	0.98	13.9
	RBGKLiv 1998-4292	F	5.50		3.60	0.81	
	“	S - 20	11.52	2.1	2.06	0.90	11.1
	“	O - 20	15.31	2.8	2.16	1.05	29.6
	“	H - 20	-	-	-	-	-
D. minutiflora Engl.	RBGKLiv 1960-1001	F	3.90		1.90	0.64	
	“	S - 20	14.40	3.7	1.64	0.71	10.9
	“	O - 20	-	-	-	-	-
	“	H - 20	-	-	-	-	-
D. pentaphylla L.	RBGKLiv Jod	F	3.00		2.90	1.14	
	“	S - 3	15.40	5.1	8.50	1.23	7.9
	“	O - 3	-	-	-	-	-
	“	H - 3	-	-	-	-	-
	“	S - 20	10.63	3.5	3.76	1.41	23.7
	“	O - 20	8.82	2.9	2.63	1.36	19.3
	“	H - 20	-	-	-	-	-
	RBGKLiv 1996-4313 Jod	F	4.60		3.40	1.29	
	“	S - 3	-	-	-	-	-
	“	O - 3	8.60	1.9	3.10	1.22	5.4
	“	H - 3	16.70	3.6	5.10	1.21	6.2
	“	S - 20	7.40	1.6	2.73	1.41	9.3
	“	O - 20	-	-	-	-	-
	“	H - 20	-	-	-	-	-
	RBGKLiv 1996-4313 TN	F	4.20		3.00	1.15	
	“	S - 20	10.72	2.6	7.78	1.11	3.5
	“	O - 20	-	-	-	-	-
	“	H - 20	14.30	3.4	2.51	1.03	10.4
	RBGKLiv 1996-4313bis TB	F	3.70		2.60	1.19	
	“	S - 20	14.61	3.9	2.15	1.30	9.2

	“	O - 20	-	-	-	-	-
	“	H - 20	9.63	2.6	2.74	1.10	8.6
D. praehensilis Benth.	RBGKLiv 1960-1002	F	3.30		2.80	0.62	
	“	S - 20	10.68	3.2	1.67	0.77	24.2
	“	O - 20	15.18	4.6	1.79	0.67	8.1
	“	H - 20	-	-	-	-	-
D. preussii Pax	RBGKLiv 1968-57006	F	3.70		3.60	1.14	
	“	S - 20	15.43	4.2	2.45	1.51	32.5
	“	O - 20	-	-	-	-	-
	“	H - 20	-	-	-	-	-
D. polystachya Turcz.	RBGKLiv Jod	F	4.30		3.70	1.75	
	“	S - 3	-	-	-	-	-
	“	O - 3	-	-	-	-	-
	“	H - 3	-	-	-	-	-
D. rockii Prain & Burkill	RBGKLiv 1996-4307	F	4.60		2.30	0.92	
	“	S - 3	9.83	2.1	3.10	0.89	3.3
	“	O - 3	16.07	3.5	3.30	0.94	2.2
	“	H - 3	11.60	2.5	3.70	0.98	6.5
	“	S - 20	7.29	1.6	1.56	1.04	13.0
	“	O - 20	22.63	4.9	1.77	0.80	13.0
	“	H - 20	-	-	-	-	-
D. sansibarensis Pax	RBGKLiv 1969-5387	F	6.90		3.50	0.36	
	“	S - 20	-	-	-	-	-
	“	O - 20	-	-	-	-	-
	“	H - 20	-	-	-	-	-
D. soso Jum. & H.Perrier complex	RBGKLiv 2014-1312	F	4.60		2.20	1.04	
	“	S - 20	8.41	1.8	2.37	1.11	6.7
	“	O - 20	-	-	-	-	-
	“	H - 20	-	-	-	-	-
	RBGKLiv 2008-3097C	F	4.10		1.90	1.24	
	“	S - 20	-	-	-	-	-
	“	O - 20	-	-	-	-	-
	“	H - 20	-	-	-	-	-
	RBGKLiv 2008-3097D	F	3.20		2.50	1.22	
	“	S - 20	-	-	-	-	-
	“	O - 20	-	-	-	-	-
	“	H - 20	-	-	-	-	-
	RBGKLiv 2005-1802b	F	5.20		4.60	0.61	
	“	S - 20	18.67	3.6	1.88	0.74	21.3
	“	O - 20	-	-	-	-	-
	“	H - 20	-	-	-	-	-
	RBGKLiv 2005-1802	F	4.70		3.20	0.69	
	“	S - 20	14.81	3.2	2.03	0.75	8.7
	“	O - 20	10.50	2.2	2.14	0.79	14.5
	“	H - 20	22.17	4.7	2.14	1.03	49.3
D. sylvatica Eckl.	RBGKLiv 2011-447 (S49)	F	7.10		4.44	0.51	
	“	S - 20	13.79	1.9	3.51	0.59	15.7
	“	O - 20	-	-	-	-	-
	“	H - 20	10.40	1.5	1.97	0.56	8.9
