# Supplementary material for: A Target Capture-Based Method to Estimate Ploidy From Herbarium Specimens
Source: Front Plant Sci. 2019 Jul 24;10:937. doi: 10.3389/fpls.2019.00937 (PMC6667659; doi:10.3389/fpls.2019.00937)
Supplement: Supplementary file 3 [file Table_3.pdf]

Supplementary Table 3. Ploidy estimation obtained by analyses of Hyb-Seq data using the nQuire pipeline (see Materials and Methods) for 27 samples of *Dioscorea sylvatica*, 56 of *D. communis* and 10 of *D. alata*. The sample information includes the collector and the barcode information for the herbarium specimens. The target-enrichment success is expressed as the total bp recovered (bp recov.) and the percentage of the total bp length (%) expected for the 260 single or low copy nuclear genes. The nQuire pipeline calculates the coverage (Cov.), while percentage of polymorphic sites (P) was calculated as the coefficient between the SNPs and the total bp recovered for each sample. The log*L* values were calculated for the free model (log*L* free) and the three ploidy models (M) implemented in nQuire: diploid (2x), triploid (3x) and tetraploid (4x). The fit of these three models to the data was estimated through delta log-likelihood (*Δ*log*L*) estimations against the free model. The fit between ideal and empirical histograms for each sample and model was characterized by a low sum of squared residuals (SSR) value, a positive slope (y-y slope) with low standard error (std.Err), and a high R^2. Values that matched with a model are shown in bold. When a sample was concluded to fit to a model, it was coloured in blue, for diploids; orange, for triploids; and green, for tetraploids; samples for which it was not possible to assign a particular ploidy level are black.

Code	Species	Sample information (year collected)	bp recov.	%	SNPs	Cov.	P (%)	log*L* free	M	log*L*	*Δ*log*L*	SSR	y-y slope	std.Err	R^2	Histogram
Z59	*D. sylvatica*	Muasya 6733	411819	95.4	2048	407	0.50	4445.9	**2x**	4136.3	**309.6**	**0.004**	**0.723**	**0.025**	**0.936**	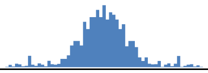
		(1998)							3x	1399.1	3046.7	0.063	-0.585	0.119	0.289	
									4x	2587.4	1858.5	0.027	0.201	0.219	0.014	
R103	*D. sylvatica*	Blatt 4	367074	85.0	1269	177	0.35	1486.8	2x	654.8	831.9	0.038	0.118	**0.055**	0.072	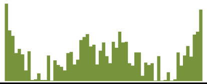
		K000728038							3x	647.9	838.9	0.043	-0.438	0.060	0.468	
		(1947)							**4x**	897.8	**589.0**	**0.011**	**0.230**	0.126	0.053	
R104	*D. sylvatica*	Wilson & Buncher	394536	91.4	2338	490	0.59	5297	**2x**	4552.5	**745.0**	**0.002**	**0.781**	**0.017**	**0.970**	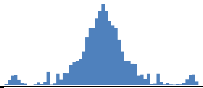
		80							3x	1563.8	3733.7	0.067	-0.615	0.127	0.283	
		K000728079 (1958)							4x	3161.5	2136.0	0.028	0.306	0.230	0.029	
R106	*D. sylvatica*	Codd 2319	356370	82.5	1430	252	0.40	2569.3	**2x**	2337.2	**232.0**	**0.007**	**0.619**	**0.025**	**0.908**	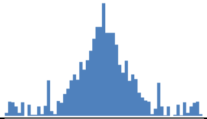
		K000728090							3x	946.1	1623.1	0.053	-0.478	0.106	0.256	
		(1946)							4x	1525.4	1043.9	0.022	0.129	0.190	0.007	
R107	*D. sylvatica*	Pole-Evans 4855	397473	92.0	1627	555	0.41	4117.5	**2x**	3659.8	**457.7**	**0.003**	**0.904**	**0.036**	**0.913**	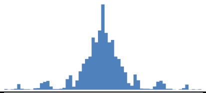
		K001148746							3x	1115.1	3002.3	0.077	-0.592	0.161	0.184	
		(1954)							4x	2601.3	1516.1	0.036	0.461	0.272	0.046	
R111	*D. sylvatica*	Strey 8329	341484	79.1	1710	227	0.50	3382.6	**2x**	3081.9	**300.7**	**0.006**	**0.653**	**0.023**	**0.930**	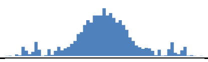
		K000728124							3x	1162.1	2220.5	0.056	-0.495	0.111	0.252	
		(1969)							4x	1950.8	1431.7	**0.022**	0.210	0.198	0.018	
R112	*D. sylvatica*	Wilson & Buncher	366036	84.8	2146	289	0.59	4542.7	**2x**	4060.4	**482.4**	**0.004**	**0.717**	**0.023**	**0.941**	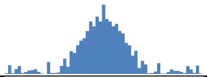
		188							3x	1438.9	3103.8	0.063	-0.605	0.115	0.315	
		K000728128 (1958)							4x	2688.8	1853.9	0.026	0.226	0.216	0.018	
R114	*D. sylvatica*	Tinley 539	342528	79.3	2369	125	0.69	3554.2	**2x**	3287.7	**266.6**	**0.014**	**0.453**	**0.027**	**0.817**	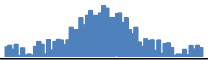
		K000728141							3x	1561.0	1993.2	0.041	-0.338	0.084	0.214	
		(1959)							4x	1978.7	1575.5	**0.019**	-0.082	0.147	0.005	

R117	*D. sylvatica*	Wilson & Buncher 3 K000728147 (1958)	330576	76.6	2151	111	0.65	2405.6	**2x**	2451.5	**-45.9**	**0.023**	**0.269**	**0.026**	**0.632**	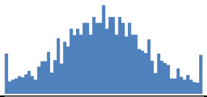
									3x	1463.7	941.8	0.030	-0.167	0.060	0.114	
									4x	1349.6	1056.1	**0.015**	-0.222	0.095	0.083	
R118	*D. sylvatica*	Wilson & Buncher 14 K000728153 (1958)	371016	85.9	1855	177	0.50	2926.9	**2x**	2761.1	**165.7**	**0.013**	**0.476**	**0.032**	**0.781**	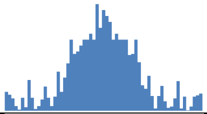
									3x	1232.3	1694.5	0.046	-0.411	0.087	0.274	
									4x	1643.7	1283.1	0.020	-0.076	0.158	0.003	
R119	*D. sylvatica*	Winter & Vahrmeijer 863 K000728149 (1966)	388560	90.0	2836	194	0.84	5282.4	**2x**	5030.4	**251.9**	**0.007**	**0.621**	**0.026**	**0.900**	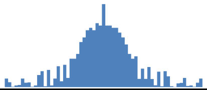
									3x	1916.2	3366.2	0.053	-0.463	0.108	0.236	
									4x	3103.7	2178.6	0.024	0.050	0.193	0.001	
R120	*D. sylvatica*	Strey 8202 K000728150 (1968)	337641	78.2	1444	95	0.37	1695.6	**2x**	1640.0	**55.6**	0.024	**0.271**	**0.036**	**0.488**	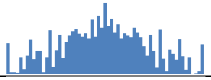
									3x	993.4	702.2	0.030	-0.152	0.070	0.073	
									4x	901.1	794.5	0.016	-0.164	0.112	0.035	
R121	*D. sylvatica*	Balsinhas 3080 K000728155 (1976)	376707	87.2	2435	163	0.65	3669.6	**2x**	3604.5	**65.2**	**0.015**	**0.412**	**0.029**	**0.762**	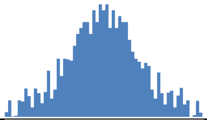
									3x	1681.4	1988.1	**0.038**	-0.270	0.082	0.155	
									4x	1944.8	1724.8	**0.019**	-0.158	0.137	0.022	
R122	*D. sylvatica*	Pole Evans 4841 K001148759 (1954)	401376	92.9	1976	651	0.49	5072.5	**2x**	4332.9	**739.5**	**0.001**	**0.906**	**0.020**	**0.969**	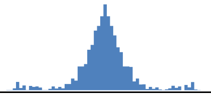
									3x	1339.2	3733.2	0.079	-0.694	0.149	0.268	
									4x	3143.9	1928.6	0.033	0.534	0.262	0.065	
R123	*D. sylvatica*	Pole Evans 4858 K001148760 (1954)	401835	93.1	1600	530	0.40	4035.2	**2x**	3507.9	**527.3**	**0.001**	**0.898**	**0.024**	**0.959**	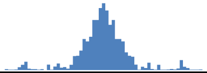
									3x	1087.1	2948.1	0.077	-0.671	0.150	0.252	
									4x	2518.1	1517.1	0.033	0.521	0.261	0.063	
R124	*D. sylvatica*	Mabatha & Nkuna 2555 K000728156 (2009)	405687	93.9	1883	403	0.46	4156.0	**2x**	3539.4	**616.7**	**0.002**	**0.797**	**0.020**	**0.960**	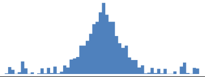
									3x	1249.7	2906.3	0.069	-0.632	0.130	0.285	
									4x	2587.7	1568.3	0.027	0.419	0.233	0.051	
R126	*D. sylvatica*	Norlinch & Weimarck 941 K000728165 (1930)	347154	80.4	2385	110	0.69	2748.5	**2x**	2638.2	**110.2**	**0.024**	**0.245**	**0.026**	**0.593**	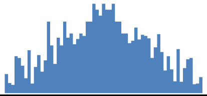
									3x	1678.9	1069.6	**0.026**	-0.087	0.059	0.035	
									4x	1429.9	1318.5	**0.014**	-0.167	0.091	0.054	
R134	*D. sylvatica*	Rogers 12828 K000728188 (1913)	317262	73.5	1637	88	0.52	1948.7	**2x**	1871.8	**76.9**	0.024	**0.260**	**0.031**	**0.530**	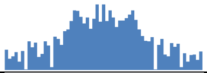
									3x	1119.3	829.3	0.031	-0.202	0.062	0.150	
									4x	1031.2	917.5	**0.015**	-0.190	0.102	0.055	
R135	*D. sylvatica*	Long 921 K000728190 (1933)	397554	92.1	1683	345	0.42	3149.9	**2x**	2993.7	**156.1**	**0.007**	**0.623**	**0.026**	**0.902**	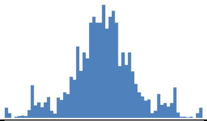
									3x	1145.4	2004.4	0.052	-0.439	0.110	0.211	
									4x	1833.2	1316.6	0.023	0.081	0.193	0.002	
R136	*D. sylvatica*	Wilson & Buchner	397299	92.0	2385	228	0.60	4852.7	**2x**	4692.0	**160.6**	**0.005**	**0.669**	**0.025**	**0.920**	

		136							3x	1642.0	3210.6	0.055	-0.463	0.117	0.208	
		K000728193 (1958)							4x	2805.5	2047.1	0.026	0.053	0.205	0.001	
R139	*D. sylvatica*	Burkill s.n.	384648	89.1	3217	248	0.84	5353.9	**2x**	5162.4	**191.4**	**0.011**	**0.509**	**0.025**	**0.872**	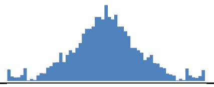
		K000728213							3x	2181.5	3172.3	0.043	-0.342	0.093	0.186	
		(1936)							4x	2950.2	2403.6	0.021	-0.123	0.160	0.009	
R141	*D. sylvatica*	Plessis s.n.	382344	88.5	2052	128	0.54	3394.9	**2x**	3350.5	**44.4**	**0.011**	**0.510**	**0.030**	**0.822**	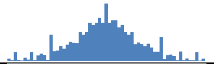
		K000728230							3x	1409.8	1985.1	0.043	-0.303	0.099	0.136	
		(1954)							4x	1867.9	1527.0	**0.022**	-0.110	0.165	0.007	
R142	*D. sylvatica*	Mogg s.n.	384255	89.0	2260	99	0.59	4073.4	**2x**	3880.4	**193.0**	**0.009**	**0.577**	**0.031**	**0.852**	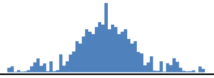
		K000728242							3x	1532.8	2540.5	0.051	-0.448	0.103	0.242	
		(1938)							4x	2332.9	1740.5	0.023	0.024	0.184	0.000	
R143	*D. sylvatica*	Brani 5855	398382	92.3	2240	487	0.56	5212.4	**2x**	4977.5	**234.8**	**0.002**	**0.796**	**0.019**	**0.966**	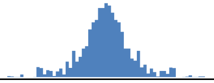
		K001148314							3x	1551.7	3660.7	0.065	-0.550	0.135	0.217	
		(1943)							4x	3090.3	2122.0	0.030	0.235	0.237	0.016	
R144	*D. sylvatica*	Kornas 838	401658	93.0	2272	1348	0.54	5323.8	**2x**	3680.2	**1643.6**	**0.004**	**0.849**	**0.034**	**0.912**	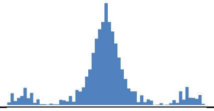
		K001148291							3x	1440.1	3883.6	0.077	-0.694	0.142	0.287	
		(1972)							4x	3443.3	**1880.5**	0.027	0.740	0.243	0.135	
R145	*D. sylvatica*	Luwiika et al. 589	404112	93.6	2399	354	0.59	5949.7	**2x**	5641.3	**308.3**	**0.001**	**0.847**	**0.015**	**0.980**	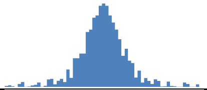
		K001148300							3x	1659.6	4290.1	0.071	-0.626	0.140	0.252	
		(1997)							4x	3550.3	2399.4	0.032	0.302	0.249	0.024	
S49	*D. sylvatica*	K Liv Coll 2011-	404211	93.6	1995	475	0.49	2431.6	**2x**	2324.9	**106.7**	**0.024**	**0.254**	**0.028**	**0.581**	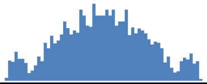
		447 - Cit31							3x	1394.6	1037.0	0.028	-0.125	0.061	0.066	
		(2017)							4x	1234.5	1197.1	**0.016**	-0.238	0.093	0.099	
Code	Species	Sample information (year collected)	bp recov.	%	SNPs	Cov.	P (%)	log*L* free	M	log*L*	*Δ*log*L*	SSR	y-y slope	std.Err	R^2	Histogram
P06	*D. communis*	Korea Hana Park	399882	92.6	3371	524	0.84	5990.8	2x	846.9	5143.9	0.052	0.009	**0.073**	0.000	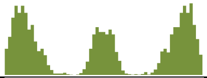
		20150728							3x	1910.3	4080.5	0.042	-0.288	0.100	0.123	
		(2017)							**4x**	5681.2	**309.6**	**0.001**	**0.120**	**0.055**	**0.890**	
R01	*D. communis*	Viruel R01	397809	92.1	3043	637	0.77	5396.1	2x	988.2	4407.9	0.046	0.080	**0.074**	0.019	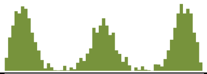
		(2016)							3x	1688.8	3707.3	0.046	-0.390	0.096	0.217	
									**4x**	5274.9	**121.3**	**0.001**	**1.26**	0.044	**0.932**	
R04	*D. communis*	Robinson s.n.	383616	88.8	2165	248	0.56	3174.3	2x	417.3	2757.0	0.058	-0.108	0.064	0.046	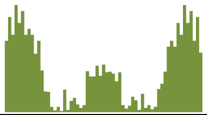
		K001141034							3x	1284.3	1890.0	0.038	-0.236	0.090	0.103	
		(1956)							**4x**	3014.5	159.8	0.002	0.978	0.076	0.732	
R05	*D. communis*	Lewis 44	375690	87.0	3443	215	0.92	3473.5	2x	1721.9	1751.6	0.039	0.032	**0.028**	0.020	

		K001140993							3x	2237.6	1235.9	0.026	-0.150	0.037	0.217	
		(1948)							**4x**	3027.8	**445.7**	**0.002**	**0.459**	0.026	**0.838**	
R06	*D. communis*	Brummitt 5132	377253	87.4	3306	192	0.88	3717.1	2x	1285.2	2431.8	0.045	-0.022	**0.041**	0.005	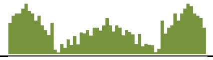
		K001141091							3x	2114.8	1602.2	0.029	-0.178	0.055	0.147	
		(1965)							**4x**	3508.2	**208.9**	**0.001**	**0.637**	**0.044**	**0.774**	
R07	*D. communis*	Bourgeau 656	336636	78.0	1365	62	0.41	1727.2	2X	107.5	1619.7	0.080	-0.294	**0.074**	**0.212**	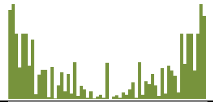
		K000204943							3x	823.8	903.4	0.042	-0.184	0.118	0.015	
		(1847)							**4x**	1628.3	**98.8**	0.015	**0.567**	0.173	**0.153**	
R08	*D. communis*	Edmond 303	335043	77.6	2148	51	0.64	3420.1	2x	-91.6	3511.8	0.090	-0.315	**0.095**	**0.156**	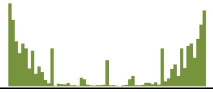
		K001141099							3x	1048.9	2371.2	**0.054**	-0.262	0.147	0.051	
		(1931)							**4x**	3037.1	**382.9**	**0.021**	**0.793**	0.212	**0.191**	
R10	*D. communis*	Atchley 378	368559	85.3	1079	87	0.29	1467.9	2x	33.0	1434.9	0.084	-0.334	**0.076**	**0.247**	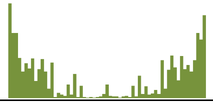
		K001141116							3x	666.9	801.0	0.040	-0.100	0.126	0.010	
		(1931)							**4x**	1375.2	**92.8**	**0.017**	**0.540**	0.185	**0.125**	
R11	*D. communis*	Atchley s.n.	371799	86.1	4029	128	1.08	3874.9	2x	1751.8	2123.0	0.044	-0.029	0.027	0.019	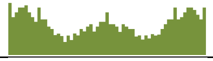
		K001141115							3x	2674.2	1200.6	0.024	-0.100	0.038	0.102	
		(1936)							**4x**	3481.3	**393.5**	**0.003**	**0.399**	0.036	**0.670**	
R12	*D. communis*	s. coll., s.n.	385149	89.2	1411	292	0.37	2404.6	**2x**	2330.7	**73.8**	**0.012**	**0.525**	**0.039**	**0.747**	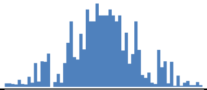
		K001141176							3x	970.3	1434.2	0.046	-0.334	0.106	0.142	
		(1937)							4x	1325.6	1079.0	**0.023**	-0.050	0.179	0.001	
R13	*D. communis*	Kotschy 493	368304	85.3	988	95	0.27	1237.8	2x	127.4	1110.3	0.077	-0.271	**0.072**	**0.193**	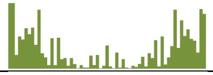
		K001141182							3x	694.4	543.3	0.034	-0.018	0.116	0.000	
		(1836)							**4x**	1182.0	**55.8**	**0.013**	**0.579**	0.165	0.172	
R14	*D. communis*	s. coll. 781	394872	91.4	2230	417	0.57	2266.4	**2x**	2040.7	**225.6**	0.033	**0.113**	**0.026**	**0.235**	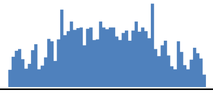
		K001141198							3x	1627.8	638.5	0.022	-0.036	0.044	0.011	
		(1967)							4x	1198.4	1067.9	**0.013**	-0.231	0.062	0.190	
R15	*D. communis*	Hewer 4620	392520	90.9	1466	187	0.37	2712.0	**2x**	2438.8	**273.2**	**0.006**	**0.668**	**0.028**	**0.900**	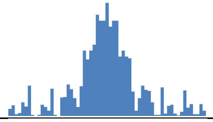
		K001141202							3x	969.2	1742.7	0.057	-0.499	0.116	0.237	
		(1979)							4x	1685.0	1026.9	0.023	0.261	0.205	0.027	
R17	*D. communis*	Davis 5409	326682	75.7	3805	48	1.16	6021.9	2x	2412.0	6029.1	0.086	-0.351	**0.075**	0.268	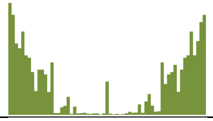
		K001141204							3x	5445.8	3609.9	0.040	-0.088	0.128	0.008	
		(1943)							**4x**	6029.1	**576.0**	**0.014**	**0.734**	0.128	0.228	
R18	*D. communis*	Davis 4353	365751	84.7	1186	100	0.32	2158.9	2x	-49.3	2208.2	0.089	-0.250	**0.107**	0.084	
		K001141208							3x	464.3	1694.5	0.063	-0.389	0.154	0.096	

		(1942)							**4x**	1543.4	**615.4**	**0.025**	**0.770**	0.233	**0.156**
R20	*D. communis*	Al-Kaisi et al.	389250	90.1	871	166	0.22	1278.6	**2x**	1102.7	**175.8**	**0.014**	**0.530**	**0.049**	**0.656**
		45349							3x	554.9	723.7	0.046	-0.288	0.118	0.091
		K001141238 (2004)							4x	815.2	463.4	0.022	0.163	0.191	0.012	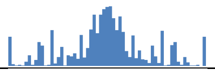
R21	*D. communis*	Lindsay 798	374748	86.8	472	126	0.13	487.5	2x	224.1	263.4	0.053	0.000	**0.074**	0.000
		K001141240							3x	314.7	172.7	0.035	-0.099	0.107	0.014
		(1937)							**4x**	430.1	**57.4**	**0.015**	**0.373**	0.161	0.082	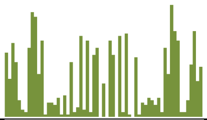
R22	*D. communis*	Atherlon 610	394491	91.4	1929	111	0.49	2542.3	**2x**	2469.7	**72.6**	**0.017**	**0.387**	**0.026**	**0.780**
		K001141245							3x	1287.5	1254.7	0.035	-0.241	0.076	0.142
		(1955)							4x	1419.8	1122.4	**0.017**	-0.104	0.128	0.011	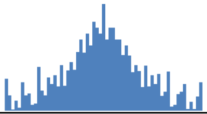
R23	*D. communis*	Fay 1006	375099	86.9	814	87	0.21	1029.8	2x	85.9	943.9	0.076	-0.217	**0.085**	0.098
		K001141256							3x	443.9	585.9	0.047	-0.240	0.127	0.056
		(1978)							**4x**	967.1	**62.7**	**0.017**	**0.641**	0.186	**0.167**	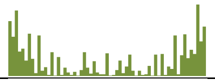
R25	*D. communis*	Trethewy 279	355125	82.2	758	49	0.21	716.2	2x	217.1	499.0	0.065	-0.036	**0.097**	0.002
		K001141260							3x	416.2	299.9	0.047	-0.157	0.139	0.021
		(1934)							**4x**	693.2	**22.9**	**0.022**	**0.473**	0.211	**0.078**	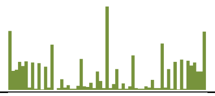
R27	*D. communis*	Christenhurz et al.	394749	91.4	2810	211	0.71	3855.8	2x	831.5	3024.3	0.047	0.011	**0.059**	0.000
		7100							3x	1536.9	2318.9	0.040	-0.351	0.074	0.275
		(2017)							**4x**	3796.2	**59.6**	**0.001**	**0.953**	**0.054**	**0.838**	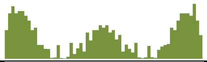
R28	*D. communis*	Medail R28	395697	91.6	3335	207	0.84	4987.3	2x	862.6	4124.6	0.052	-0.034	0.063	0.005
		(2016)							3x	1958.1	3029.2	0.037	-0.253	0.086	0.127
									**4x**	4728.9	**258.3**	**0.001**	**1.02**	**0.055**	**0.851**	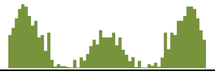
R29	*D. communis*	Viruel R29	395751	91.6	2415	224	0.61	3822.8	2x	521.5	3201.3	0.056	-0.067	**0.068**	0.016
		(2016)							3x	1409.5	2313.3	0.040	-0.274	0.093	0.128
									**4x**	3528.3	**194.5**	**0.002**	**1.080**	**0.066**	**0.817**	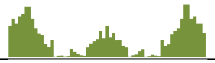
R30	*D. communis*	Medail R30	394335	91.3	2464	127	0.62	3447.3	2x	511.6	2935.7	0.058	-0.104	**0.062**	0.045
		(2016)							3x	1442.3	2005.0	0.038	-0.262	0.086	0.134
									**4x**	3317.3	**130.0**	**0.003**	**0.921**	0.080	**0.687**	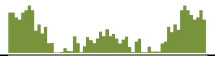
R31	*D. communis*	Viruel R31	399567	92.5	2972	1460	0.74	5648.8	2x	951.1	4697.7	0.048	0.097	0.083	0.022
		(2016)							3x	1637.8	4010.9	0.050	-0.399	0.110	0.181
									**4x**	5518.5	**130.3**	**0.003**	**1.352**	**0.071**	**0.857**	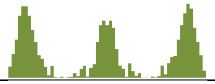
R32	*D. communis*	Davis & Polunin	380712	88.2	2304	148	0.61	3290.6	2x	427.4	2863.1	0.058	-0.098	**0.064**	0.037
		25472							3x	1320.3	1970.3	0.037	-0.231	0.091	0.097
		K001141177 (1956)							**4x**	3079.1	**211.5**	**0.003**	**0.952**	0.083	**0.686**	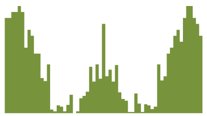

R73	*D. communis*	Porta s.n. K001141014 (1867)	375441	86.9	11900	211	3.17	19437.2	2x	2512.4	16924.8	0.079	-0.376	**0.040**	0.591	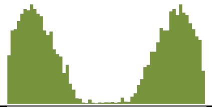
									3x	13667.1	5769.7	0.016	0.323	0.082	0.205	
									**4x**	14113.6	**5323.7**	**0.011**	**0.372**	0.136	**0.112**	
R74	*D. communis*	Hubbard 11137 K001140953 (1943)	369837	85.6	11972	130	3.24	20241.2	2x	-35.7	20276.8	0.086	-0.374	**0.069**	**0.329**	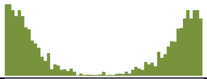
									3x	8106.1	12135.1	0.040	-0.131	0.122	0.019	
									**4x**	**18569.8**	**1671.3**	**0.011**	**0.854**	0.156	**0.334**	
R75	*D. communis*	Steiner 1207 K001141018 (1987)	361206	83.6	11895	108	3.29	16885.2	2x	1694.1	15191.1	0.077	-0.358	**0.041**	**0.556**	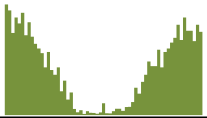
									3x	10643.8	6241.4	0.022	0.146	0.089	0.043	
									**4x**	**14028.4**	**2856.8**	**0.010**	**0.433**	0.130	0.158	
R76	*D. communis*	Jongkind 6014 K001141097 (2004)	383970	88.9	2086	199	0.54	4112.1	2x	-196.3	4308.5	0.101	-0.359	**0.107**	**0.159**	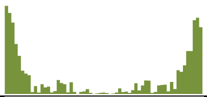
									3x	865.6	3246.4	0.067	-0.409	0.161	0.097	
									**4x**	2943.8	**1168.2**	**0.028**	**0.722**	0.248	**0.125**	
R77	*D. communis*	Harlett 183 K001141102 (1935)	359961	83.4	10921	210	3.03	16415.1	2x	1200.2	15214.9	0.080	-0.367	**0.047**	**0.499**	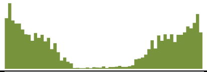
									3x	9399.4	7015.7	0.023	0.152	0.096	0.040	
									**4x**	13234.6	**3180.6**	**0.011**	**0.430**	0.142	**0.133**	
R78	*D. communis*	Atchley 1132 K001141113 (1932)	411807	95.4	9130	194	2.21	9861.9	**2x**	7972.6	**1889.2**	0.025	**0.217**	**0.024**	**0.571**	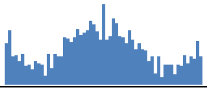
									3x	5718.2	4143.6	0.032	-0.270	0.041	**0.416**	
									4x	5506.4	4355.5	**0.011**	-0.010	0.084	0.000	
R84	*D. communis*	RBGKLiv 1969-19666 (2018)	376572	87.2	5476	90	1.45	8374.5	2x	200.2	8174.3	0.083	-0.369	**0.061**	**0.383**	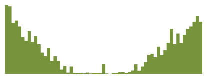
									3x	4012.8	4361.6	0.032	0.013	0.113	0.000	
									**4x**	7420.9	**953.6**	**0.012**	**0.623**	0.156	**0.212**	
R86	*D. communis*	RBGKLiv JLMN182 (2018)	389871	90.3	1976	109	0.51	3694.4	2x	-193.8	3888.2	0.096	-0.338	0.103	**0.154**	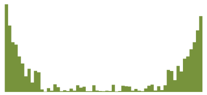
									3x	820.1	2874.2	0.063	-0.399	0.154	**0.101**	
									**4x**	3003.9	**690.5**	**0.023**	**0.871**	0.227	**0.199**	
R94	*D. communis*	Hill 2408 K001141125 (1934)	370596	85.8	6096	119	1.64	10570.0	2x	-187.5	10757.6	0.085	-0.271	**0.093**	**0.124**	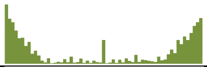
									3x	2719.7	7850.3	0.057	-0.407	0.135	0.132	
									**4x**	8944.6	**1625.4**	**0.017**	**0.912**	0.192	**0.274**	
R95	*D. communis*	Merton 3092 K001141199 (1951)	388509	90.0	803	149	0.21	760.4	2x	273.0	487.3	0.066	-0.149	0.076	0.060	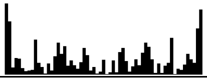
									**3x**	484.2	**276.1**	**0.037**	-0.110	0.114	0.015	
									**4x**	485.1	**275.2**	**0.025**	-0.177	**0.017**	0.016	
R96	*D. communis*	Sahira 37558 K001141223 (2003)	379839	88.0	772	156	0.20	1131.8	2x	64.6	1067.1	0.083	-0.155	**0.112**	0.031	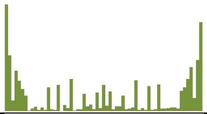
									3x	304.6	827.1	0.065	-0.398	0.158	0.096	
									**4x**	701.1	**430.6**	**0.032**	**0.403**	0.254	0.040	
R129	*D. communis*	Hubbard et al. 82	336156	77.8	1221	128	0.36	1265.8	2x	421.8	843.9	0.057	-0.077	**0.067**	0.022	

		K001141037 (1932)							3x	675.5	590.3	0.040	-0.285	0.091	**0.141**	
									**4x**	895.5	**370.2**	**0.015**	**0.194**	0.151	0.027	
S25	*D. communis*	Viruel S25 (2016)	393120	91.0	1004	426	0.26	1156.8	2x	329.4	827.3	0.062	-0.160	**0.060**	**0.107**	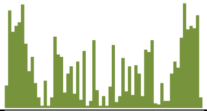
									3x	697.1	459.6	0.035	-0.178	0.089	0.062	
									**4x**	986.5	**170.3**	**0.010**	**0.438**	0.132	**0.155**	
S28	*D. communis*	Verdcourt 4434 K001141070 (1967)	366210	84.8	1107	160	0.30	1394.0	2x	178.6	1215.4	0.075	-0.209	**0.084**	0.093	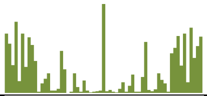
									3x	703.8	690.2	0.044	0.177	0.127	0.031	
									**4x**	1358.4	**35.5**	**0.015**	**0.701**	0.180	**0.204**	
S33	*D. communis*	Alston 1106 K001141124 (1932)	301251	69.8	972	43	0.32	1027.4	2x	187.3	842.1	0.081	-0.196	**0.105**	0.045	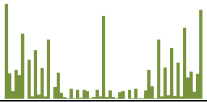
									3x	613.2	416.2	0.045	0.014	**0.156**	0.000	
									**4x**	951.5	**77.9**	**0.028**	**0.422**	0.237	0.050	
S34	*D. communis*	Stainton 7414 K001141123 (1959)	357534	82.8	775	76	0.22	1097.0	2x	44.1	1052.8	0.084	-0.346	**0.070**	**0.288**	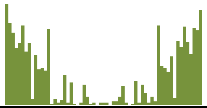
									3x	547.9	549.0	0.036	-0.028	0.122	0.000	
									**4x**	1019.3	**77.6**	**0.015**	**0.606**	0.173	**0.171**	
S35	*D. communis*	Logall 71 K001141126 (1929)	309252	71.6	903	55	0.29	960.7	2x	207.3	753.3	0.071	-0.189	**0.080**	0.086	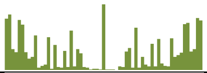
									3x	558.0	402.6	0.042	-0.181	0.119	0.037	
									**4x**	855.3	**105.4**	**0.018**	**0.383**	0.183	0.068	
S42	*D. communis*	Chase et al. 7100 (2017)	398199	92.2	2576	152	0.65	3289.1	2x	732.2	2556.9	0.053	-0.074	**0.055**	0.030	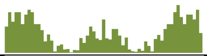
									3x	1652.8	1636.3	0.031	-0.137	0.079	0.048	
									**4x**	3089.0	**200.1**	**0.002**	**0.797**	0.072	**0.670**	
S53	*D. communis*	Galiano et al. 1539/71 SEV12315 (1971)	308154	71.4	714	59	0.23	616.2	2x	269.0	347.2	0.073	-0.129	**0.097**	0.029	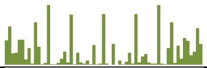
									3x	511.0	105.2	0.041	0.015	0.143	0.000	
									**4x**	553.2	**63.0**	**0.028**	**0.176**	0.222	0.010	
S54	*D. communis*	Cabezudo 1081/76 SEV26339 (1976)	289632	67.1	662	87	0.23	550.3	2x	186.9	363.4	0.071	-0.117	**0.095**	0.025	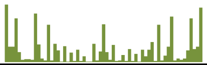
									3x	405.6	144.7	0.040	0.013	0.140	0.000	
									**4x**	523.2	**27.0**	**0.027**	**0.150**	0.271	0.008	
S61	*D. communis*	Raimondo et al. s.n. SEV270115 (1990)	303465	70.3	812	35	0.27	719.9	2x	338.9	380.9	0.073	-0.065	**0.108**	0.006	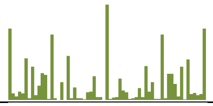
									**3x**	559.6	**160.2**	**0.042**	**0.109**	0.158	0.008	
									**4x**	572.9	**146.9**	**0.034**	0.077	0.247	0.001	
S62	*D. communis*	Talavera et al. 200/03M SEV270667 (2003)	379806	88.0	2702	139	0.71	2502.9	2x	136.7	1196.1	0.045	-0.048	**0.024**	0.061	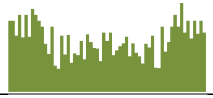
									3x	1925.9	576.9	0.022	-0.045	0.036	0.025	
									**4x**	2101.5	**401.4**	**0.005**	**0.260**	0.046	**0.347**	
S63	*D. communis*	Pina & Raus s.n. SEV280053	383373	88.8	1452	112	0.38	1945.9	2x	288.2	1657.7	0.064	-0.128	**0.074**	0.047	
									3x	903.8	1042.0	0.039	-0.183	0.108	0.046	

									**4x**	1911.6	**34.3**	**0.007**	**0.910**	0.125	**0.469**	
S64	*D. communis*	Ortiz & Salgueiro	397656	92.1	2165	396	0.54	2925.2	2x	833.5	2091.6	0.042	0.056	**0.055**	0.017	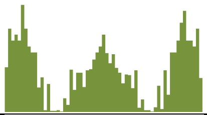
		285/15 SEV286153							3x	1221.3	1703.9	0.040	-0.372	0.064	0.358	
		(2015)							**4x**	2845.8	**79.4**	**0.001**	**0.877**	**0.053**	**0.819**	
S67	*D. communis*	Davis D 38553	356871	82.6	1064	311	0.30	1891.9	**2x**	1746.0	**145.9**	**0.011**	**0.561**	**0.040**	**0.764**	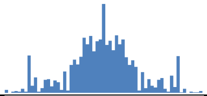
		K001141186							3x	724.2	1167.7	0.050	-0.406	0.109	0.189	
		(1962)							4x	1059.0	832.9	0.022	0.119	0.188	0.006	
S68	*D. communis*	Rico 2255	390813	90.5	1382	159	0.35	2136.6	**2x**	1727.4	**409.2**	**0.012**	**0.514**	**0.034**	**0.789**	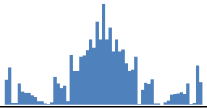
		K000764199							3x	852.0	1284.6	0.051	-0.510	0.087	0.365	
		(2007)							4x	1250.2	886.4	0.018	0.175	0.169	0.017	
S71	*D. communis*	Barkley et al. 139	374037	86.6	808	220	0.22	895.3	2x	658.9	236.4	0.030	**0.231**	**0.054**	**0.235**	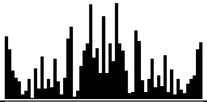
		K001141234							**3x**	489.2	**412.0**	0.037	-0.248	0.084	**0.127**	
		(1963)							**4x**	499.2	**396.0**	**0.017**	-0.037	0.140	0.001	
S73	*D. communis*	Hewer H 4028	388638	90.0	2016	320	0.52	2958.7	2x	360.7	2598.0	0.060	-0.112	**0.068**	0.042	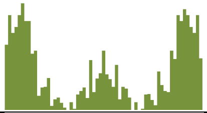
		K001141242							3x	1164.6	1794.1	0.041	-0.280	0.095	0.126	
		(1977)							**4x**	2881.8	**76.9**	**0.003**	**1.032**	0.086	**0.709**	
S76	*D. communis*	Bramwell et al. 92	365781	84.7	1424	335	0.39	2192.8	2x	167.4	2025.4	0.071	-0.214	**0.071**	0.130	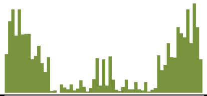
		K001141180							3x	920.3	1272.5	0.038	-0.156	0.110	0.032	
		(1972)							**4x**	2076.0	**116.8**	**0.006**	**0.966**	0.121	**0.518**	
S78	*D. communis*	Helbaek 1902	369435	85.6	525	213	0.14	602.7	**2x**	421.6	**181.1**	0.034	0.279	**0.074**	**0.190**	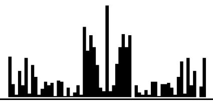
		K001141216							3x	307.8	294.9	0.049	-0.376	0.110	**0.163**	
		(1905)							**4x**	430.8	**171.9**	**0.019**	**0.339**	0.183	0.054	
S79	*D. communis*	Medail S79	399651	92.5	3010	320	0.75	4715.8	2x	815.4	3900.4	0.053	-0.033	**0.067**	0.004	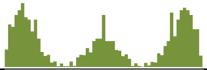
		(2016)							3x	1829.6	2886.2	0.039	-0.251	0.093	0.110	
									**4x**	4437.5	**278.3**	**0.001**	**1.08**	0.059	**0.847**	
S80	*D. communis*	Viruel S80	380271	88.1	764	91	0.20	804.9	2x	229.4	575.5	0.054	-0.020	**0.074**	0.001	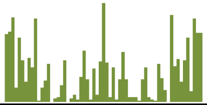
		(2016)							3x	420.0	384.8	0.041	-0.244	0.104	0.085	
									**4x**	784.8	**20.1**	**0.011**	**0.609**	0.149	**0.219**	

Code	Species	Sample information (year collected)	bp recov.	%	SNPs	Cov.	P (%)	log*L* free	M	log*L*	Δlog*L*	SSR	y-y slope	std.Err	R^2	Histogram
Y01	*D. alata*	Lau 28339	146163	95.0	1700	78	1.16	1708.3	**2x**	1600.1	**108.2**	0.029	**0.202**	**0.042**	**0.273**	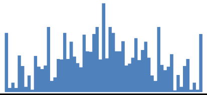
		K001141973							3x	1142.9	565.4	0.028	-0.084	0.072	0.022	
		(1936)							4x	968.1	740.2	**0.016**	-0.183	0.111	0.043	
Y02	*D. alata*	Powell s.n.	93720	60.9	2388	135	2.55	2518.8	**2x**	2091.1	**427.7**	0.027	**0.202**	**0.027**	**0.478**	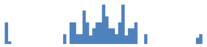
		K001144448							3x	1502.1	1016.7	0.031	-0.242	0.045	**0.323**	

		(1980)							4x	1404.4	1114.4	**0.012**	-0.089	0.085	0.018	
Y03	*D. alata*	Smith 4709	151842	98.7	4220	151	2.78	4197.7	2x	1822.8	2374.8	0.043	-0.015	**0.029**	0.004	
		K001145277							3x	2743.8	1453.8	0.025	-0.130	0.040	0.150	
		(1947)							**4x**	3814.1	**383.5**	**0.002**	**0.464**	0.031	**0.790**	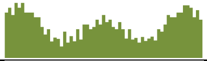
Y04	*D. alata*	Jones 170	151035	98.2	1145	287	0.76	1601.9	2x	224.5	1377.4	0.076	-0.273	**0.069**	**0.206**	
		K000809763							3x	970.1	631.7	0.026	0.178	0.111	0.041	
		(2002)							**4x**	1291.8	**310.0**	**0.014**	**0.492**	0.165	**0.130**	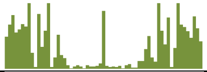
Y05	*D. alata*	Henty 41956	149295	97.1	1859	137	1.25	3252.9	**2x**	2741.3	**511.5**	**0.019**	**0.360**	**0.036**	**0.617**	
		L.1471533							3x	1506.8	1746.0	0.038	-0.287	0.078	0.185	
		(1969)							4x	1681.8	1571.0	**0.018**	-0.143	0.134	0.019	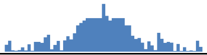
Y06	*D. alata*	Sulit 22491	120513	78.4	855	66	0.71	796.2	2x	136.7	659.5	0.071	-0.189	0.080	0.086	
		K001144846							3x	617.8	178.4	**0.030**	0.132	0.121	0.019	
		(1954)							**4x**	923.9	**-127.6**	**0.020**	**0.256**	0.187	0.030	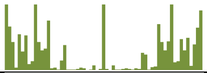
Y07	*D. alata*	Du et al. HNK 2240	150366	97.8	1902	127	1.26	2781.5	**2x**	2012.9	**768.5**	**0.014**	**0.459**	**0.032**	**0.771**	
		K001144044							3x	1124.5	1656.9	0.048	-0.490	0.076	0.414	
		(2008)							4x	1612.3	1169.1	**0.014**	0.273	0.150	0.053	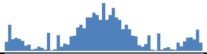
Y09	*D. alata*	Russell 143	149175	97.0	1104	123	0.74	1157.9	2x	747.8	410.1	0.032	**0.200**	**0.054**	0.184	
		K001142743							3x	611.2	546.7	0.043	-0.414	**0.070**	**0.371**	
		(1983)							**4x**	806.8	**351.1**	**0.013**	**0.193**	0.135	0.033	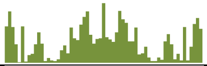
R89	*D. alata*	K Liv. Coll. cit20	371055	85.9	6163	135	1.66	11708.2	2x	-314.6	12022.9	0.092	-0.396	**0.077**	**0.305**	
		(2017)							3x	3944.8	7763.4	0.044	-0.142	0.135	0.019	
									**4x**	10124.8	**1583.4**	**0.014**	**0.902**	0.175	**0.308**	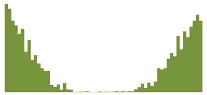
T38	*D. alata*	Christenhusz	408420	94.6	3374	90	0.83	4031.0	2x	1301.8	2729.1	0.068	-0.290	0.024	**0.702**	
		T38							**3x**	3694.1	**336.9**	**0.012**	**0.317**	0.050	**0.397**	
		(2019)							4x	2770.8	1260.2	**0.012**	-0.006	**0.102**	0.007	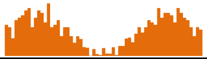
