# Supplementary material for: A Target Capture-Based Method to Estimate Ploidy From Herbarium Specimens
Source: Front Plant Sci. 2019 Jul 24;10:937. doi: 10.3389/fpls.2019.00937 (PMC6667659; doi:10.3389/fpls.2019.00937)

Supplementary Table 4. Estimation of the average and median allelic ratios, the percentage of SNPs with an allelic ratio <2 (% <2) together with boxplots, density plots, allelic ratio and coverage per SNP and boxplot per gene of the allelic ratios of the samples studied; respectively. Red lines represent an allelic ratio of two. Blue lines in allelic ratio per gene graphic represent the median values.

	Boxplot	Density plot	Allelic ratio per SNP	Allelic ratio per gene
Dioscorea sylvatica				
Sample R107<br>Mean 1.29<br>Median 1.13<br>% <2 91.1	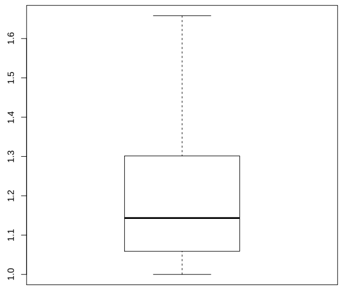	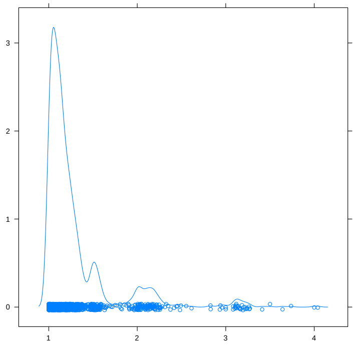	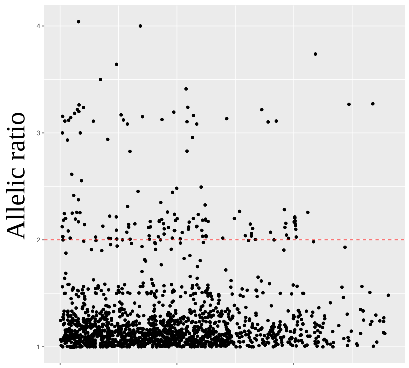	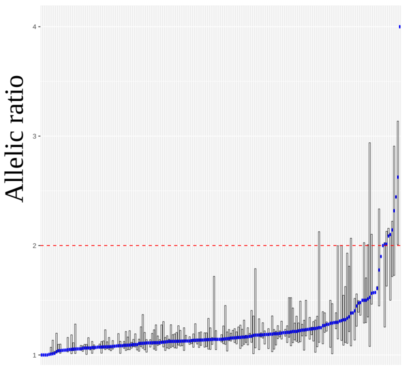
	Allelic ratio	Allelic ratio	Coverage	Genes
Sample R122<br>Mean 1.30<br>Median 1.15<br>% <2 93.1	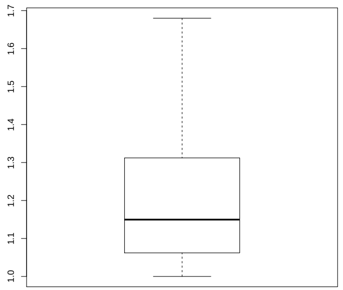	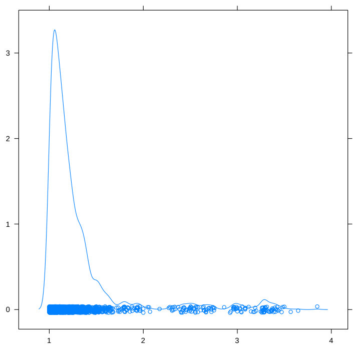	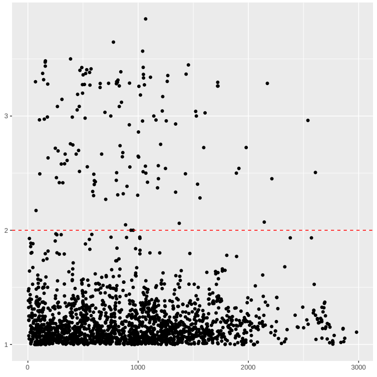	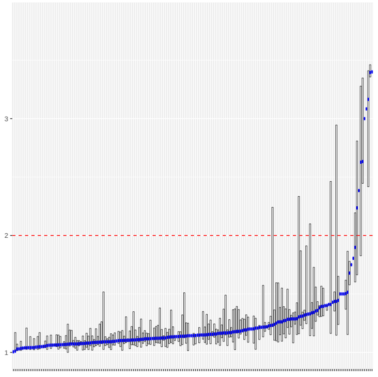

Sample	R123
Mean	1.31
Median	1.15
% <2	92.4

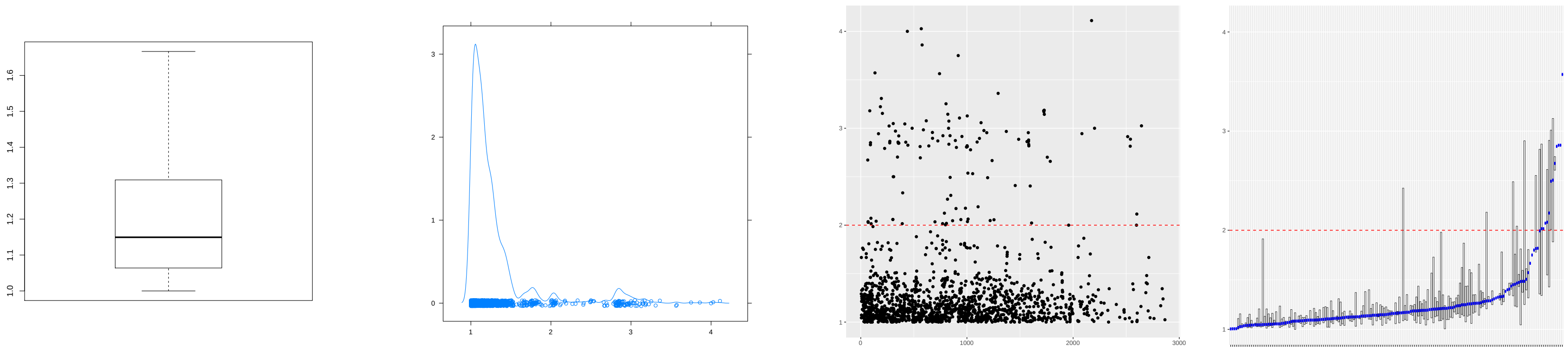

Sample	R144
Mean	1.46
Median	1.15
% <2	84.8

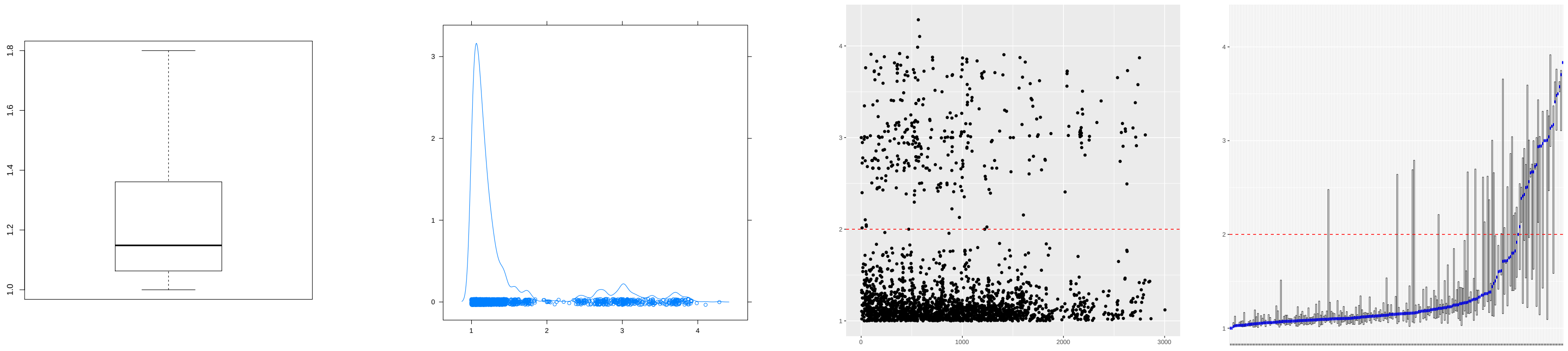

Sample	R145
Mean	1.28
Median	1.17
% <2	95.5

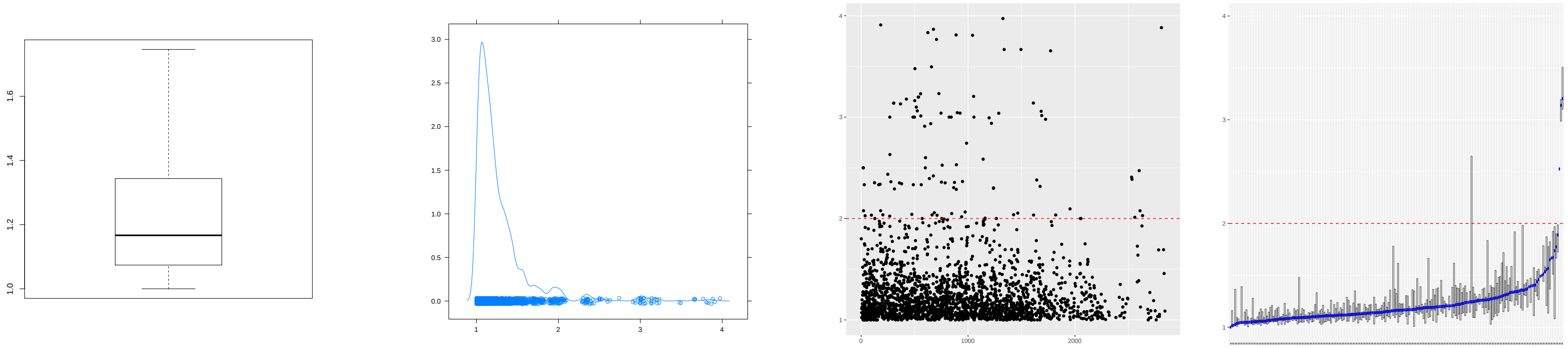

Sample	R124
Mean	1.39
Median	1.18
% <2	89.64

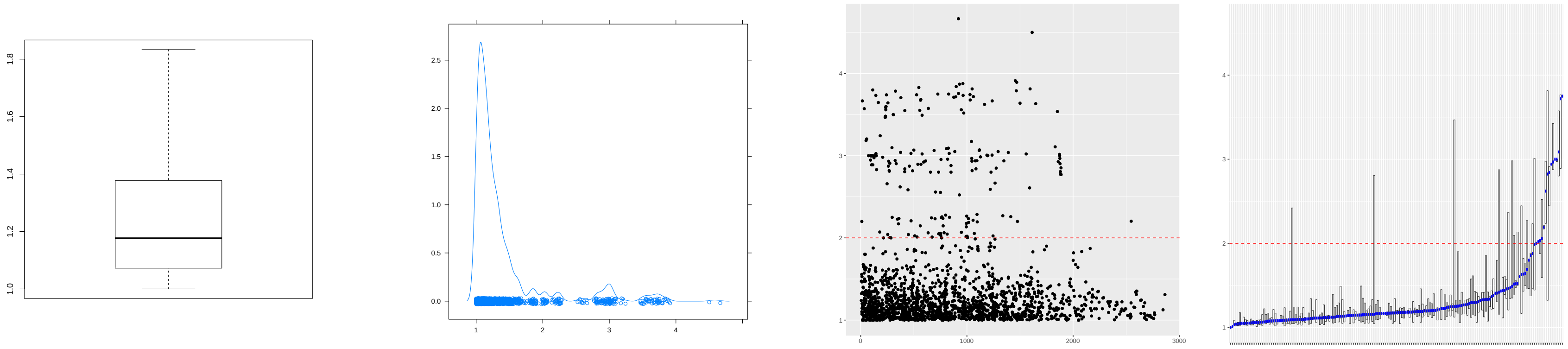

Sample	R143
Mean	1.31
Median	1.18
% <2	92.7

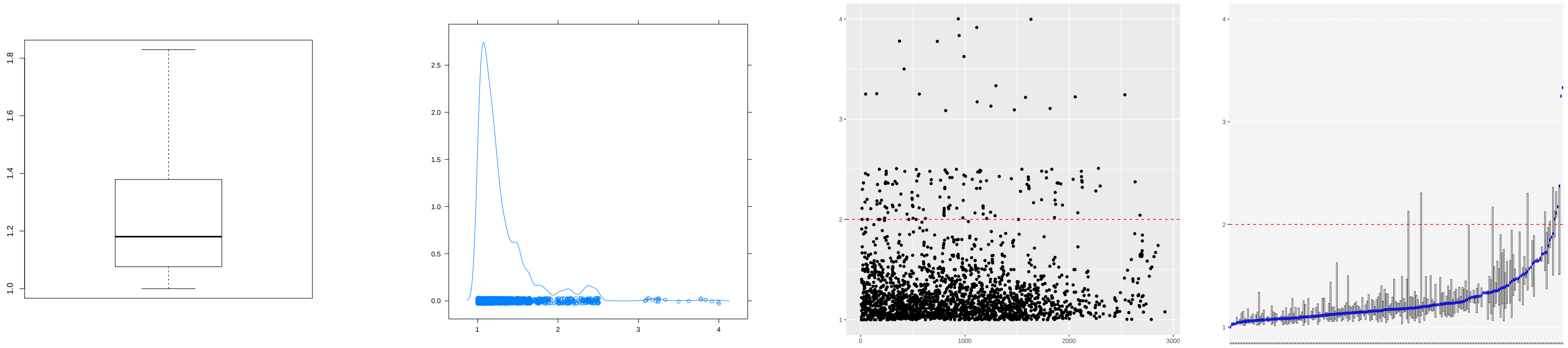

Sample	R112
Mean	1.39
Median	1.21
% <2	90.1

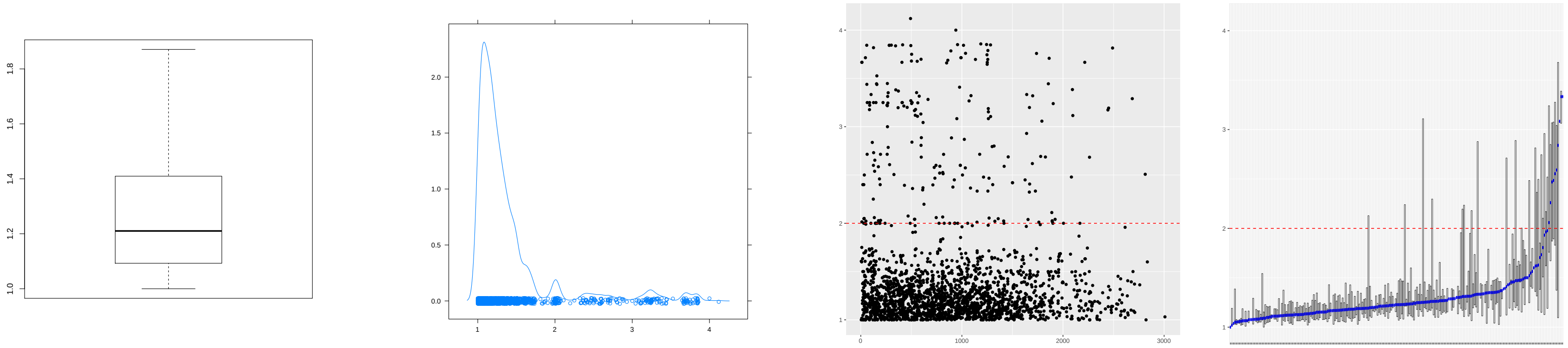

Sample	R104
Mean	1.37
Median	1.19
% <2	91.2

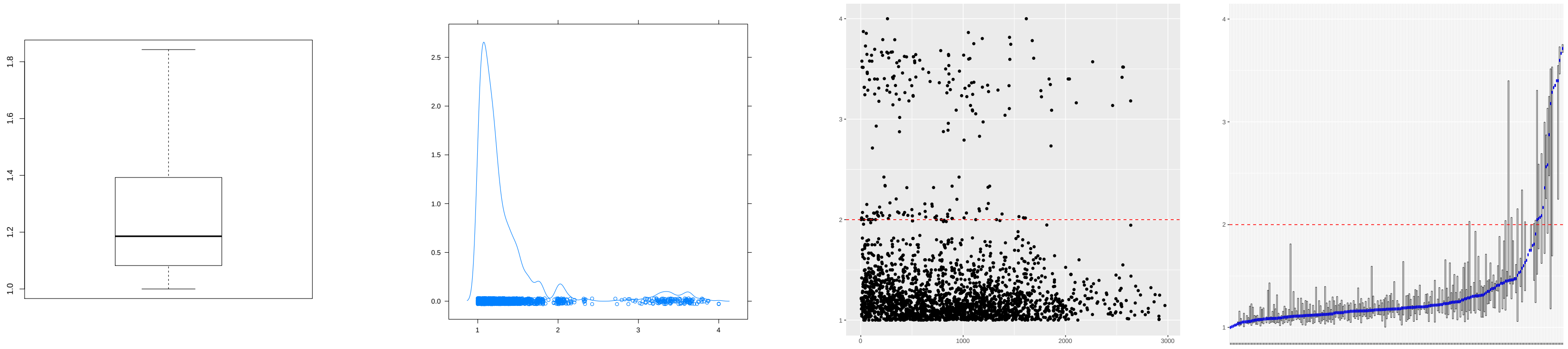

Sample	Z59
Mean	1.36
Median	1.21
% <2	91.7

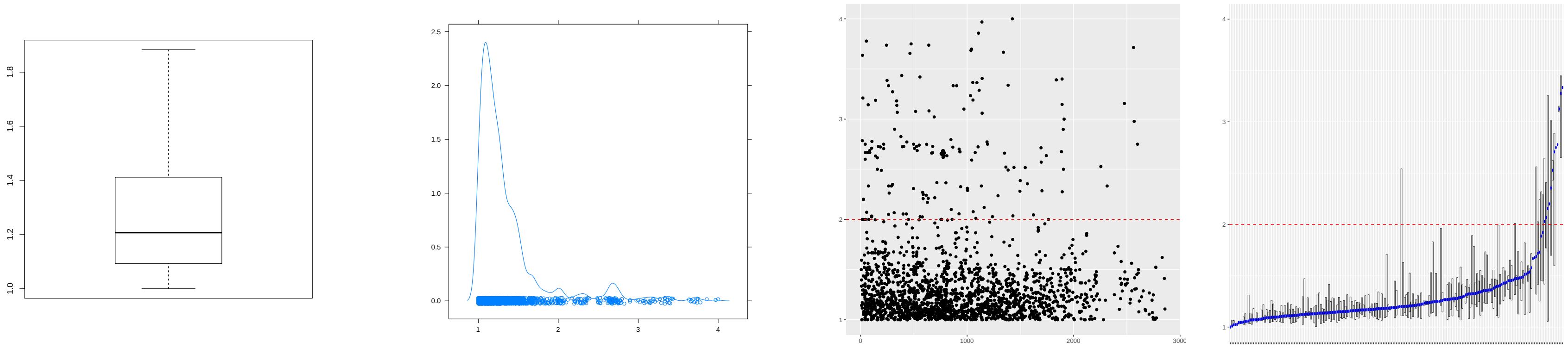

Sample	R111
Mean	1.42
Median	1.23
% <2	87.3

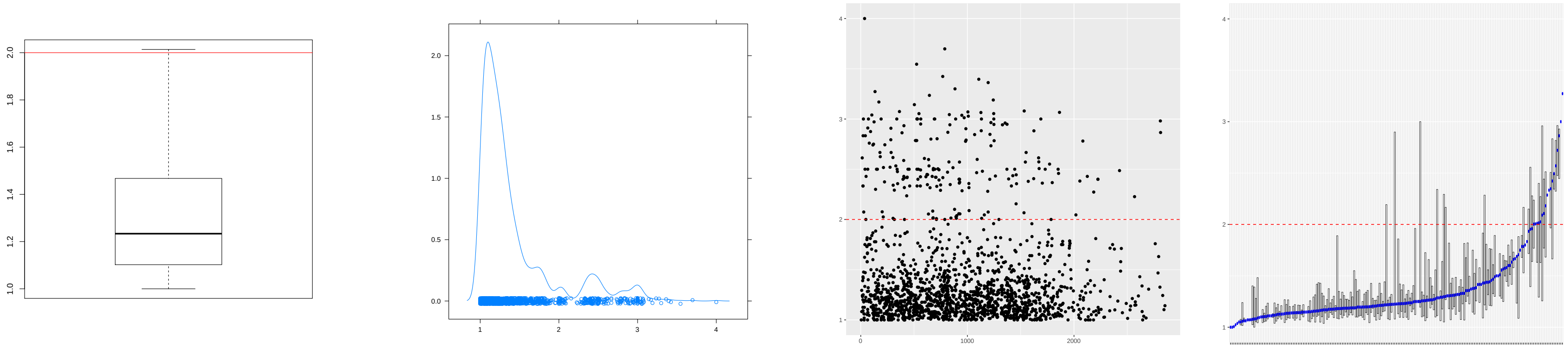

Sample	R136
Mean	1.38
Median	1.22
% <2	90.9

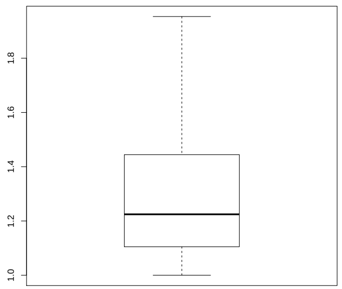
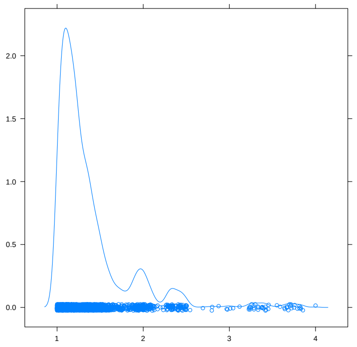
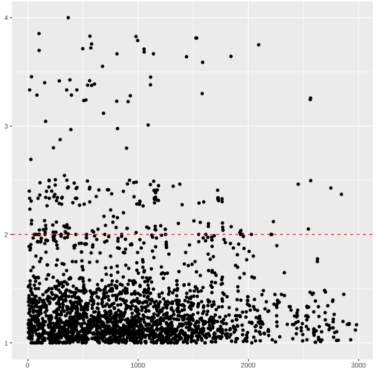
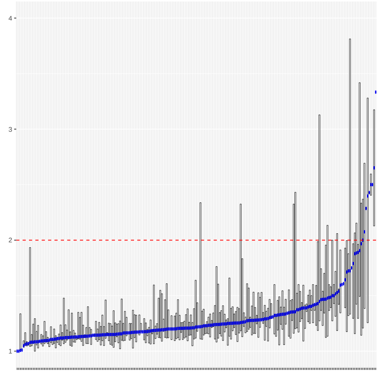

Sample	R106
Mean	1.47
Median	1.24
% <2	85.5

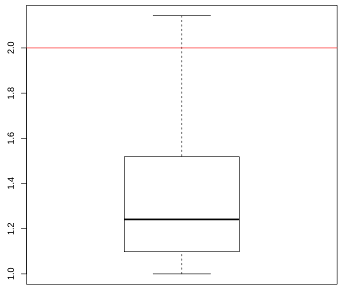
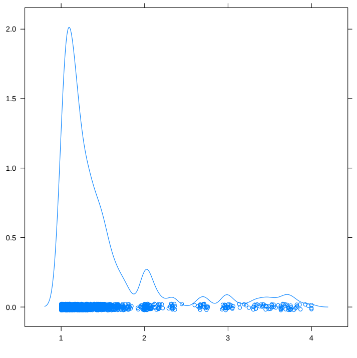
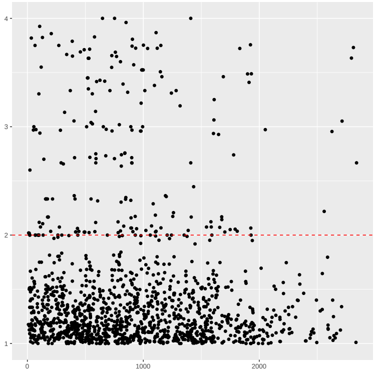
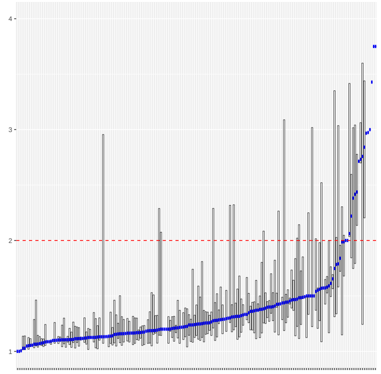

Sample	R135
Mean	1.43
Median	1.24
% <2	84.5

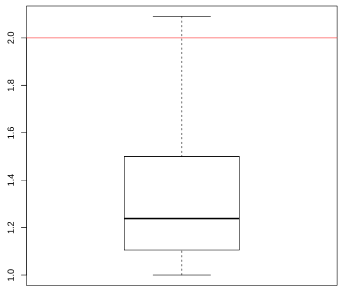
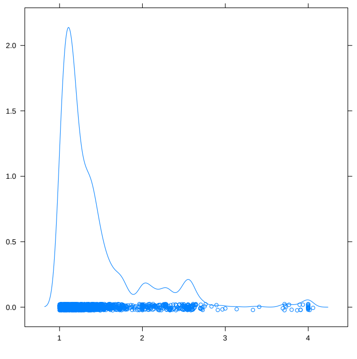
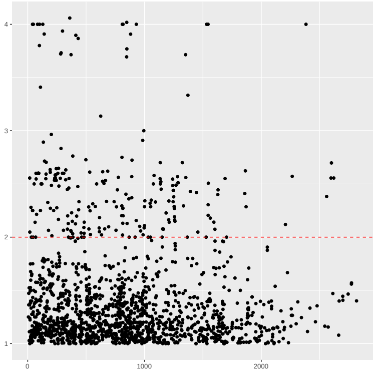
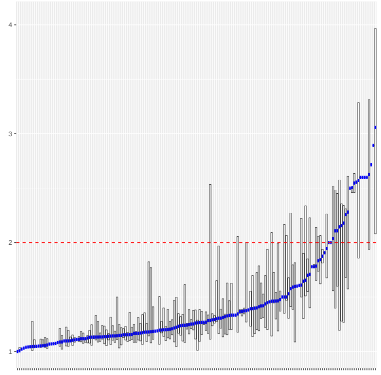

Sample	R142
Mean	1.45
Median	1.27
% <2	86.4

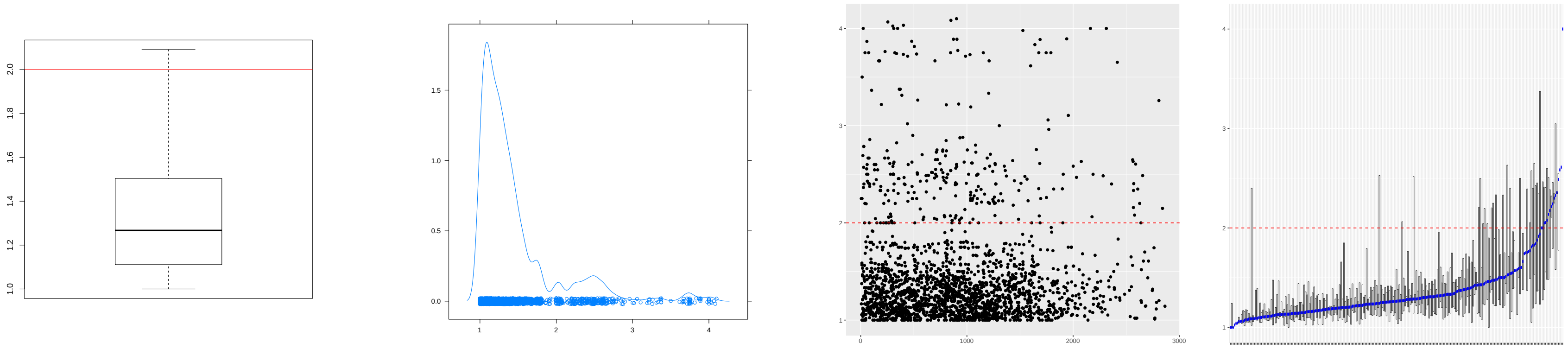

Sample	R139
Mean	1.49
Median	1.9
% <2	87.8

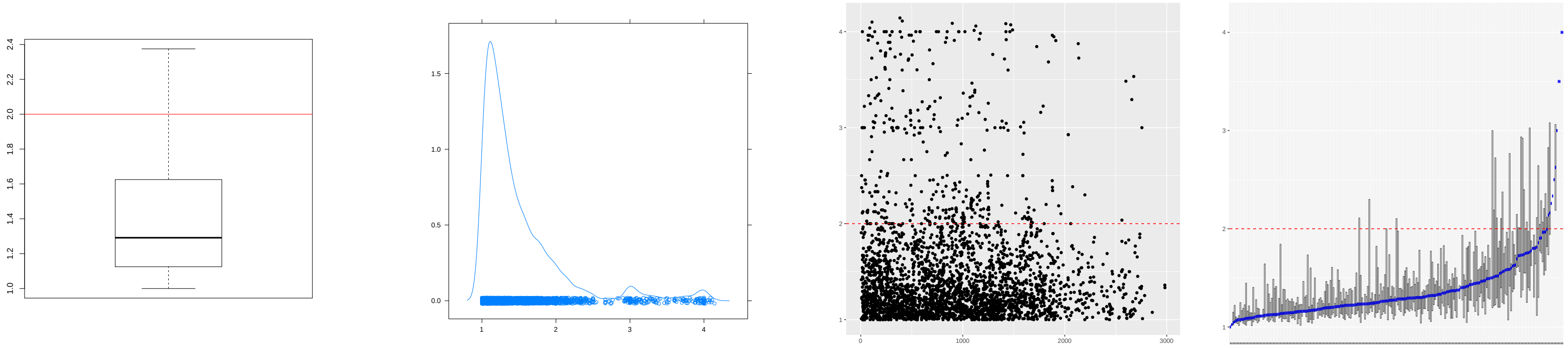

Sample	R141
Mean	1.48
Median	1.29
% <2	85.6

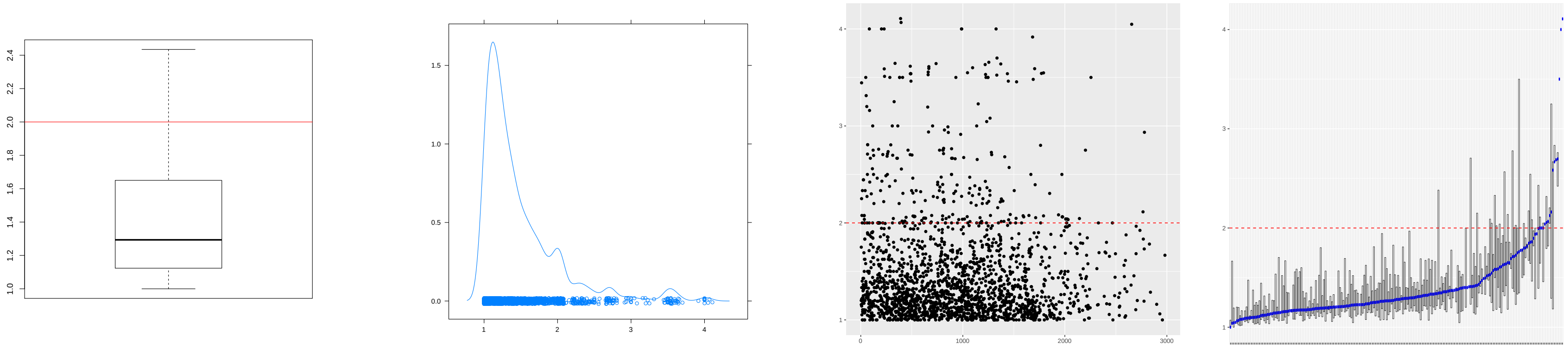

Sample	R114
Mean	1.58
Median	1.32
% <2	81.8

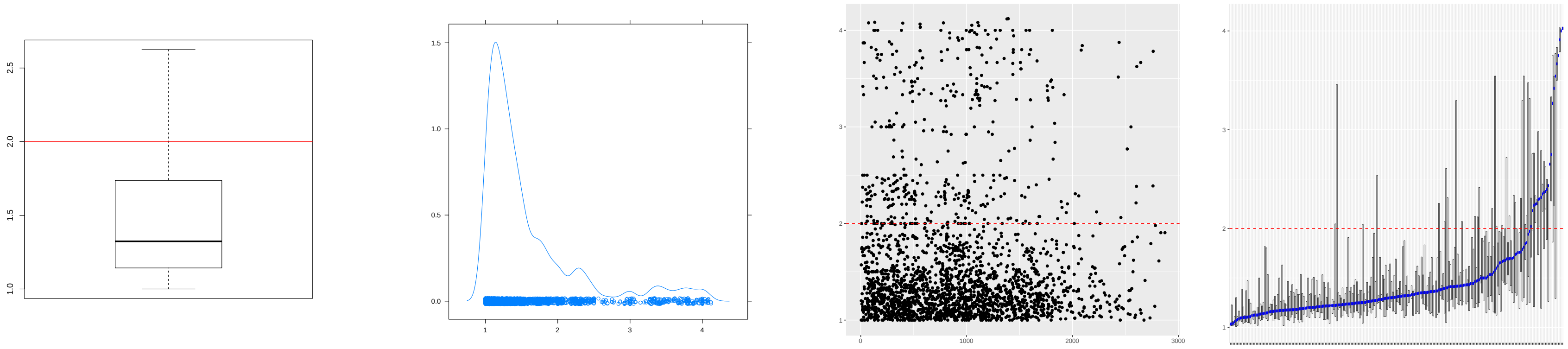

Sample	R118
Mean	1.54
Median	1.32
% <2	84.8

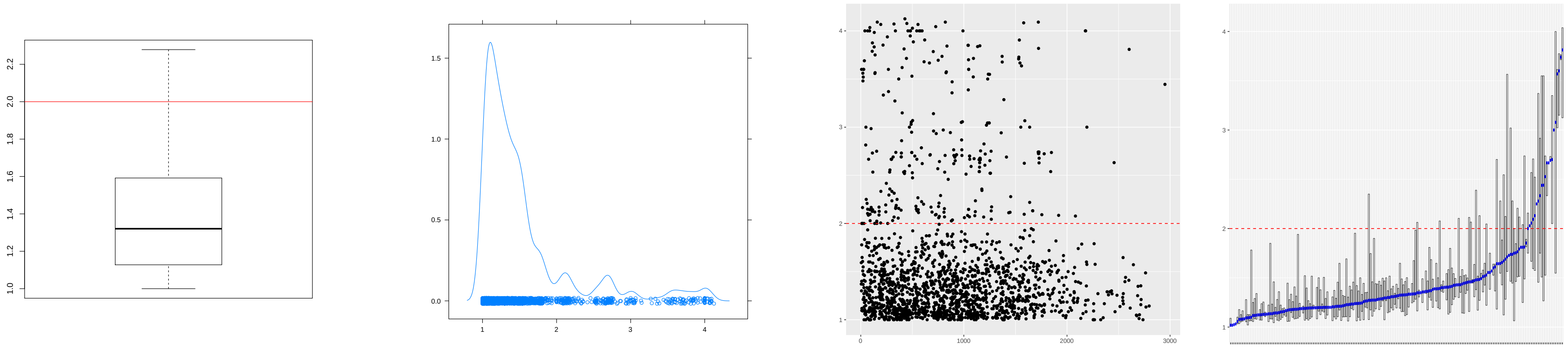

Sample	R121
Mean	1.54
Median	1.34
% <2	83.0

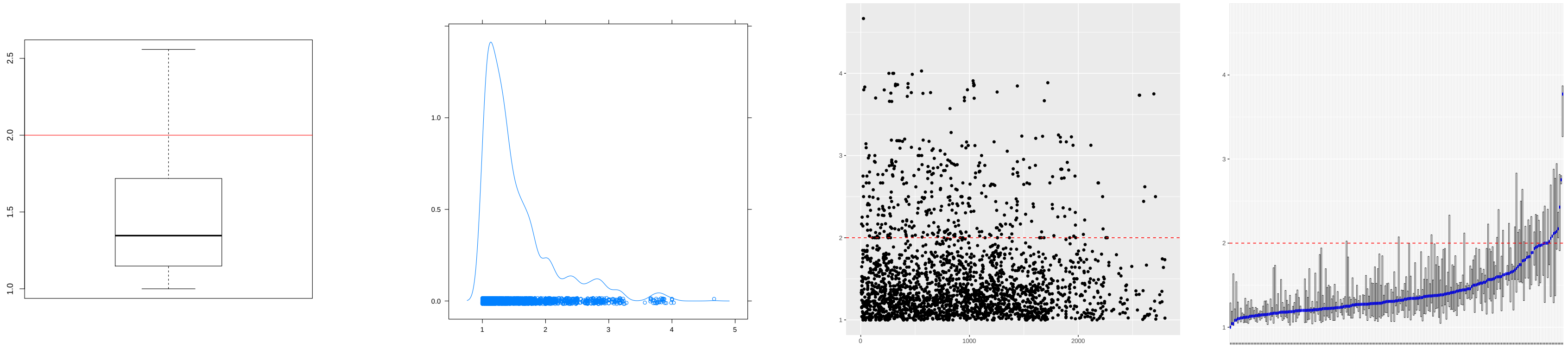

Sample	R134
Mean	1.71
Median	1.45
% <2	74.2

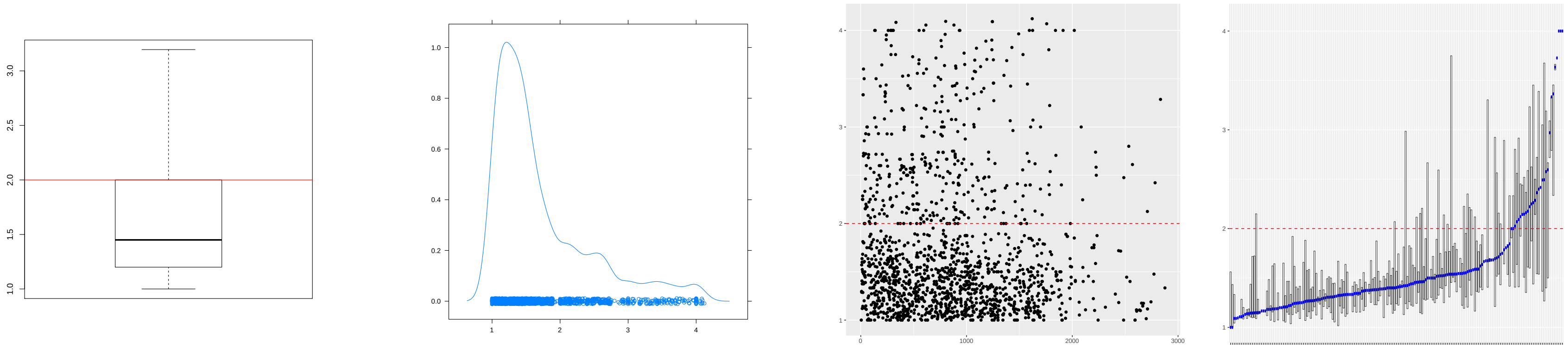

Sample	R117
Mean	1.72
Median	1.46
% <2	74.9

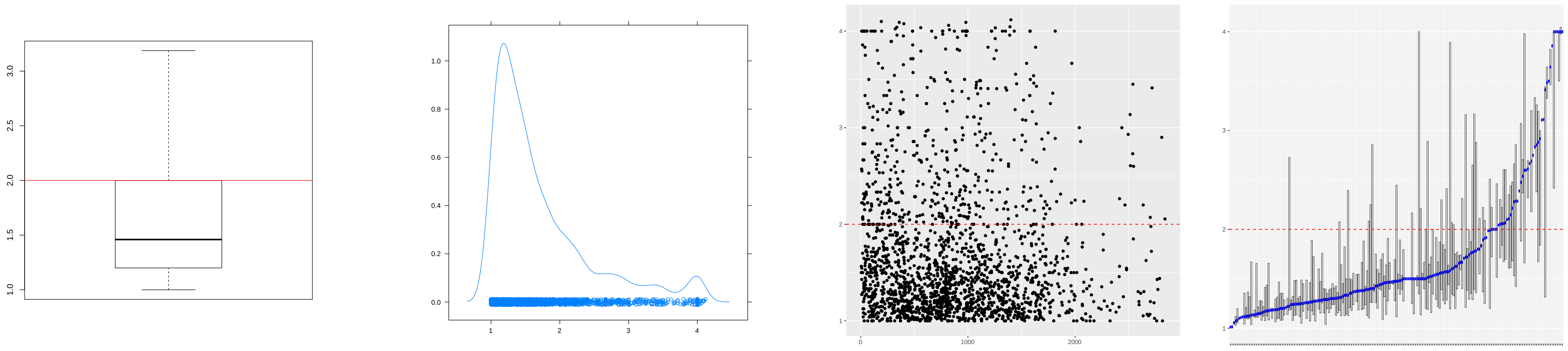

Sample	R120
Mean	1.71
Median	1.47
% <2	73.1

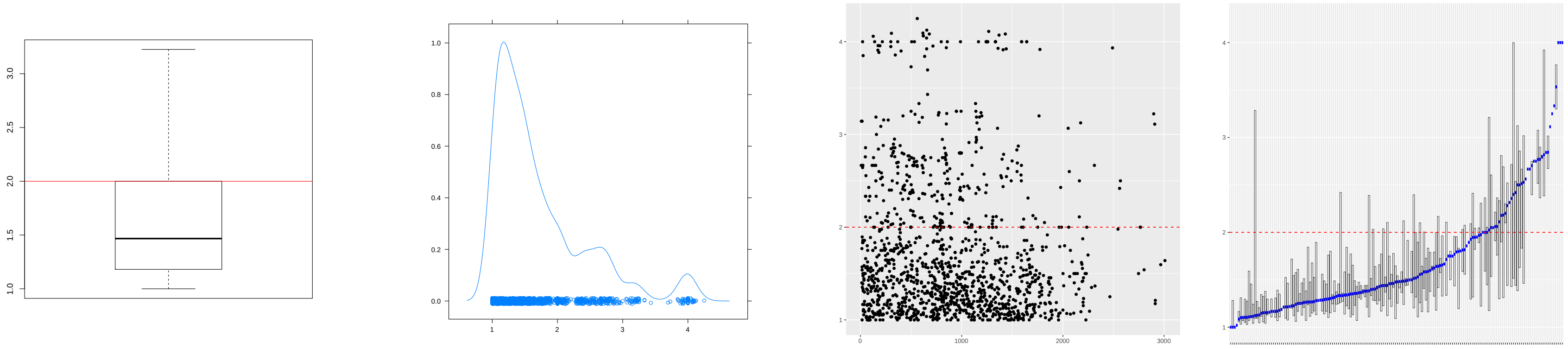

Sample	S49
Mean	1.70
Median	1.48
% <2	77.04

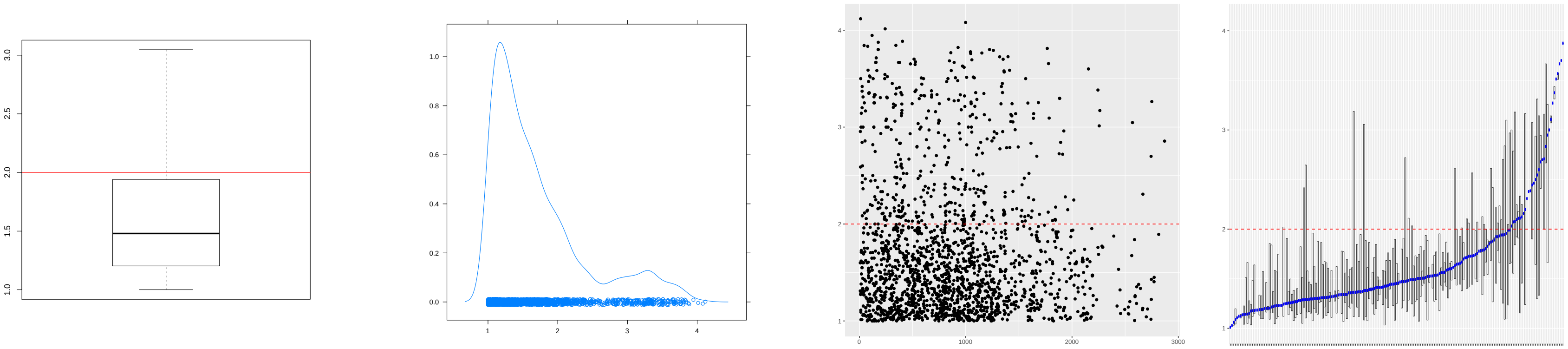

Sample	R126
Mean	1.73
Median	1.52
% <2	72.6

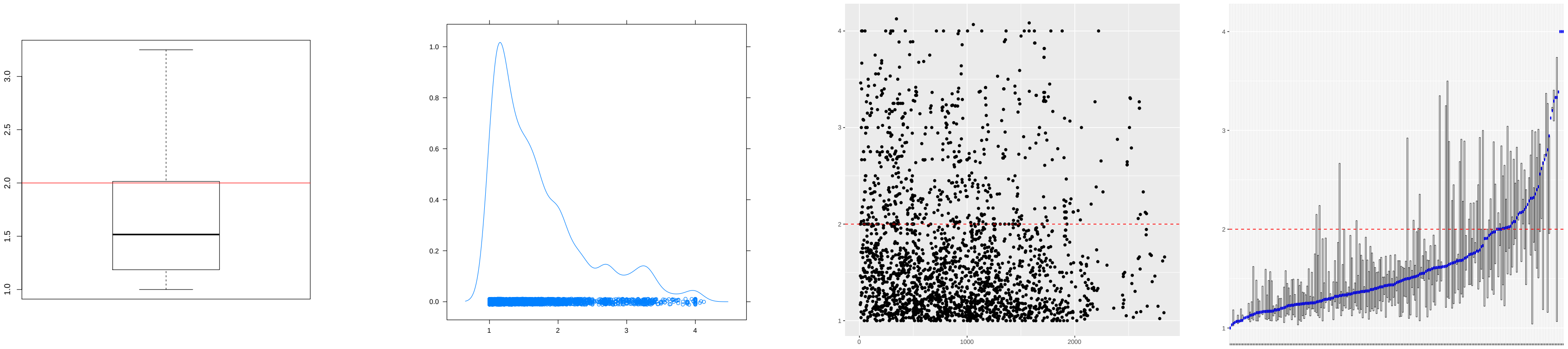

Sample	R103
Mean	2.22
Median	1.72
% <2	55.6

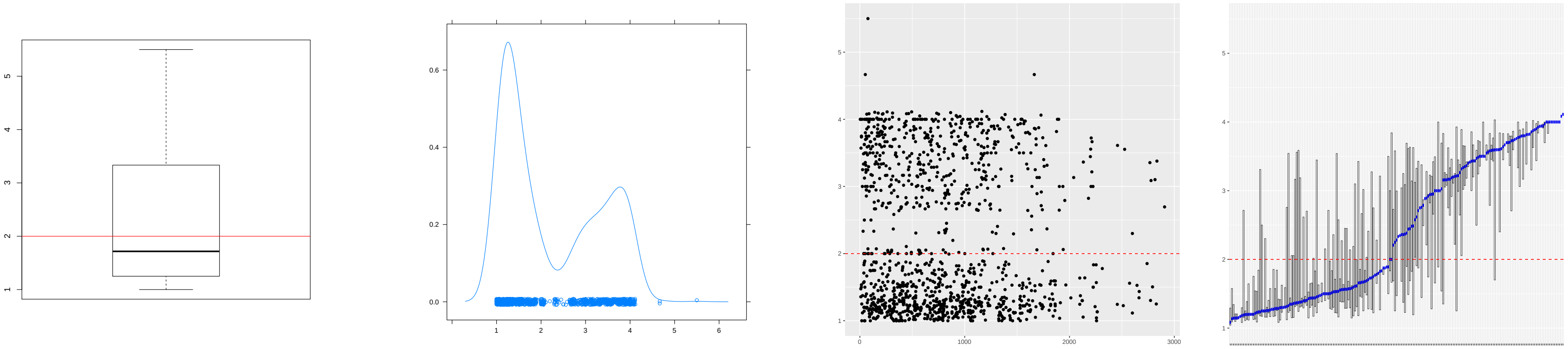

Dioscorea communis

Sample	R15
Mean	1.46
Median	1.22
% <2	85.3

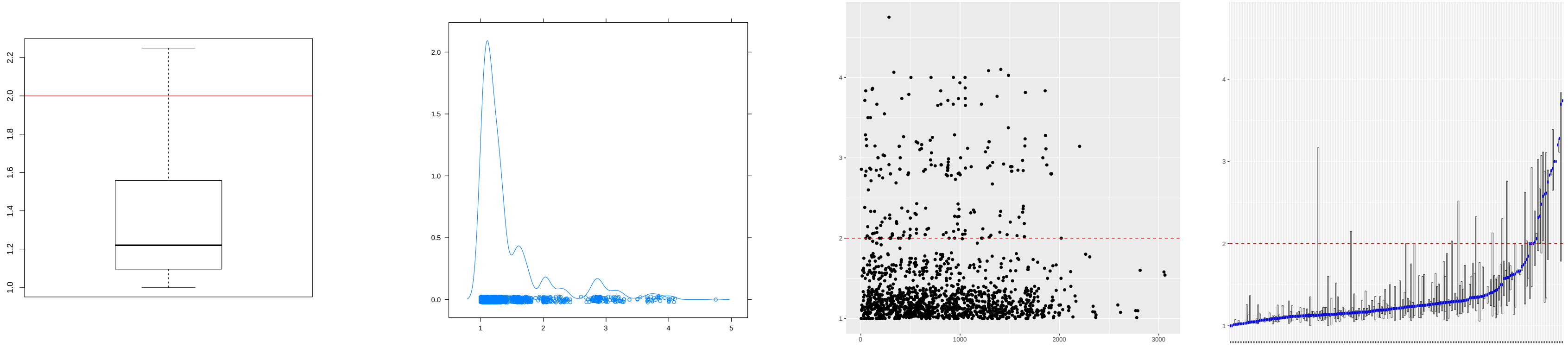

Sample	R20
Mean	1.63
Median	1.25
% <2	74.3

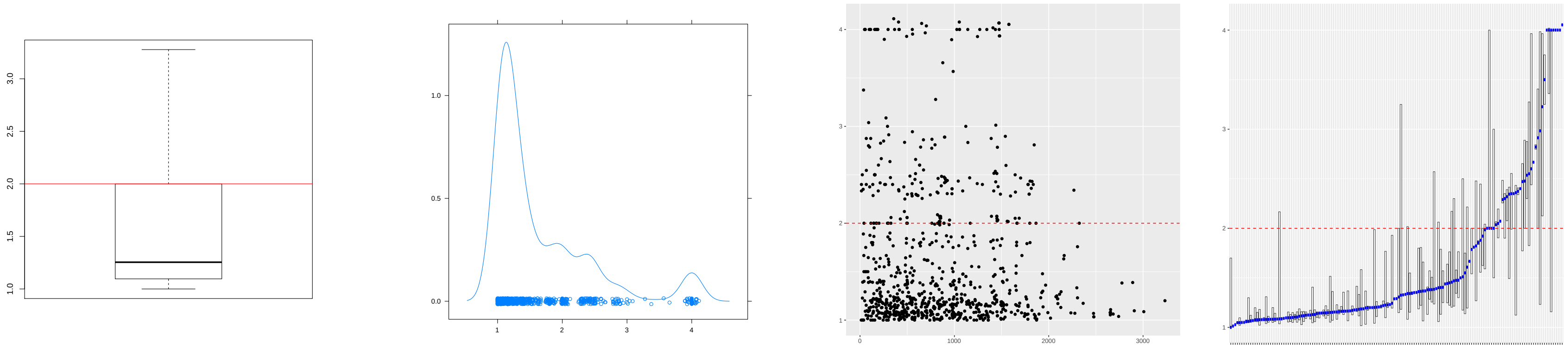

Sample	S67
Mean	1.47
Median	1.27
% <2	83.7

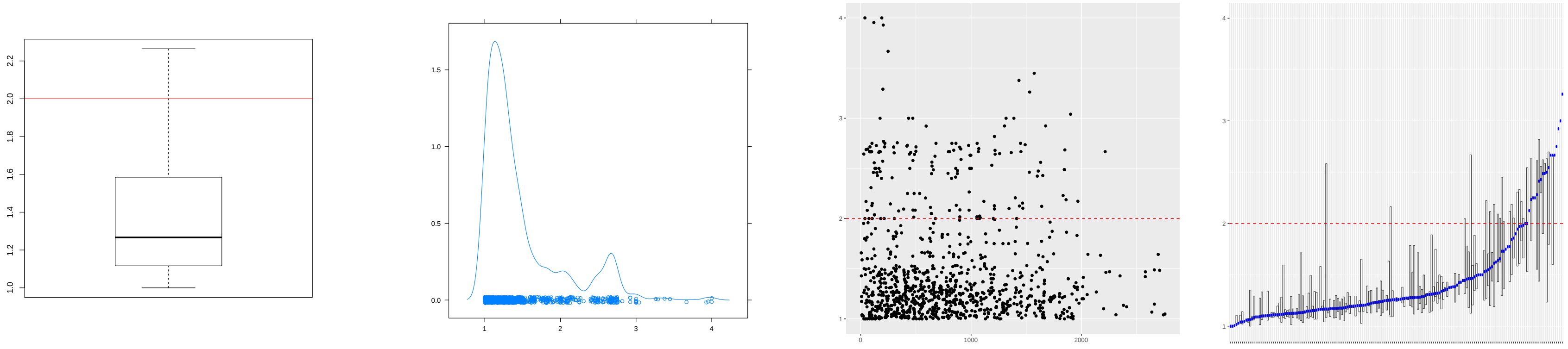

Sample	R12
Mean	1.47
Median	1.27
% <2	82.4

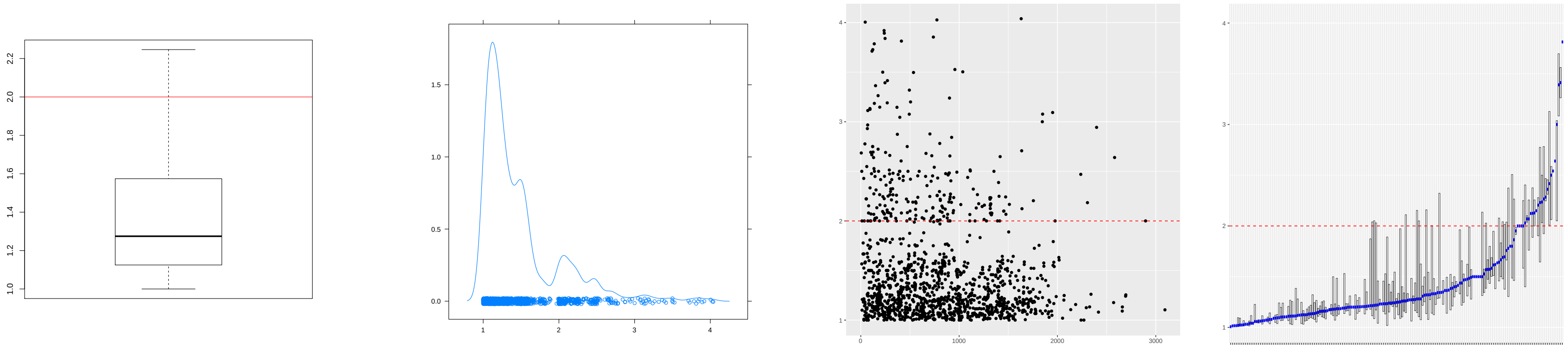

Sample	S68
Mean	1.65
Median	1.29
% <2	81.7

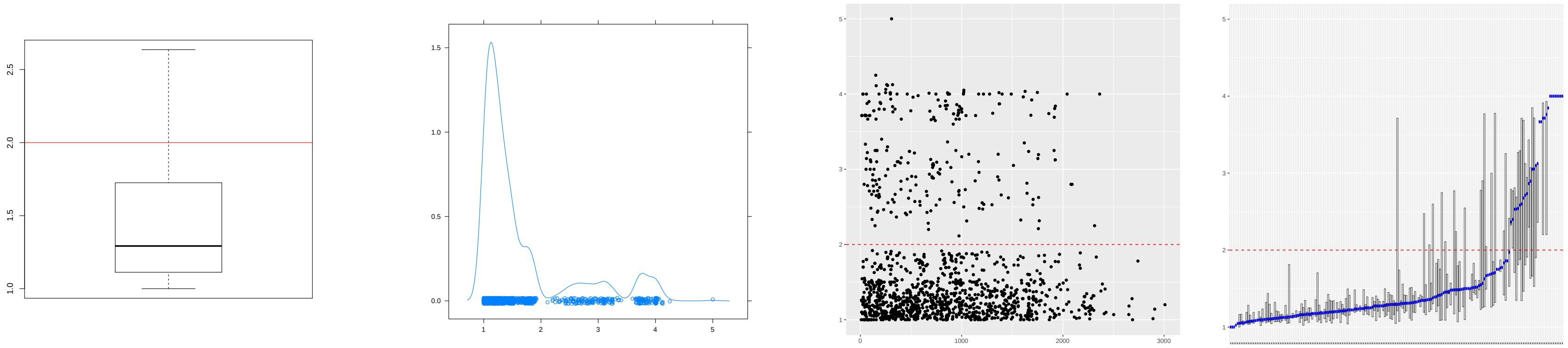

Sample	S78
Mean	1.94
Median	1.34
% <2	61.7

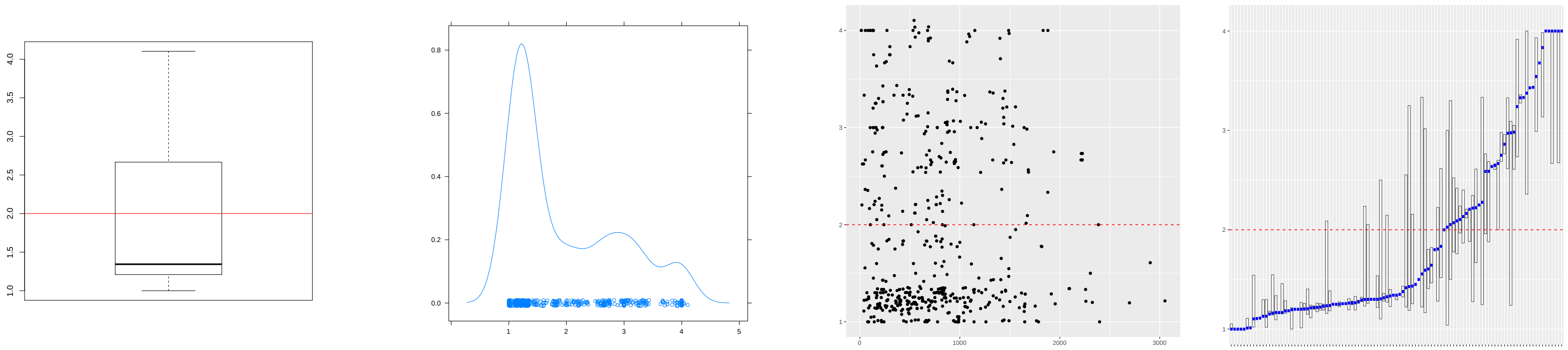

Sample	R22
Mean	1.63
Median	1.36
% <2	78.5

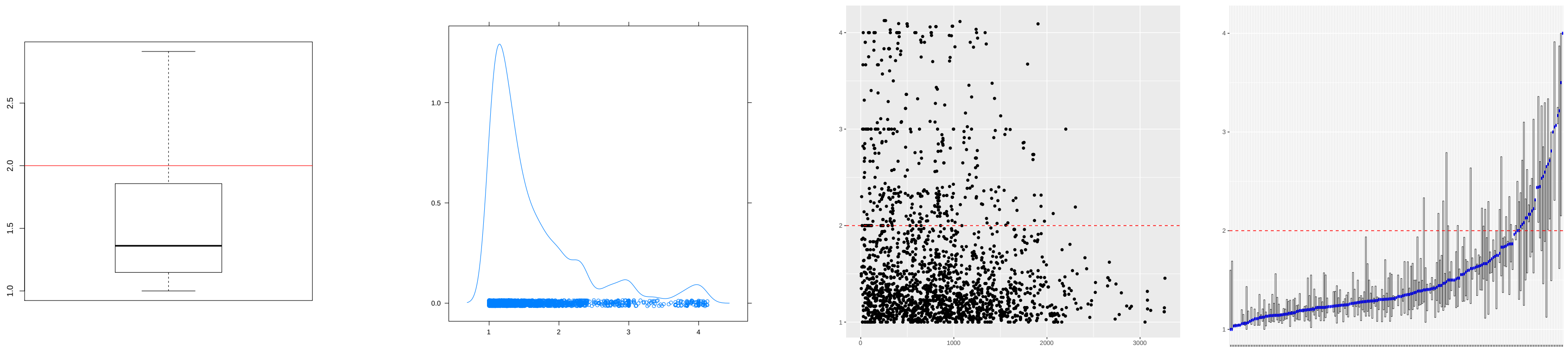

Sample	R78
Mean	1.89
Median	1.52
% <2	67.6

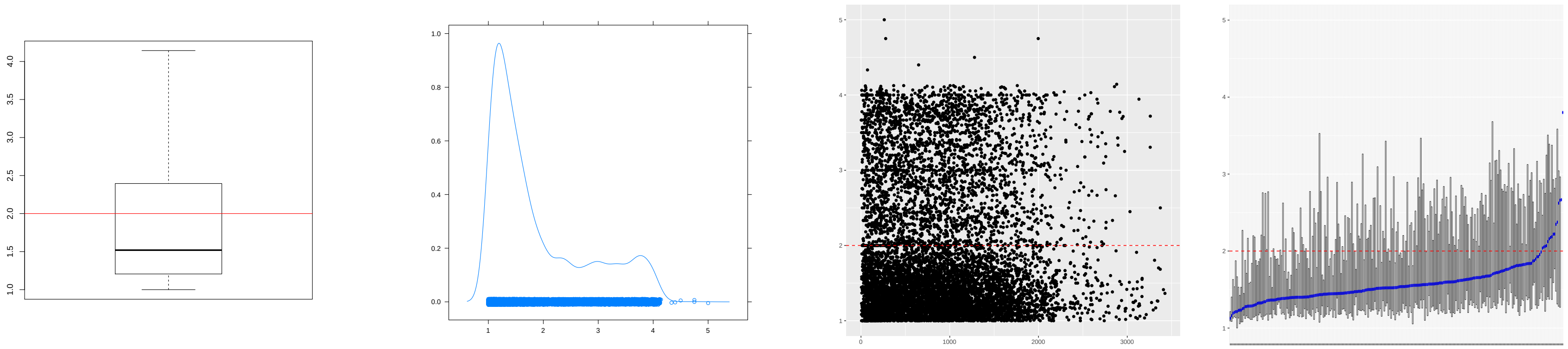

Sample	S71
Mean	1.94
Median	1.53
% <2	65.6

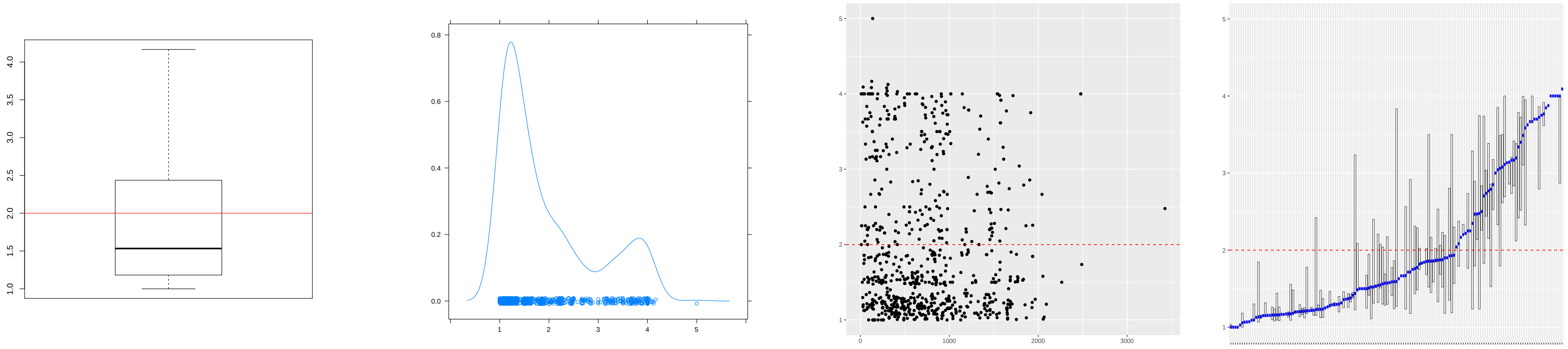

Sample	R14
Mean	1.86
Median	1.62
% <2	67.9

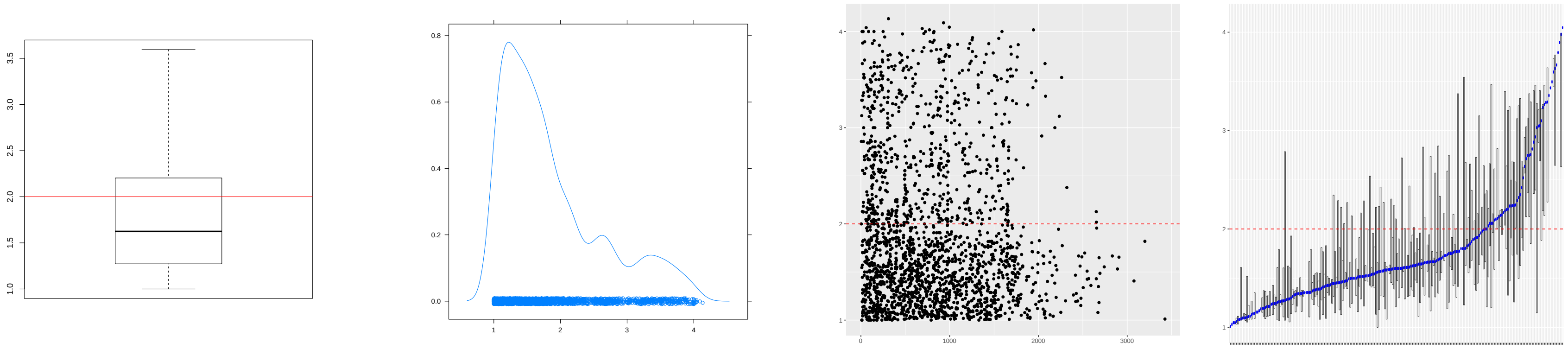

Sample	R129
Mean	2.43
Median	2.13
% <2	42.6

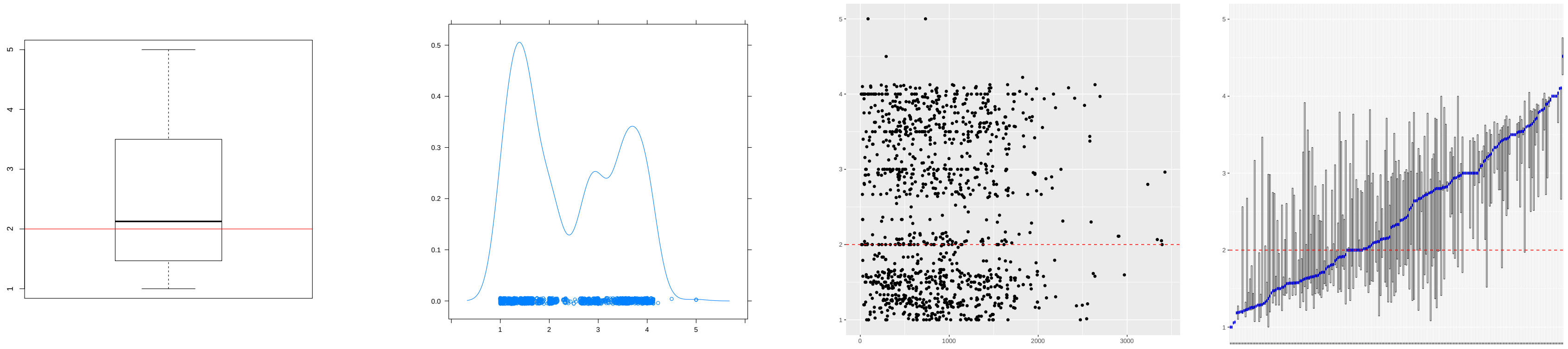

Sample	S62
Mean	2.24
Median	2.18
% <2	43.6

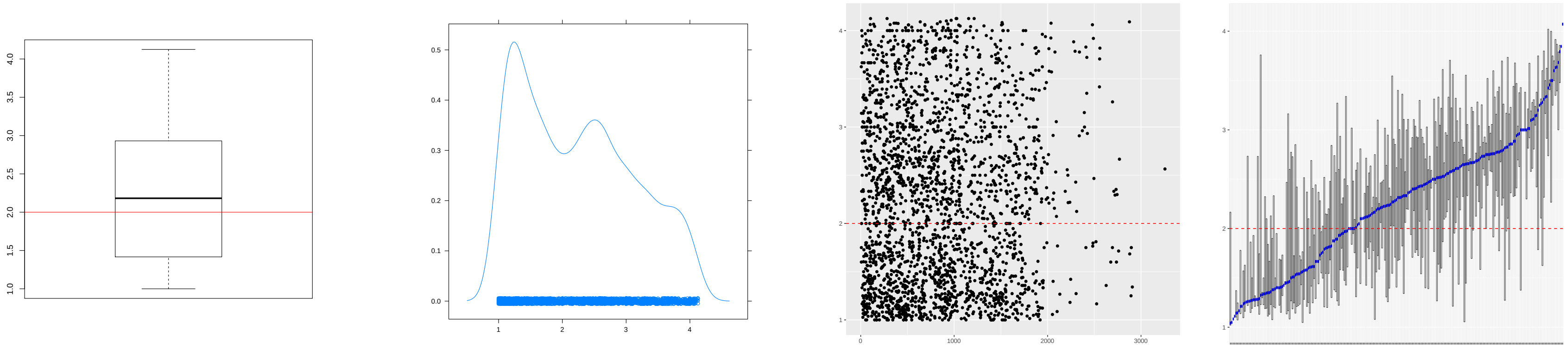

Sample	R95
Mean	2.45
Median	2.18
% <2	47.4

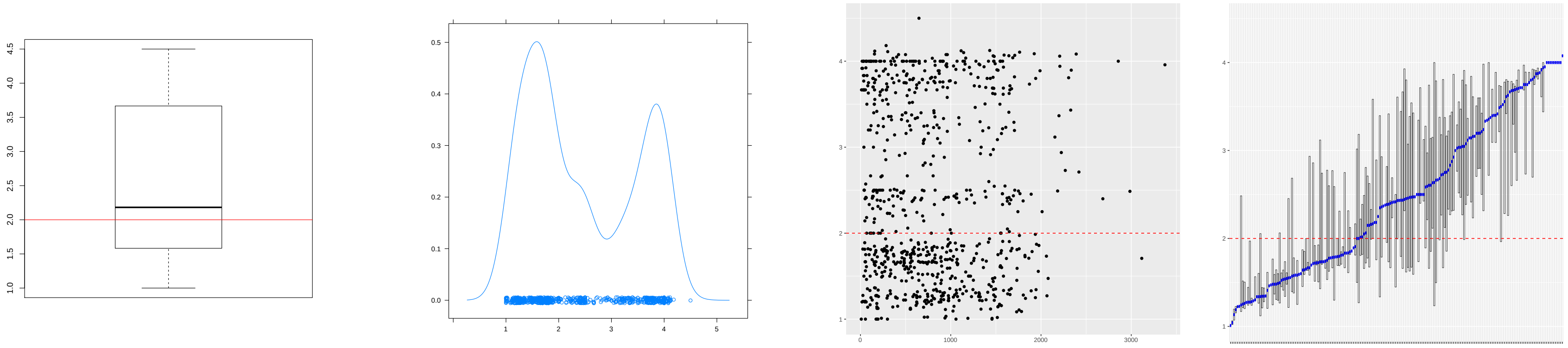

Sample	S61
Mean	2.32
Median	2.19
% <2	36.1

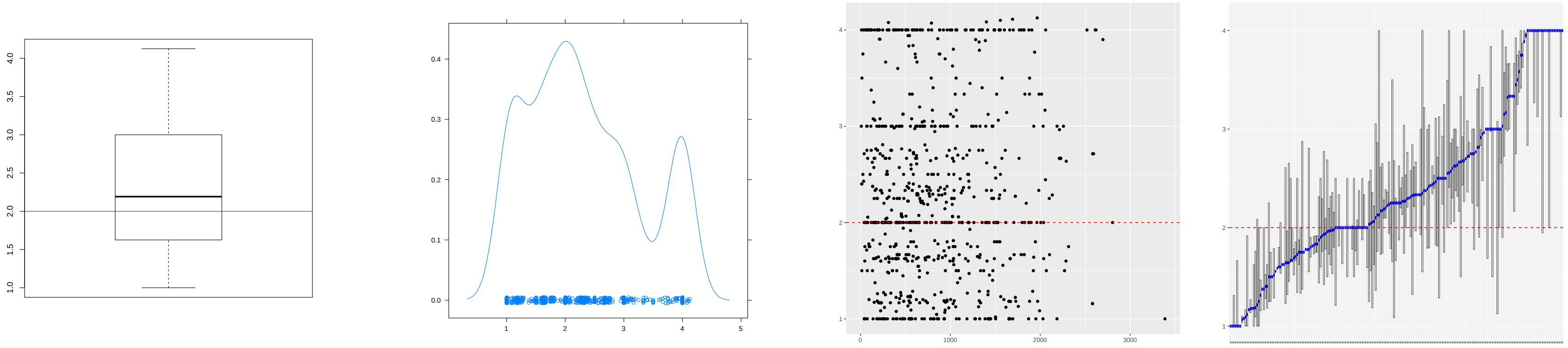

Sample	R05
Mean	2.22
Median	2.19
% <2	44.2

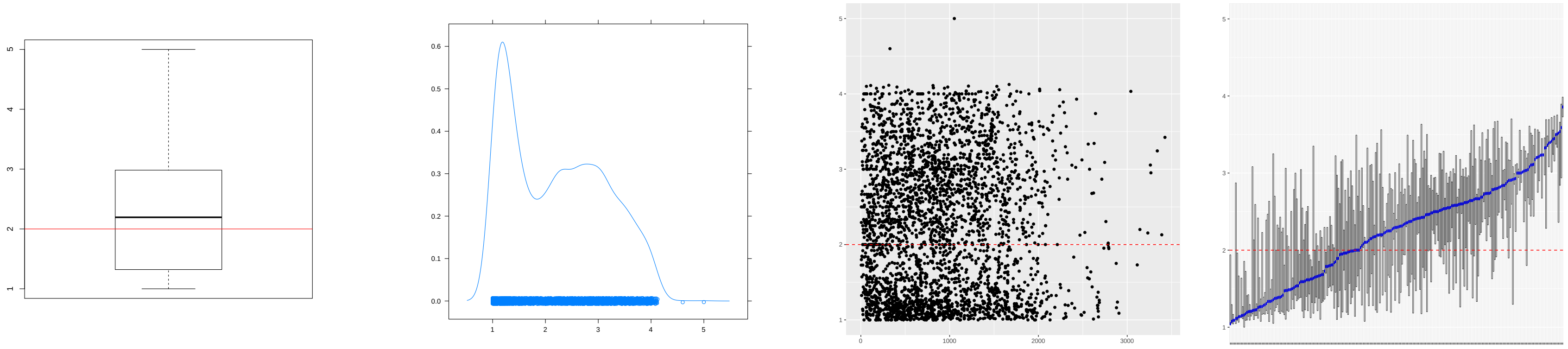

Sample	R11
Mean	2.30
Median	2.27
% <2	41.1

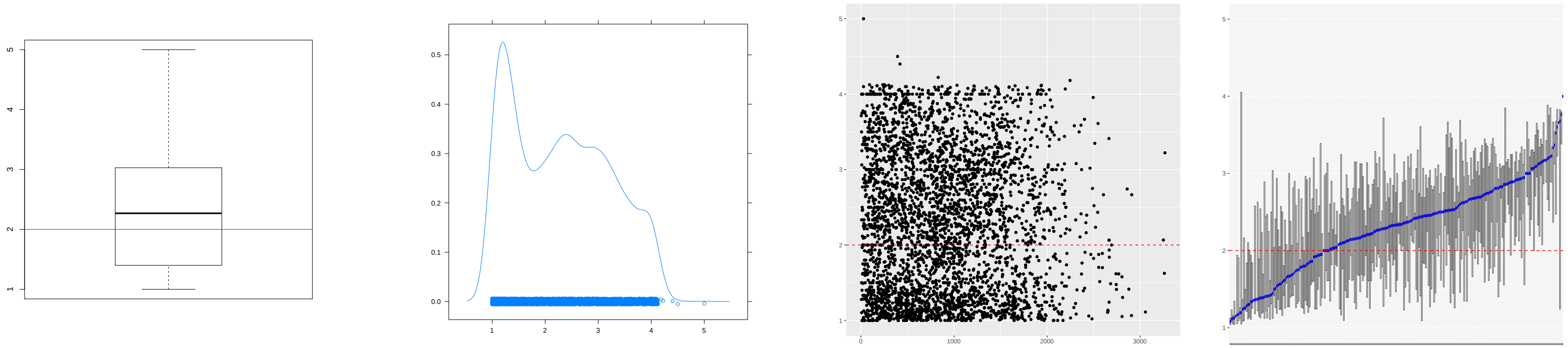

Sample	R21
Mean	2.25
Median	2.29
% <2	40.5

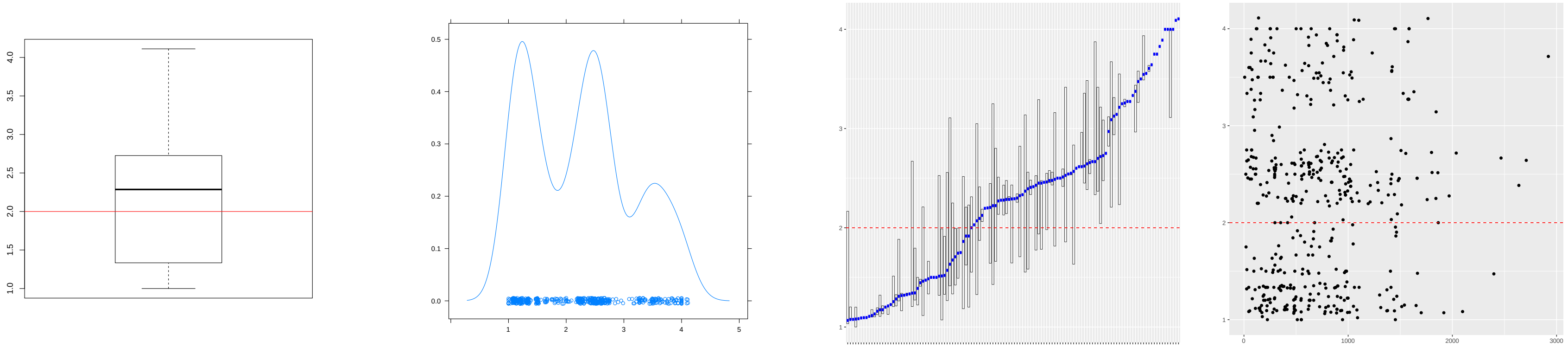

Sample	S53
Mean	2.38
Median	2.30
% <2	33.5

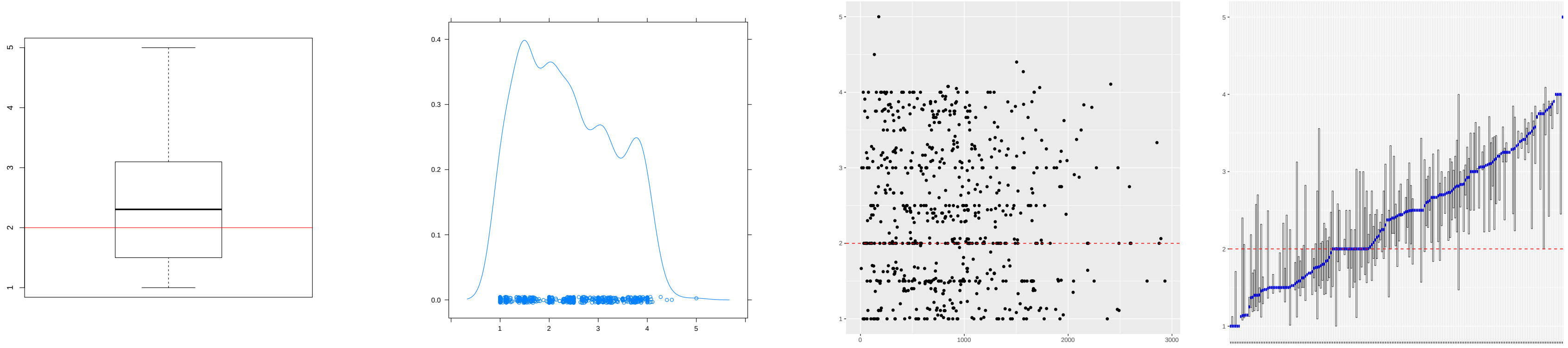

Sample	S54
Mean	2.50
Median	2.40
% <2	33.8

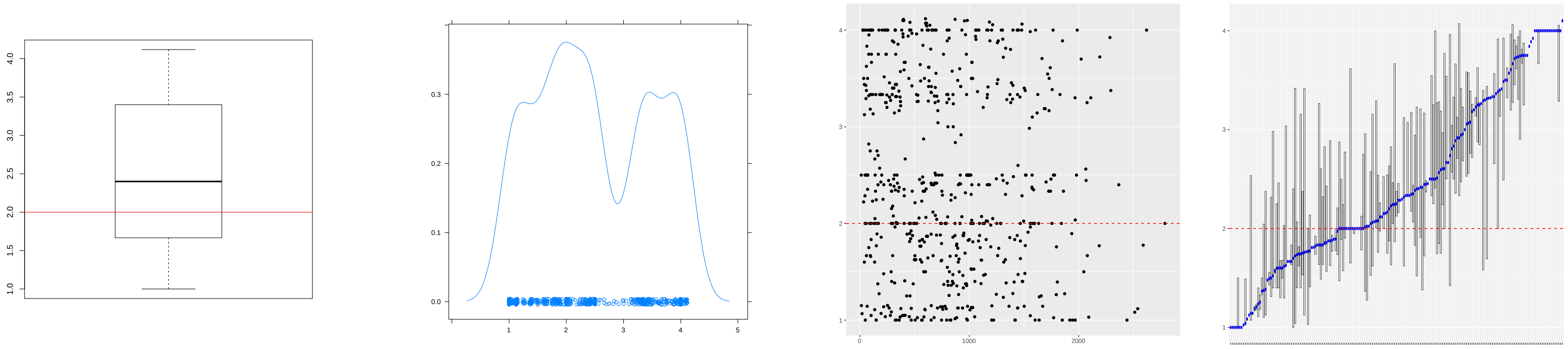

Sample	R06
Mean	2.35
Median	2.42
% <2	35.8

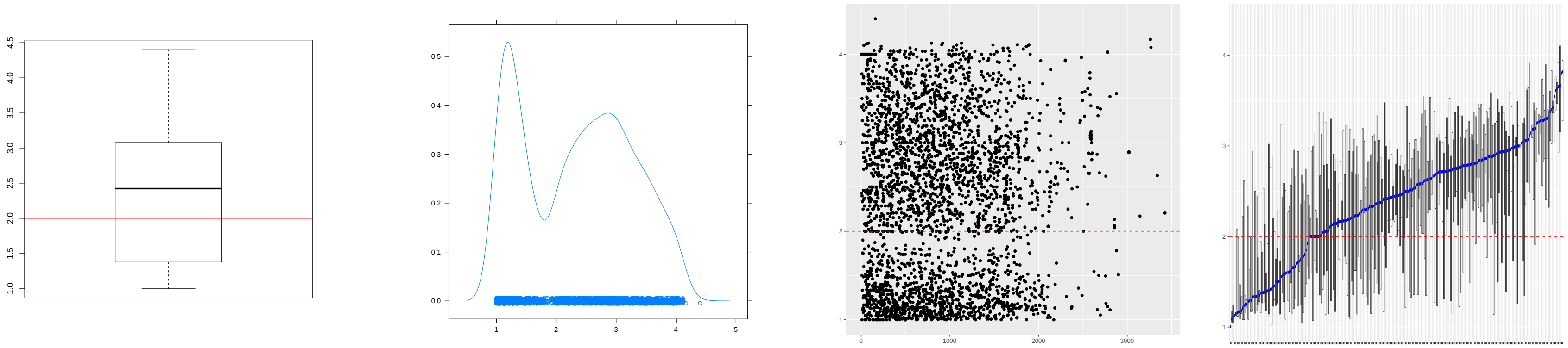

Sample	R25
Mean	2.49
Median	2.46
% <2	31.5

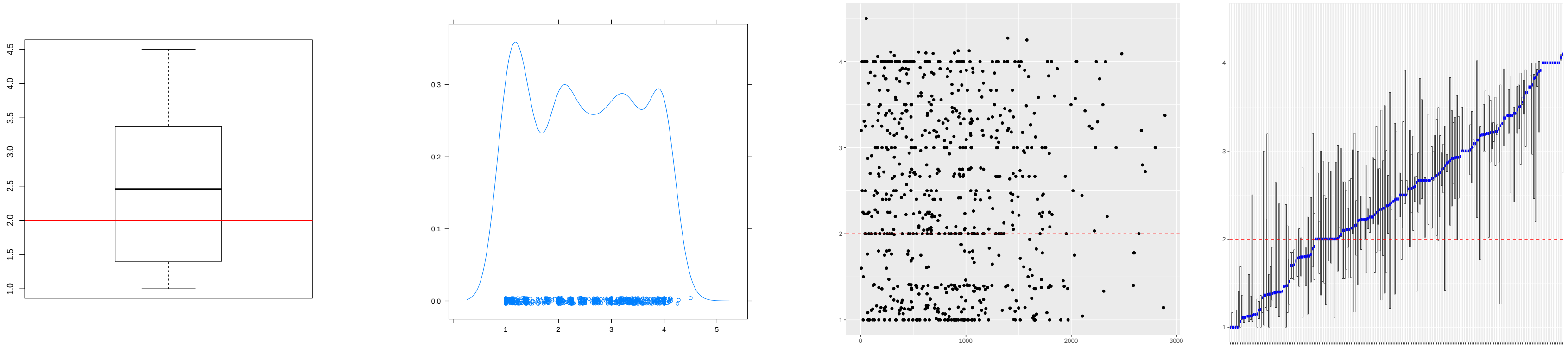

Sample	S80
Mean	2.46
Median	2.50
% <2	34.0

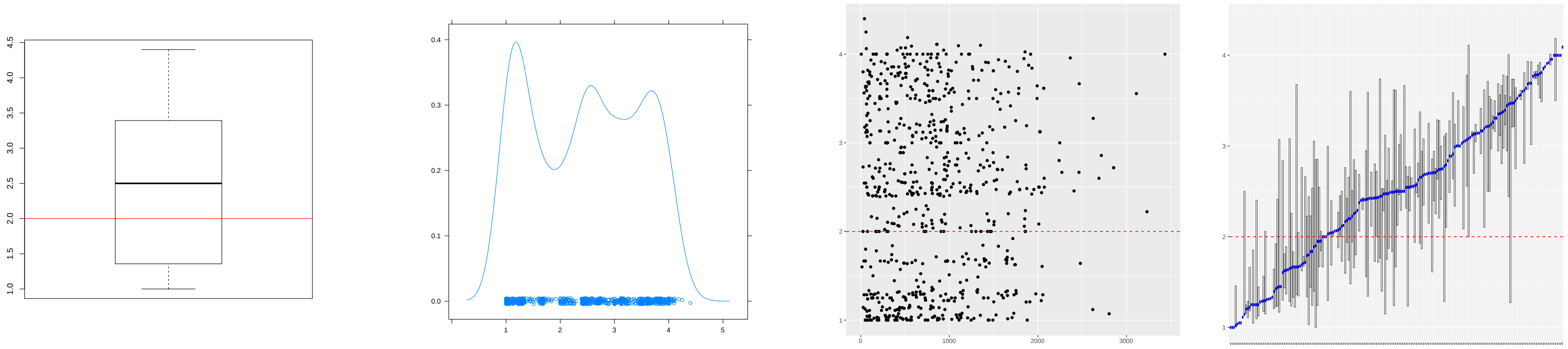

Sample	S64
Mean	2.34
Median	2.54
% <2	37.7

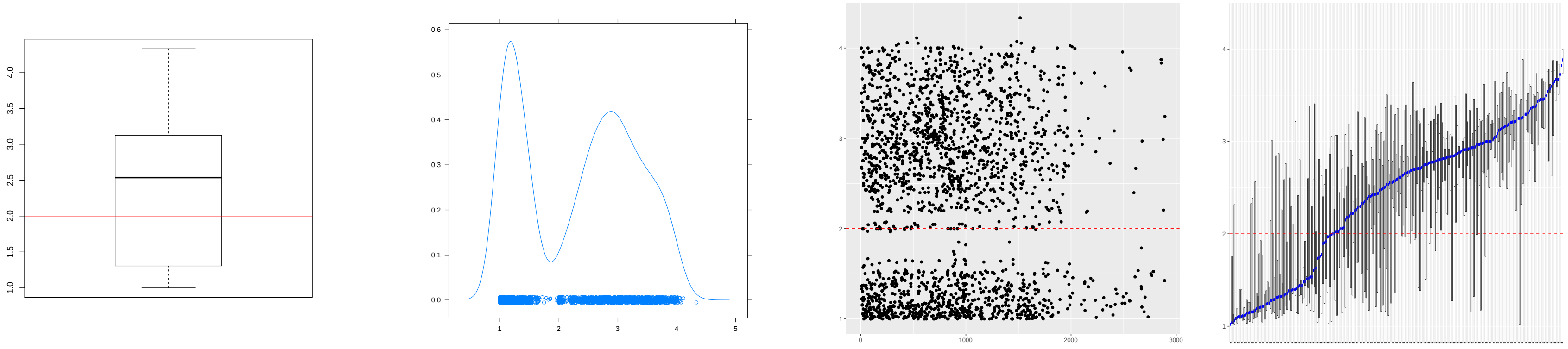

Sample	R73
Mean	2.60
Median	2.54
% <2	20.7

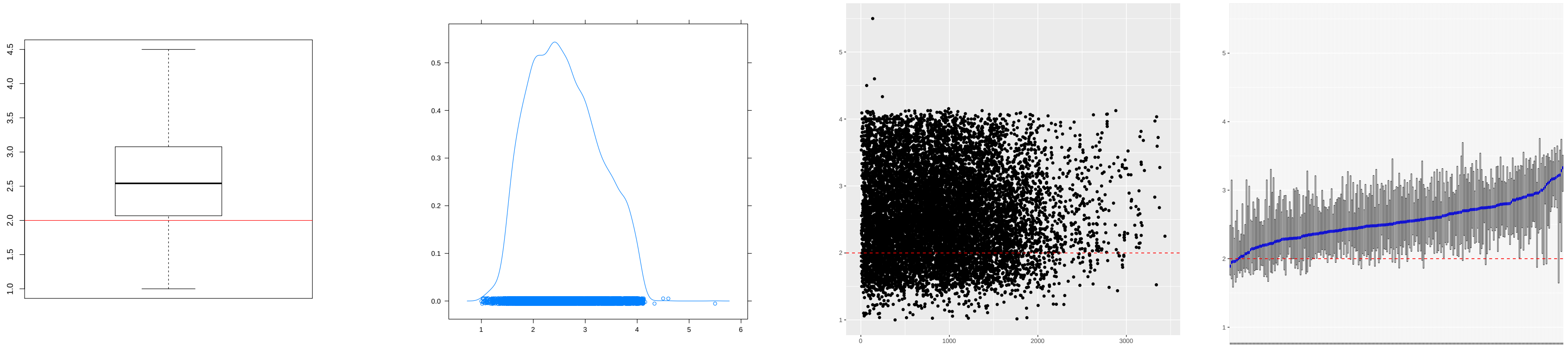

Sample	S25
Mean	2.43
Median	2.55
% <2	42.5

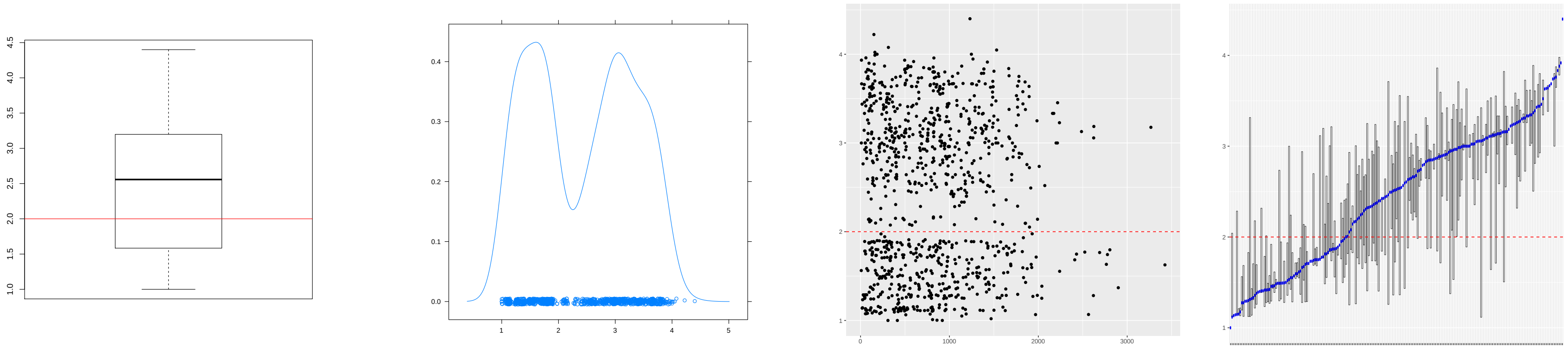

Sample	S42
Mean	2.48
Median	2.57
% <2	28.7

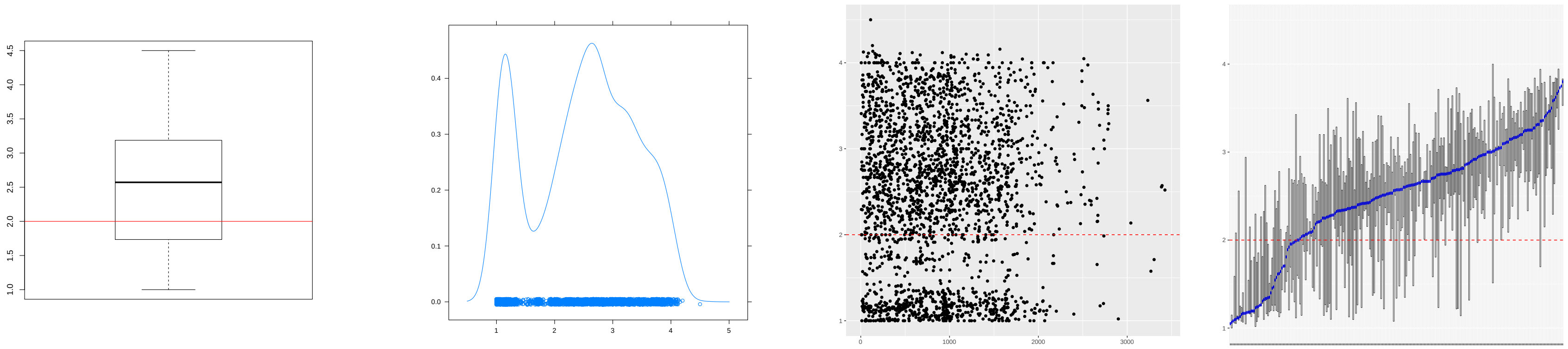

Sample	S33
Mean	2.62
Median	2.58
% <2	23.9

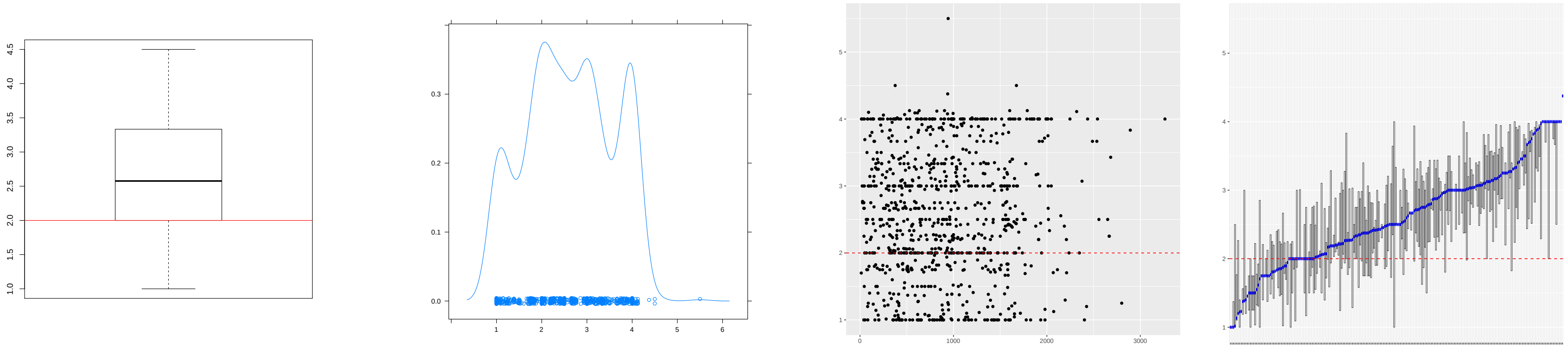

Sample	R27
Mean	2.46
Median	2.66
% <2	33.0

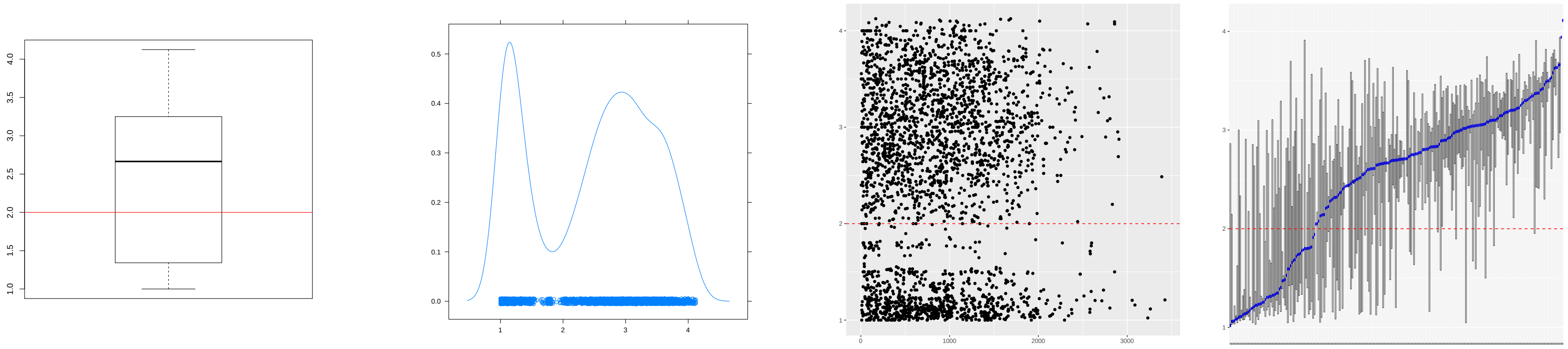

Sample	S35
Mean	2.57
Median	2.67
% <2	33.0

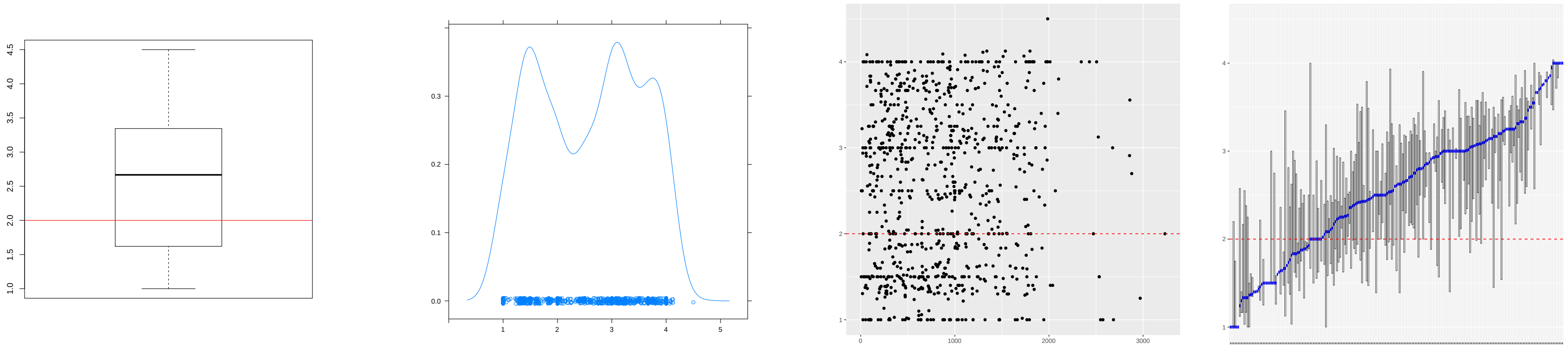

Sample	S79
Mean	2.48
Median	2.67
% <2	27.3

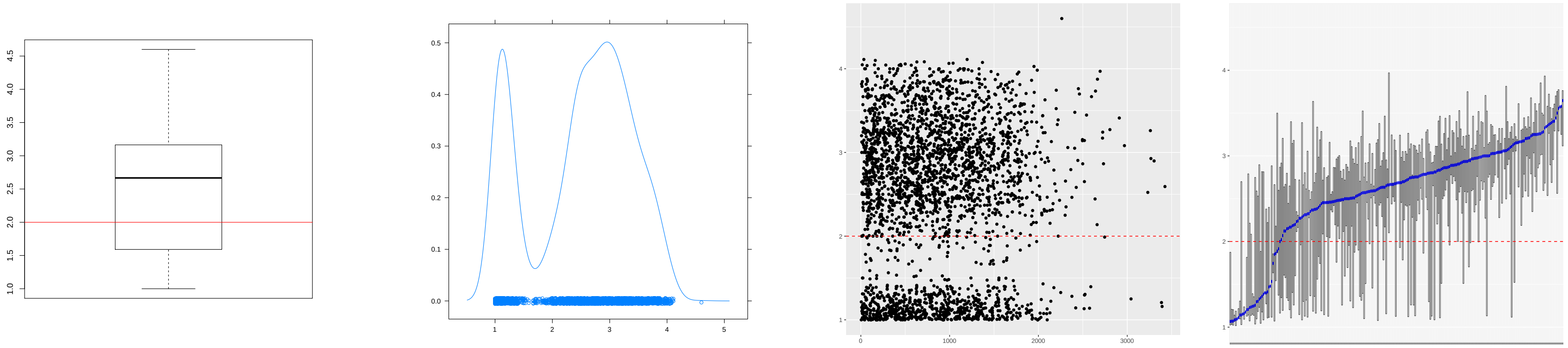

Sample	R28
Mean	2.51
Median	2.67
% <2	26.7

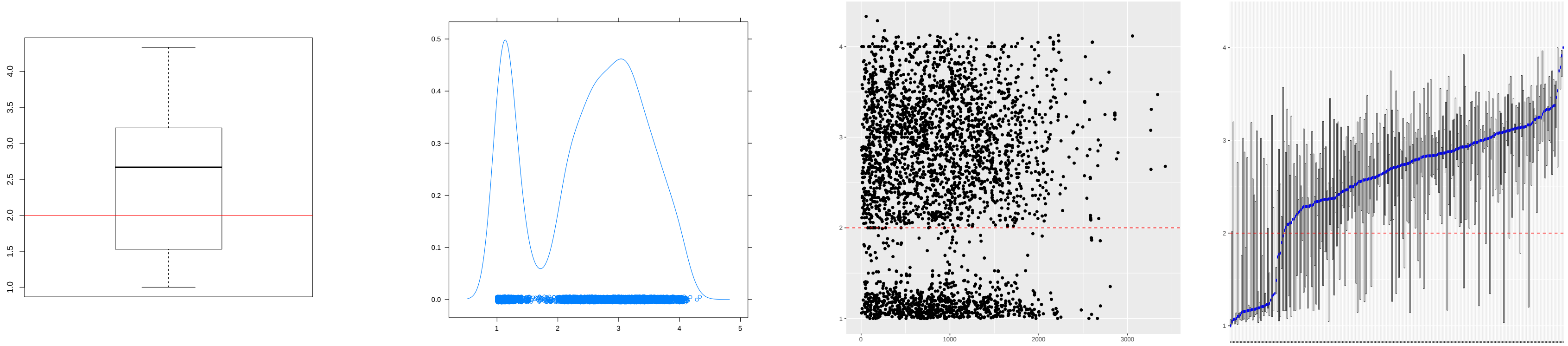

Sample	R01
Mean	2.41
Median	2.68
% <2	32.3

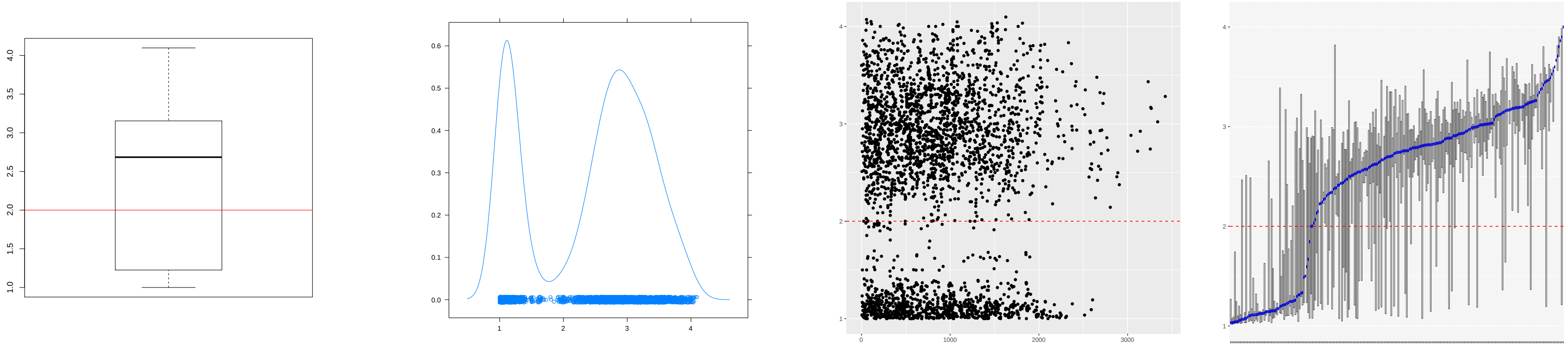

Sample	R75
Mean	2.71
Median	2.69
% <2	19.8

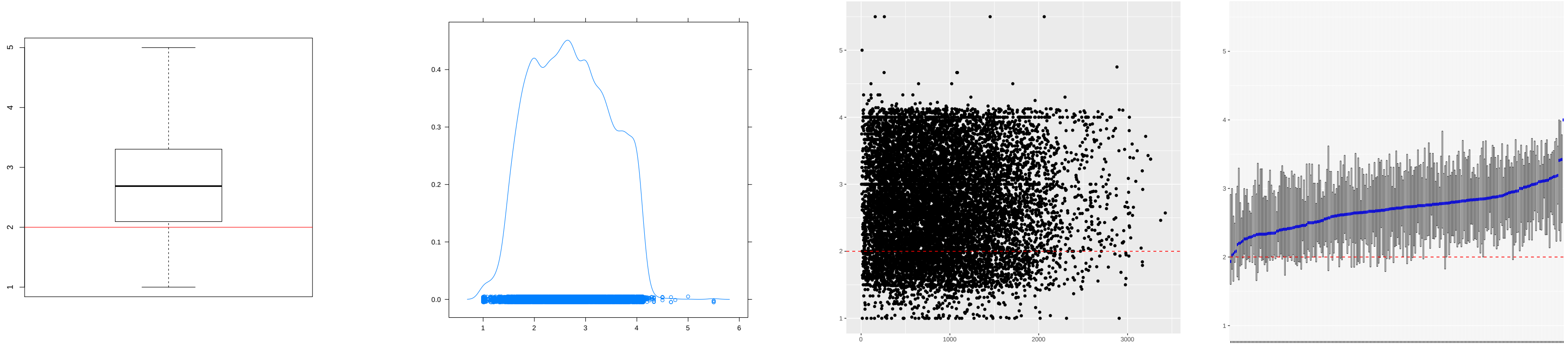

Sample	P06
Mean	2.51
Median	2.72
% <2	25.8

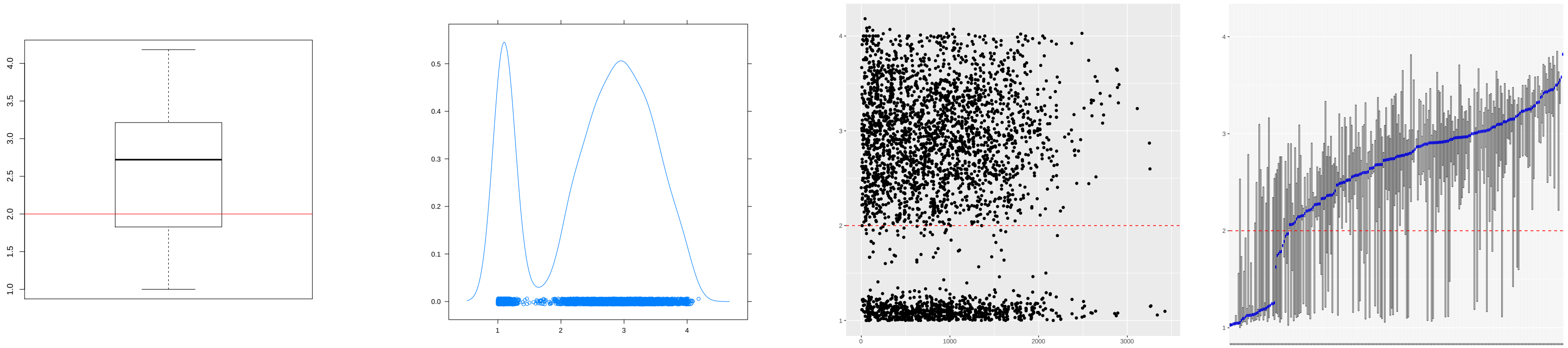

Sample	R31
Mean	2.41
Median	2.72
% <2	31.1

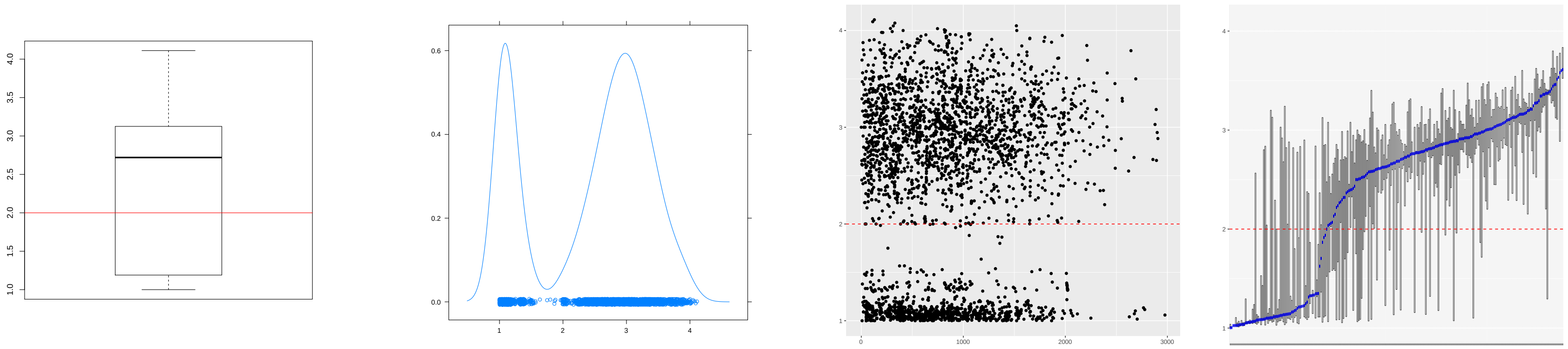

Sample	R13
Mean	2.72
Median	2.73
% <2	21.1

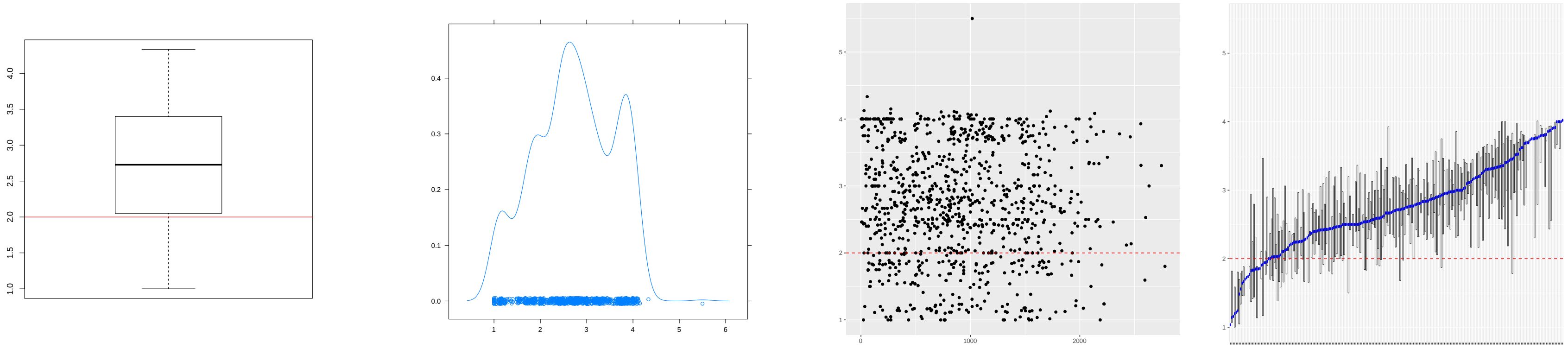

Sample	R04
Mean	2.60
Median	2.73
% <2	23.2

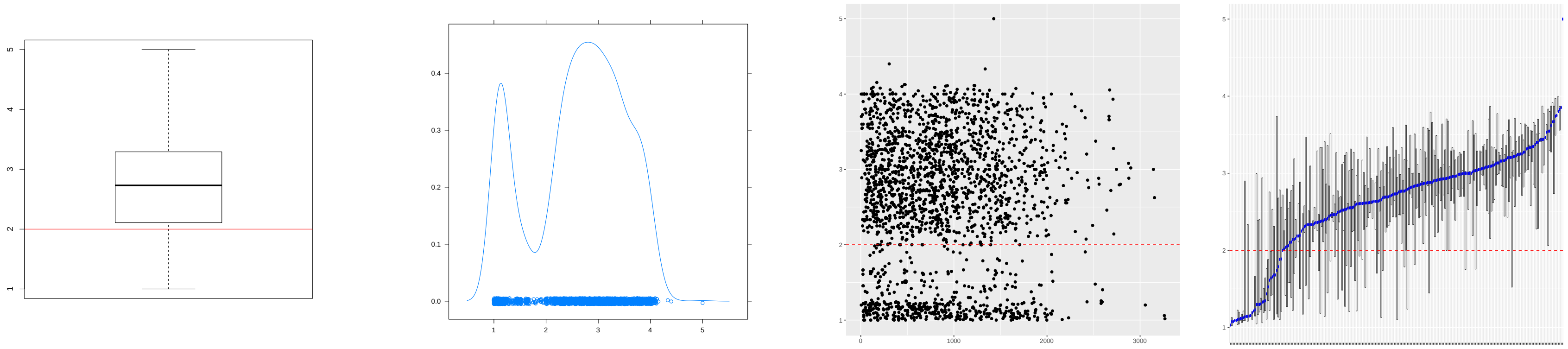

Sample	R30
Mean	2.59
Median	2.73
% <2	24.8

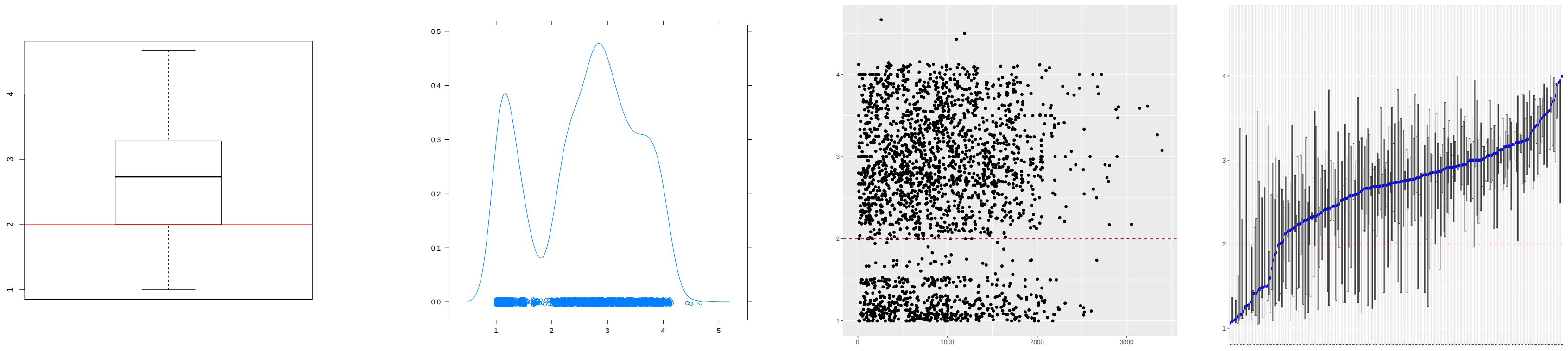

Sample	R77
Mean	2.76
Median	2.73
% <2	17.6

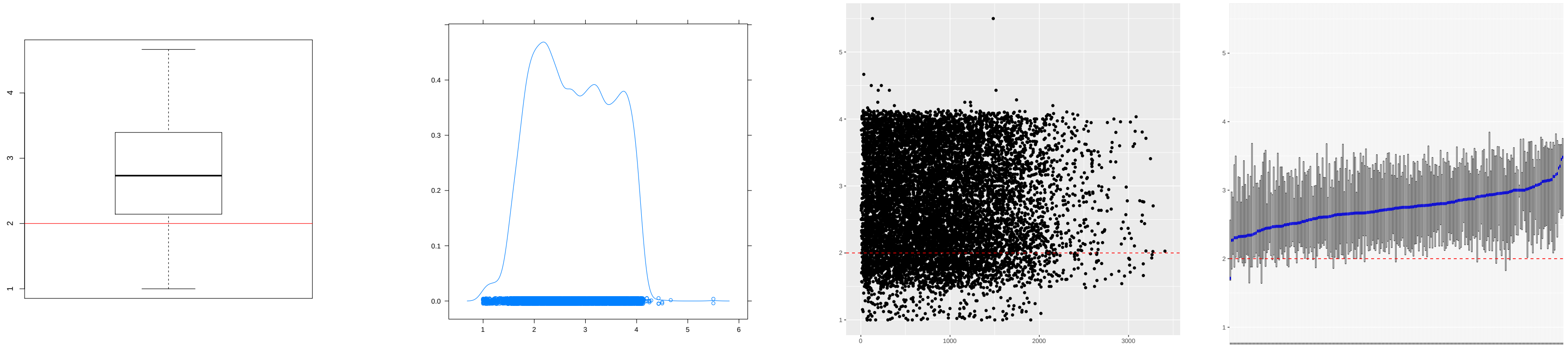

Sample	S28
Mean	2.66
Median	2.74
% <2	25.6

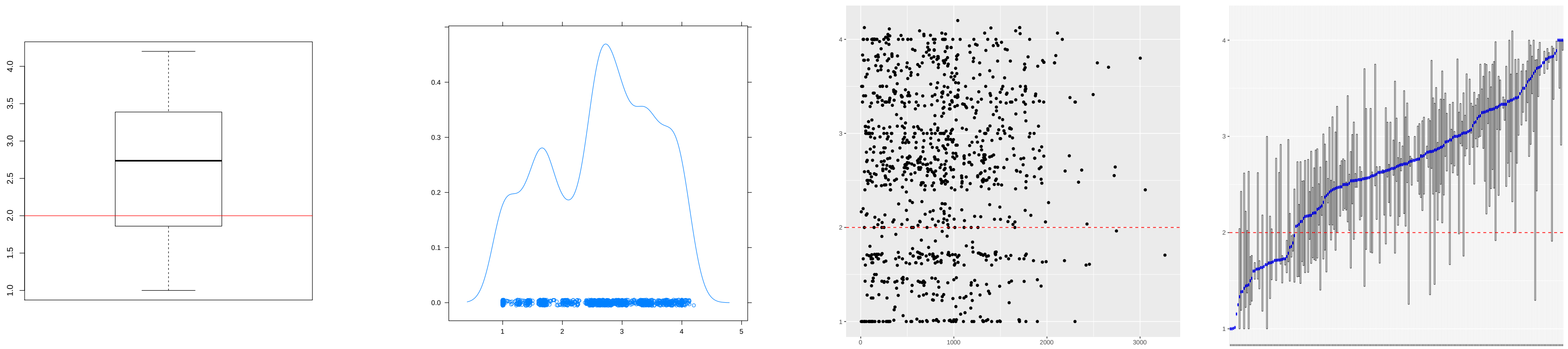

Sample	R32
Mean	2.62
Median	2.74
% <2	226

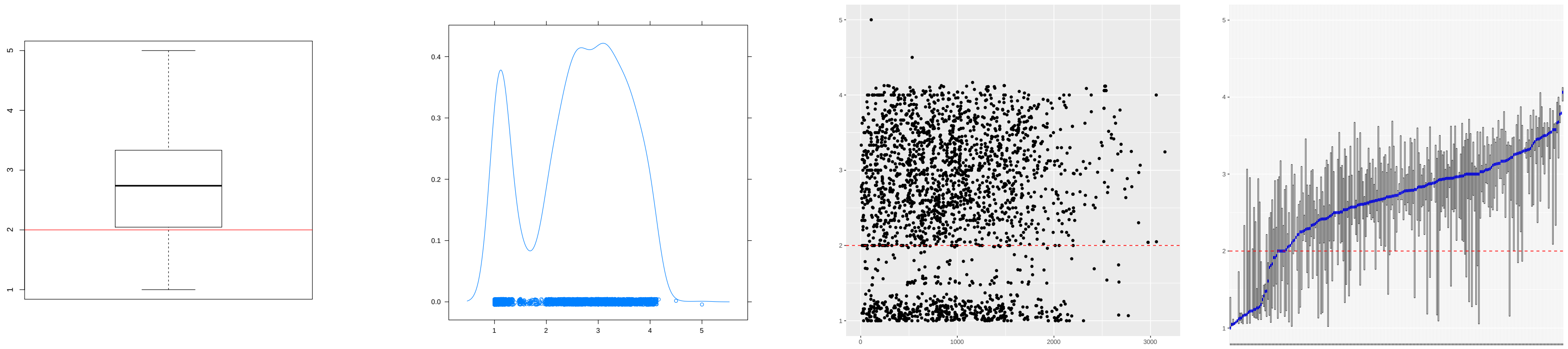

Sample	S63
Mean	2.59
Median	2.74
% <2	24.4

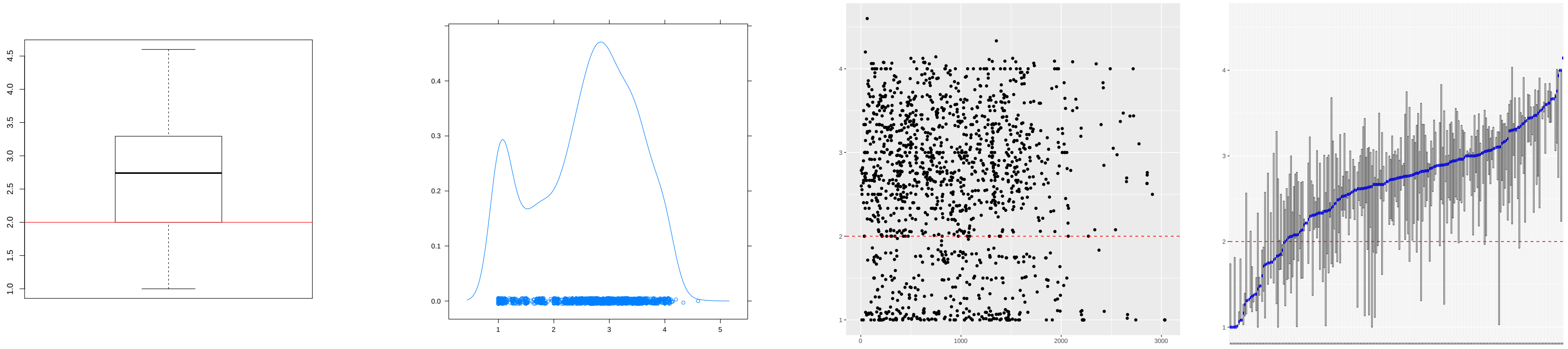

Sample	R29
Mean	2.57
Median	2.75
% <2	24.14

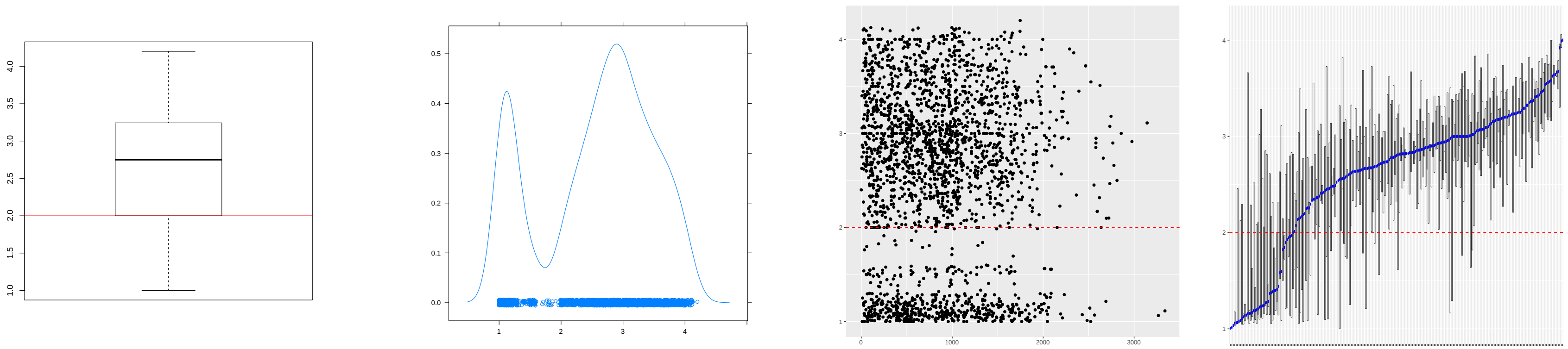

Sample	S73
Mean	2.63
Median	2.80
% <2	23.5

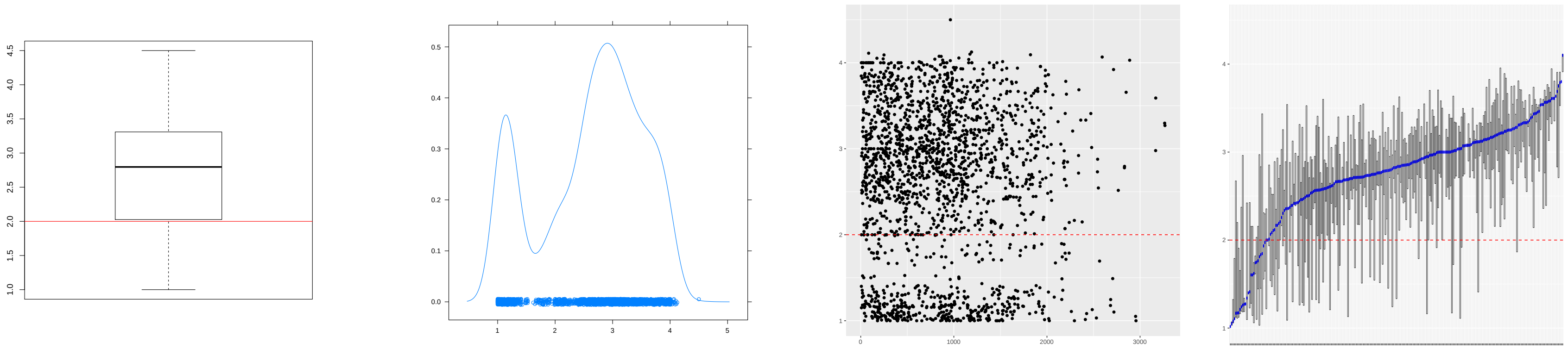

Sample	S76
Mean	2.72
Median	2.82
% <2	16.2

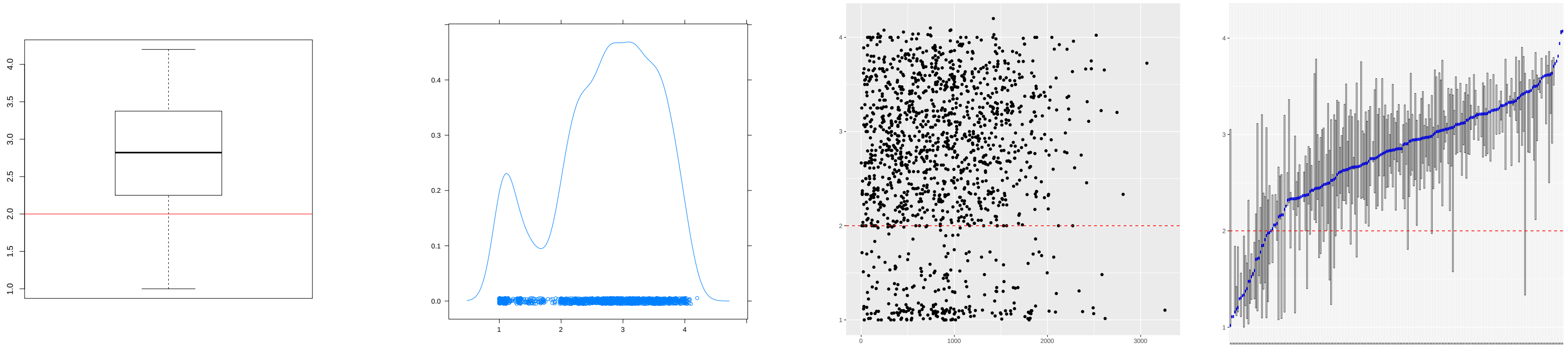

Sample	R23
Mean	2.76
Median	2.89
% <2	23.6

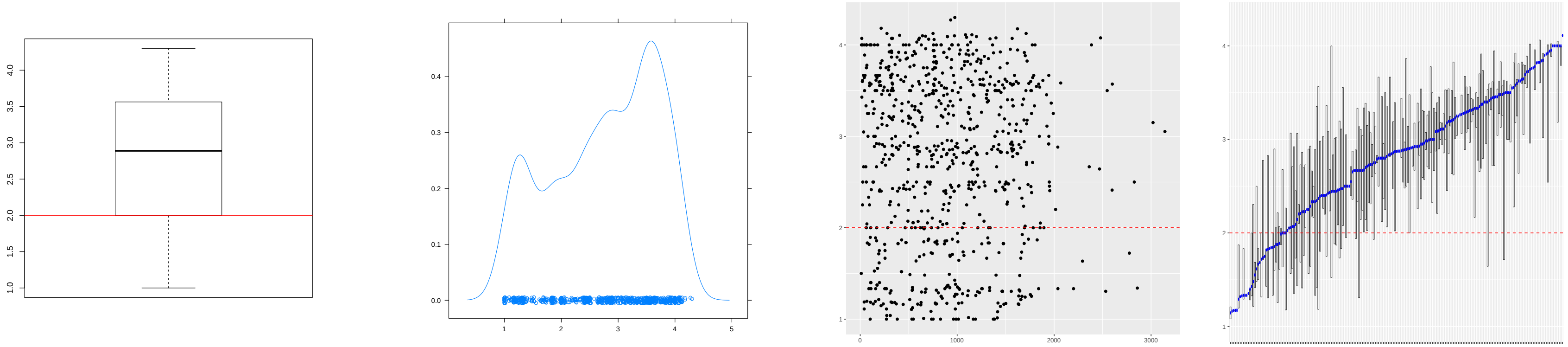

Sample	S34
Mean	2.84
Median	2.90
% <2	12.1

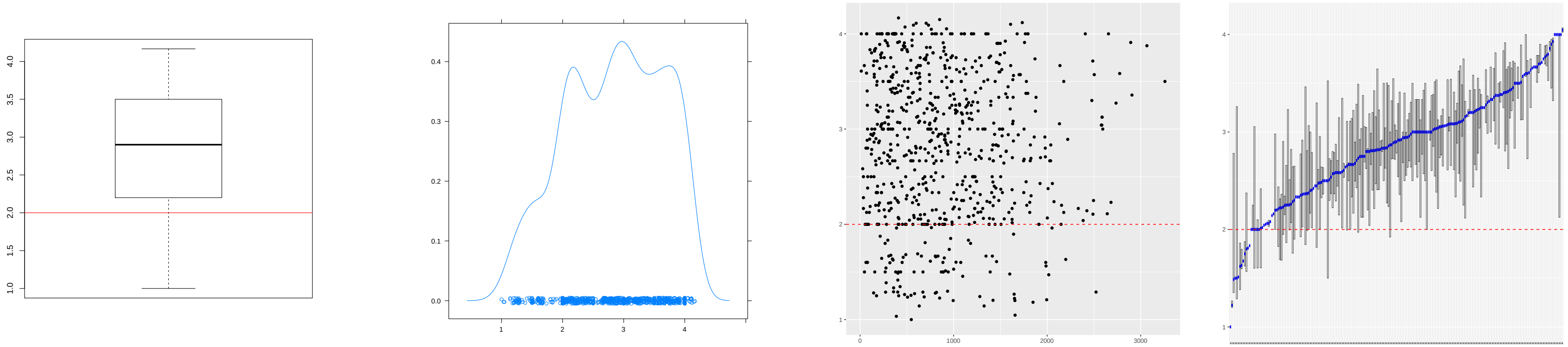

Sample	R84
Mean	2.89
Median	2.90
% <2	12.9

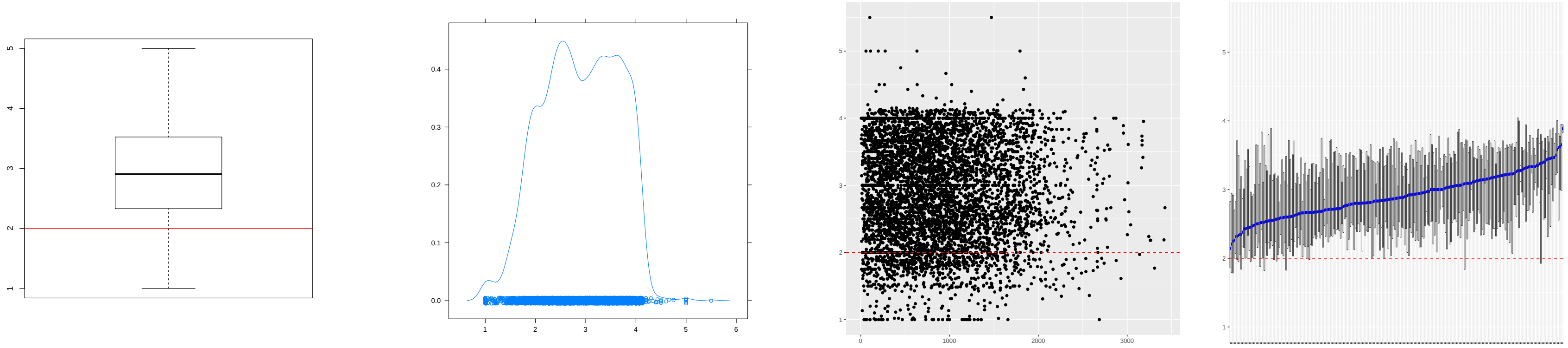

Sample	R10
Mean	2.90
Median	2.94
% <2	13.7

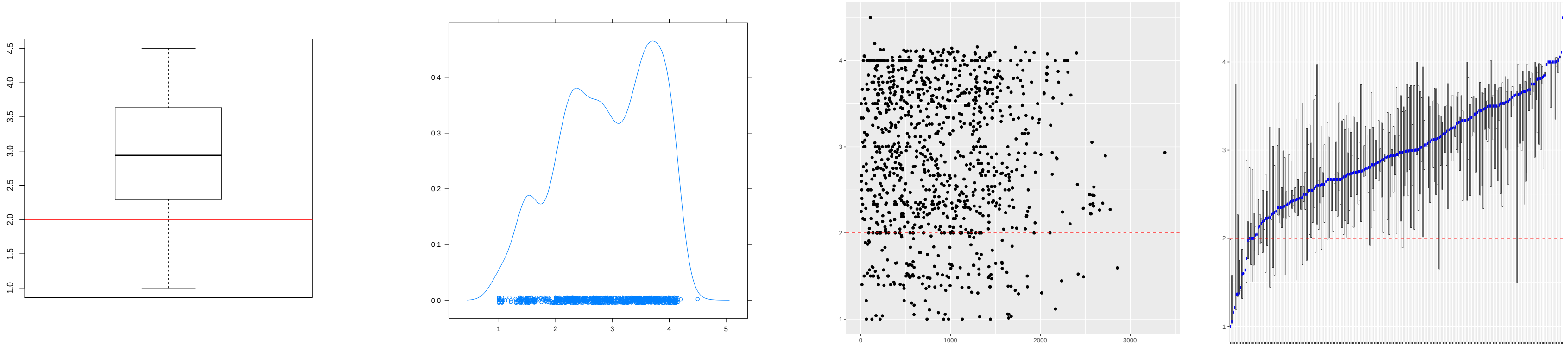

Sample	R07
Mean	2.81
Median	3.00
% <2	21.5

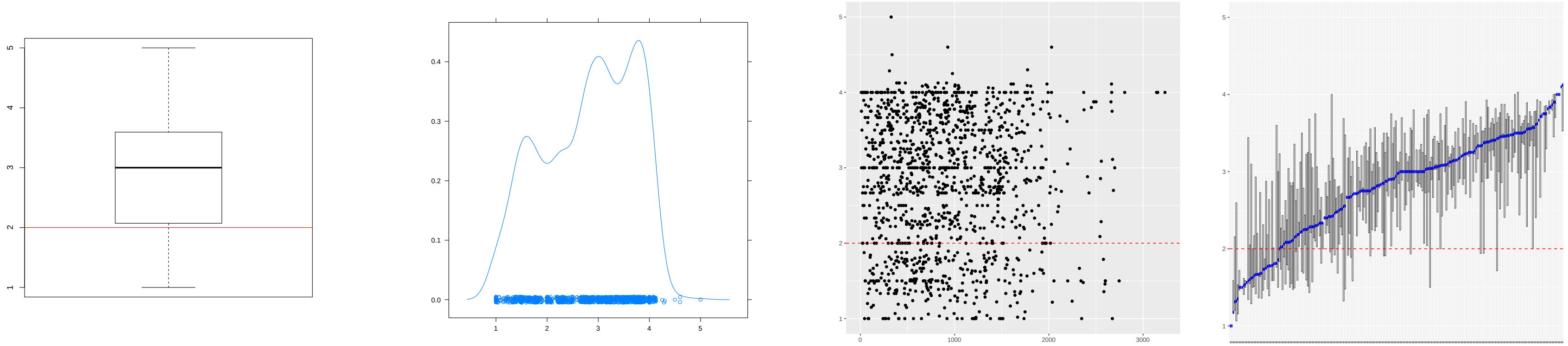

Sample	R74
Mean	2.95
Median	3.00
% <2	9.83

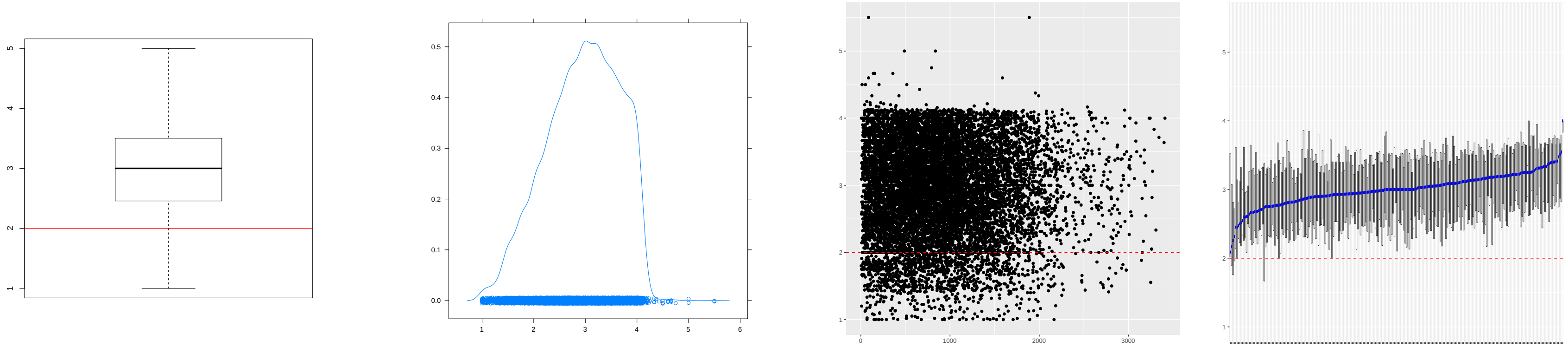

Sample	R17
Mean	2.95
Median	3.00
% <2	8.86

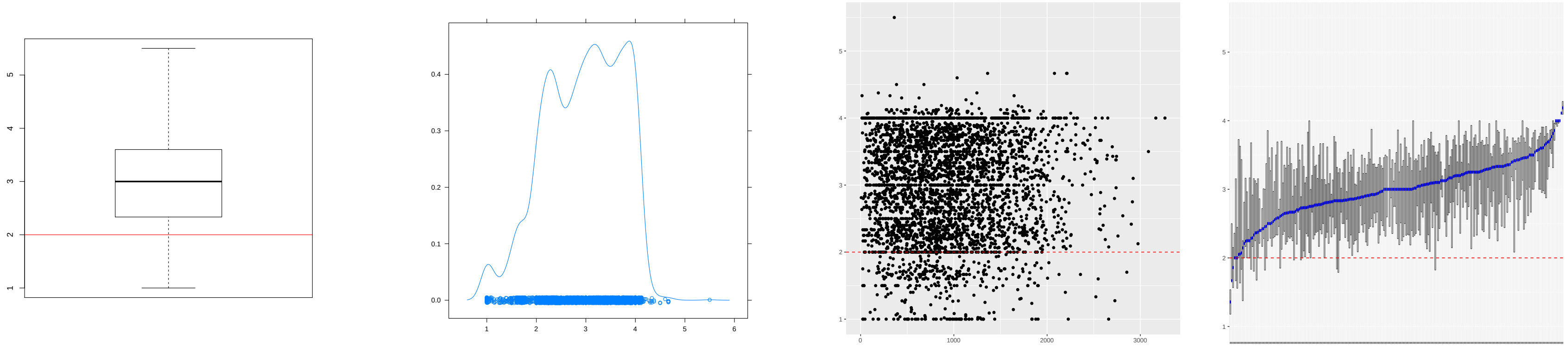

Sample	R08
Mean	3.03
Median	3.17
% <2	9.73

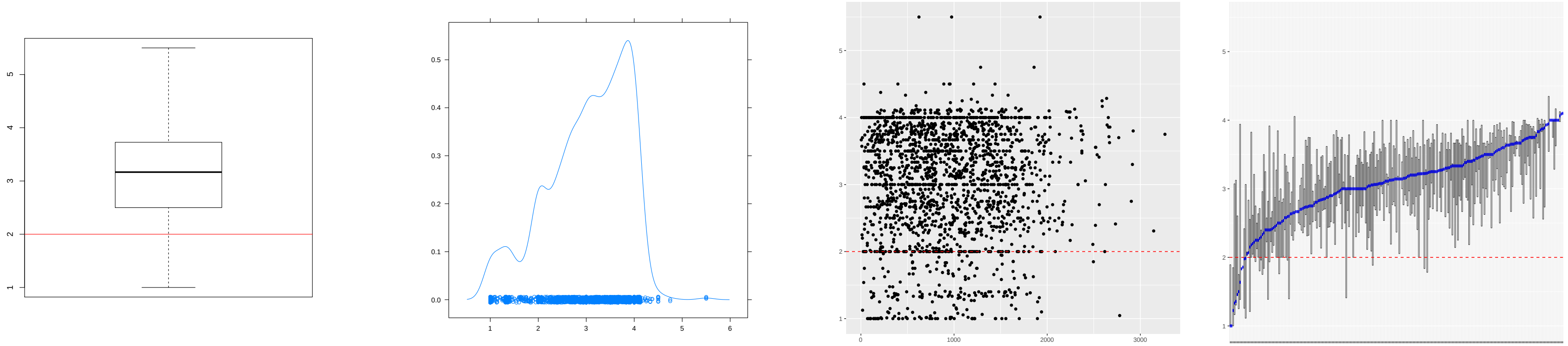

Sample	R96
Mean	2.83
Median	3.18
% <2	30.2

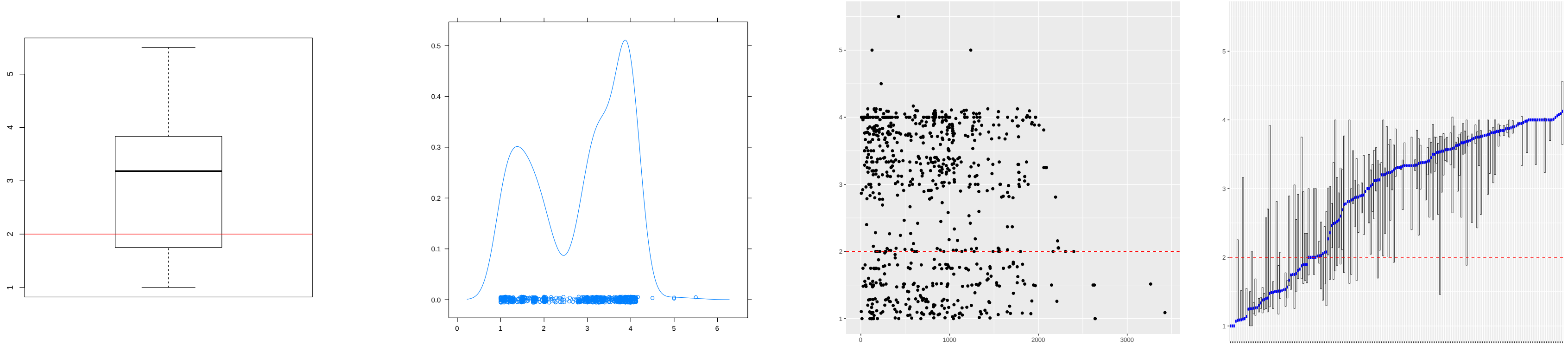

Sample	R94
Mean	3.02
Median	2.20
% <2	13.4

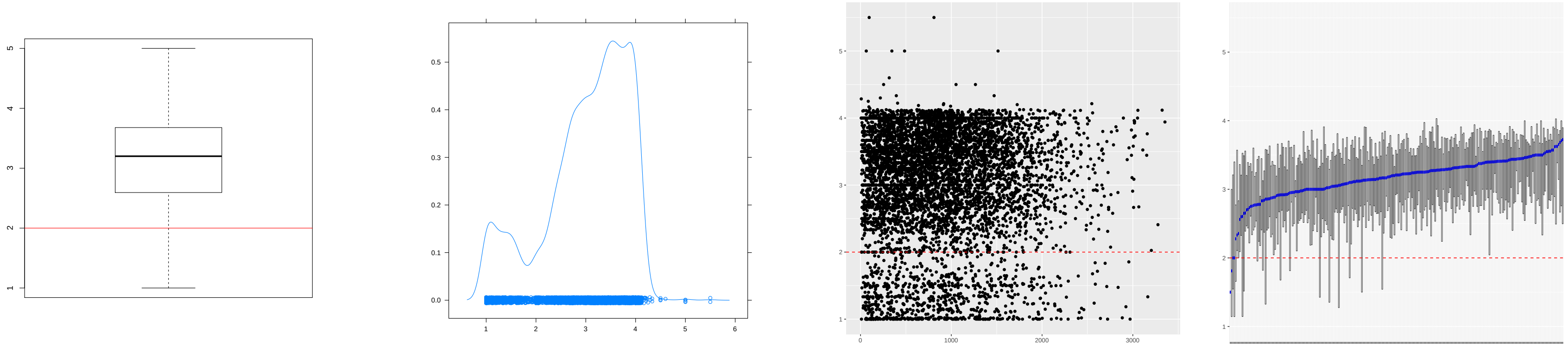

Sample	R18
Mean	3.03
Median	3.28
% <2	15.1

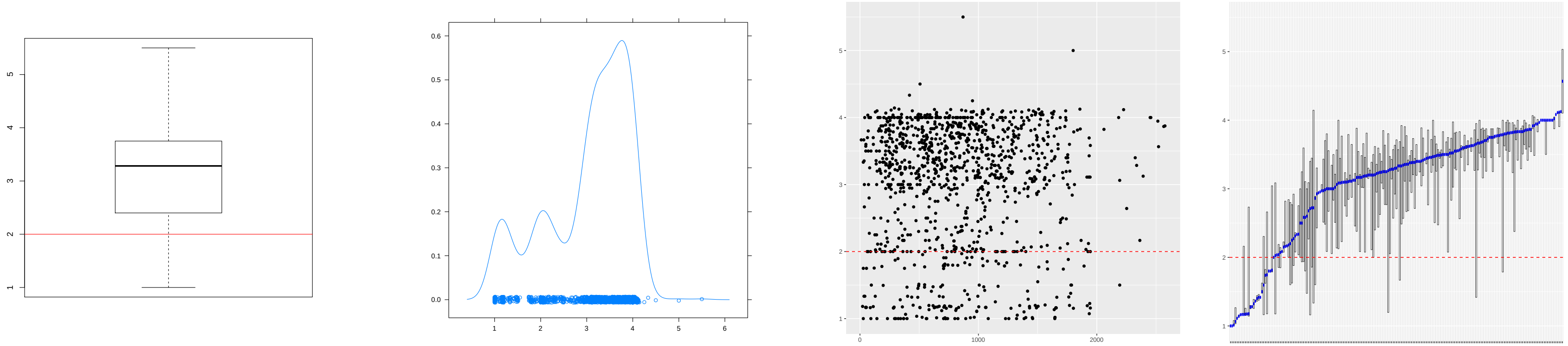

Sample	R86
Mean	3.15
Median	3.33
% <2	10.1

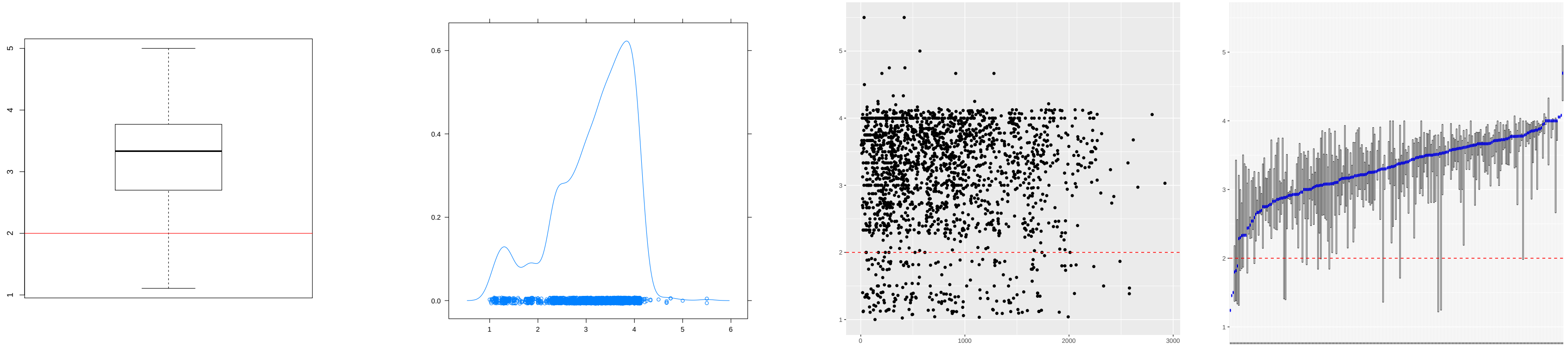

Sample	R76
Mean	3.15
Median	3.38
% <2	13.8

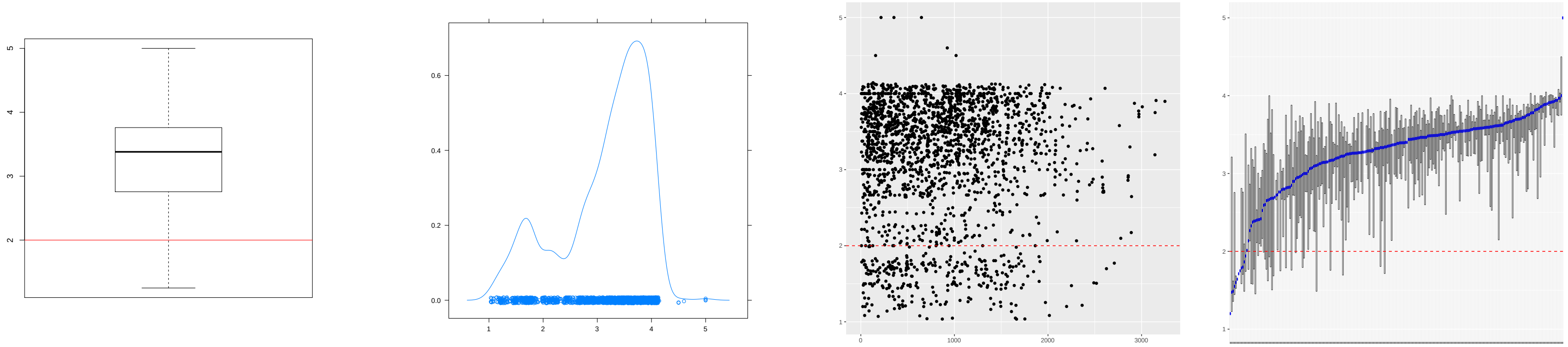

Dioscorea alata

Sample	Y05
Mean	1.56
Median	1.30
% <2	78.8

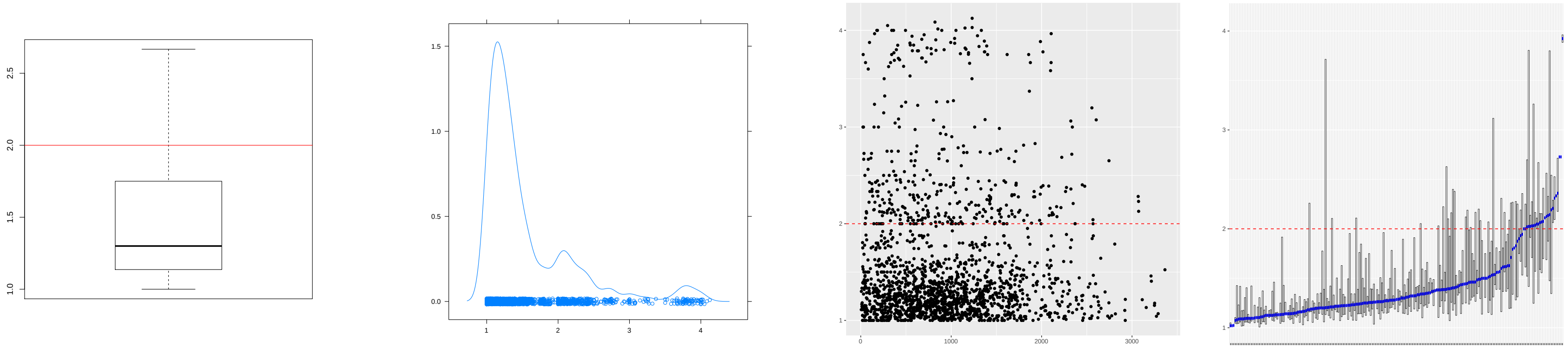

Sample	Y07
Mean	1.75
Median	1.32
% <2	73.3

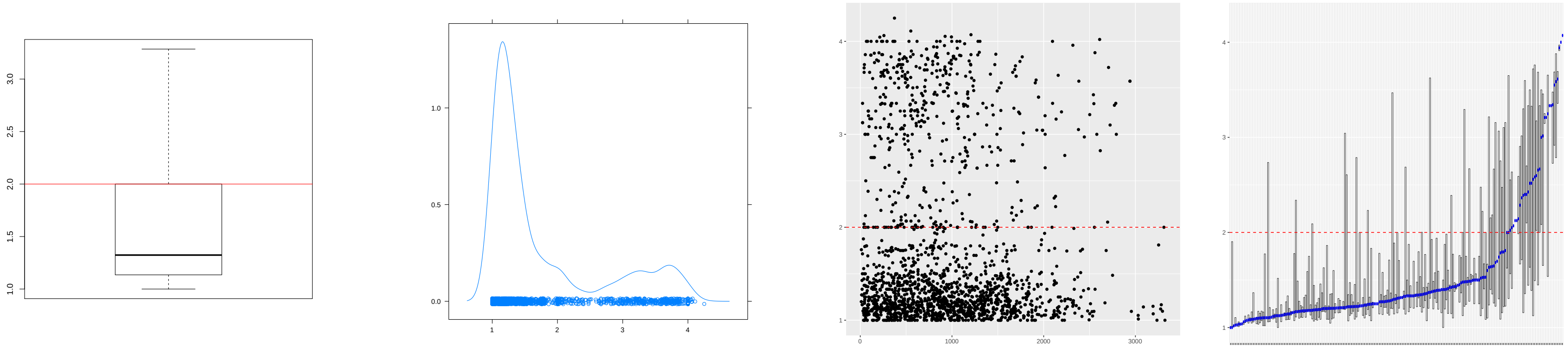

Sample	Y02
Mean	1.89
Median	1.52
% <2	67.2

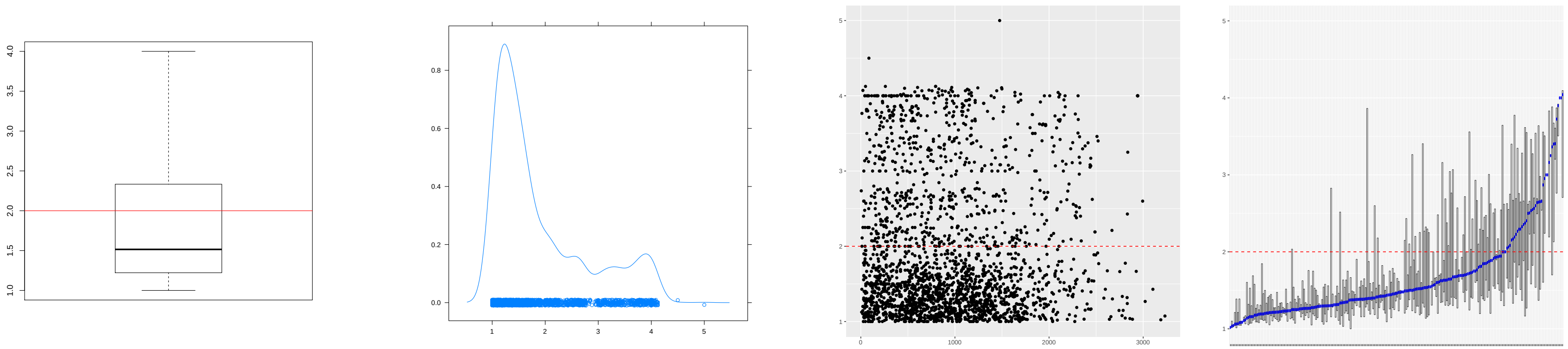

Sample	Y01
Mean	1.84
Median	1.58
% <2	66.2

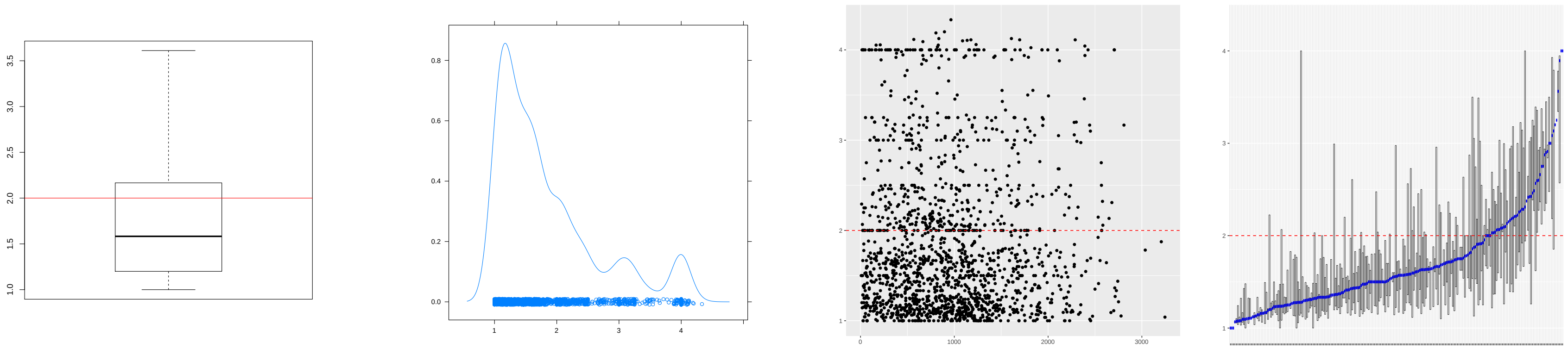

Sample	Y09
Mean	2.05
Median	1.50
% <2	59.1

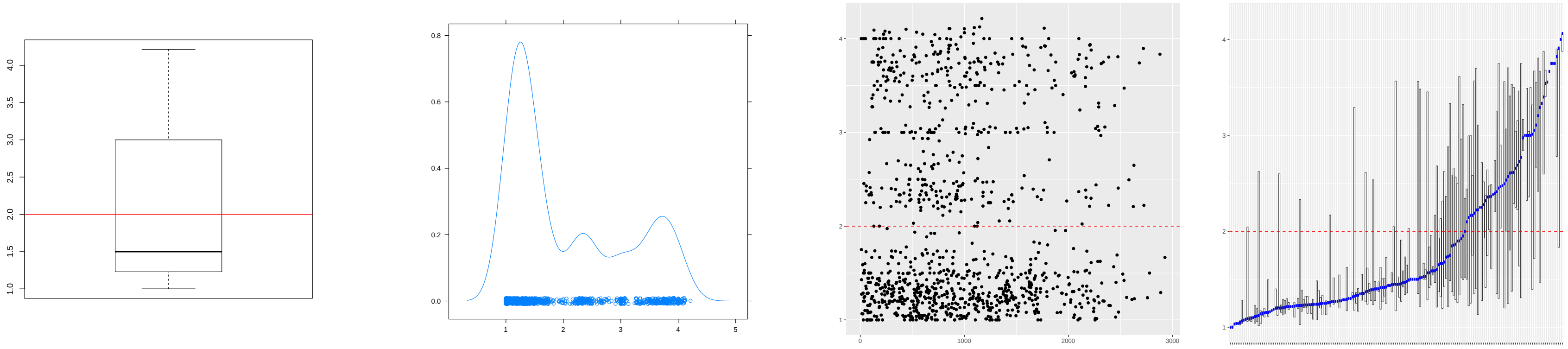

Sample	T38
Mean	2.37
Median	2.26
% <2	34.6

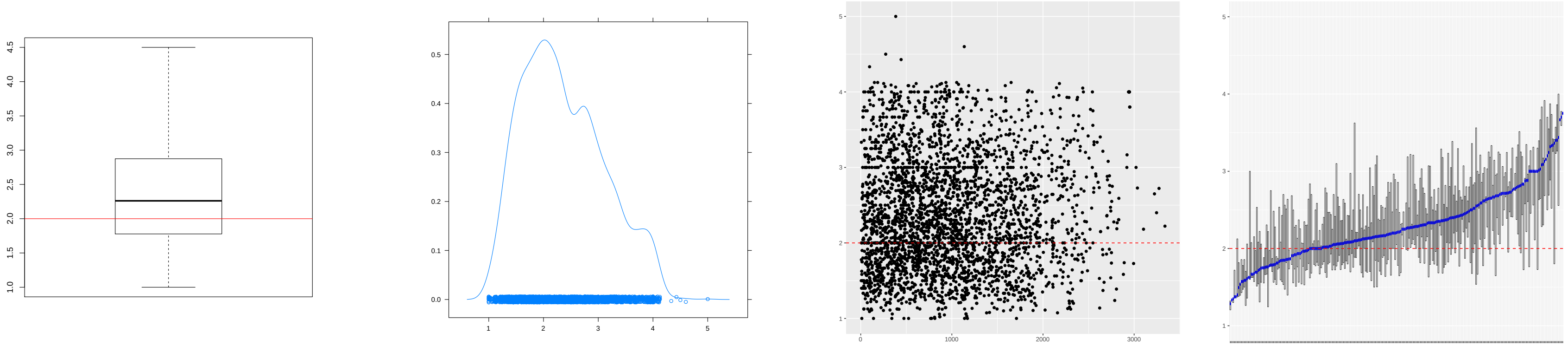

Sample	Y03
Mean	2.30
Median	2.31
% <2	40.9

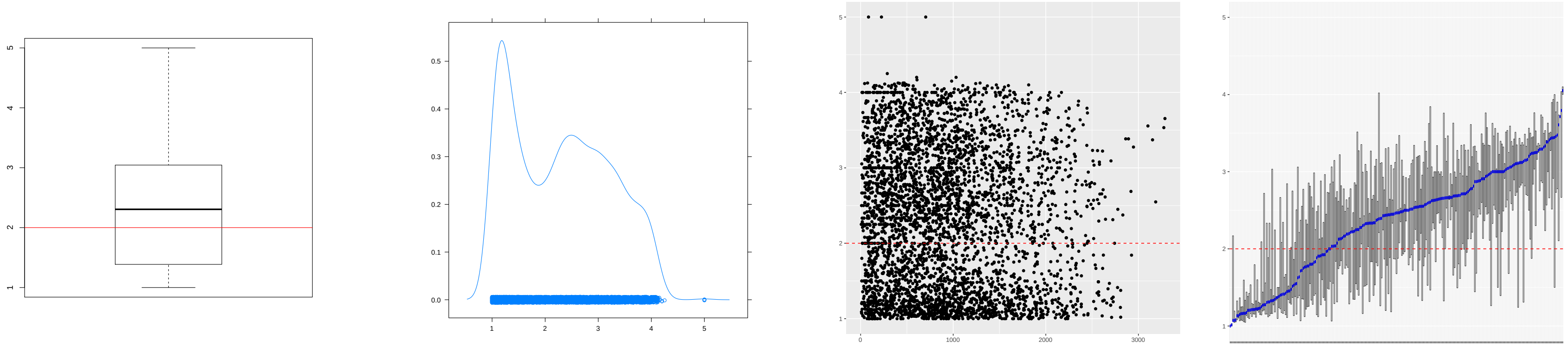

Sample	Y06
Mean	2.67
Median	2.50
% <2	16.5

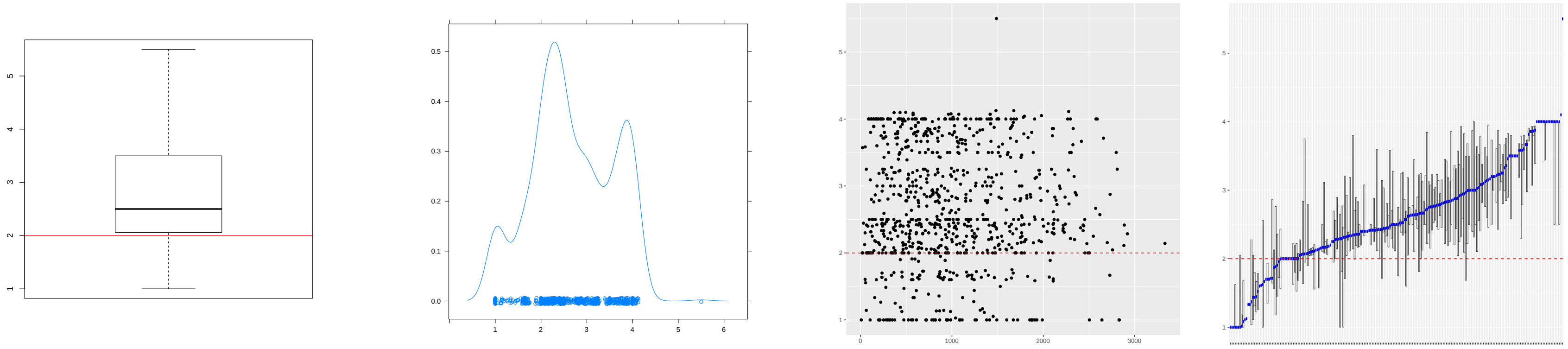

Sample	Y04
Mean	2.61
Median	2.66
% <2	19.8

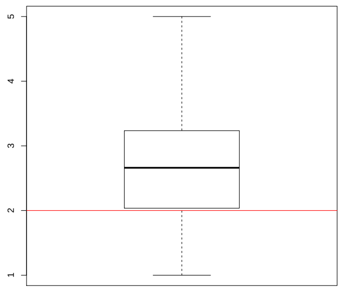
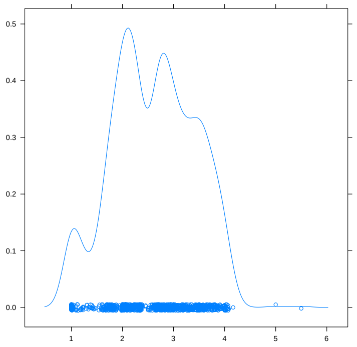
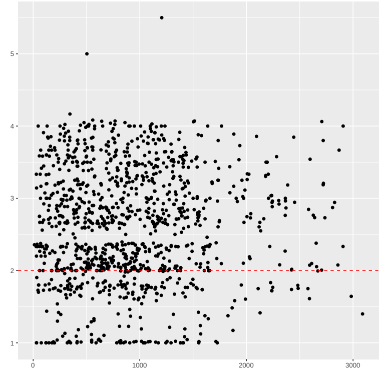
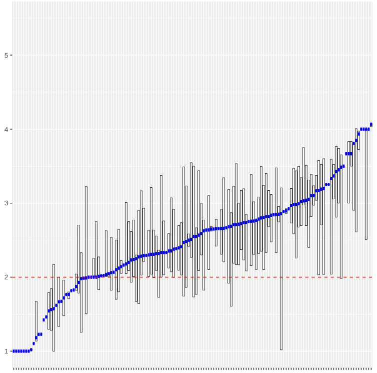

Sample	R89
Mean	3.04
Median	3.07
% <2	6.00

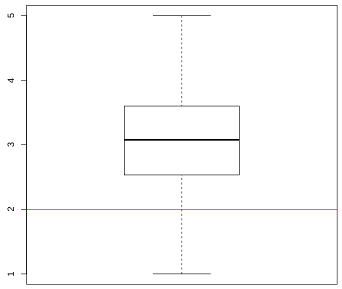
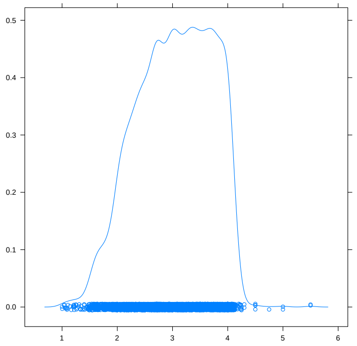
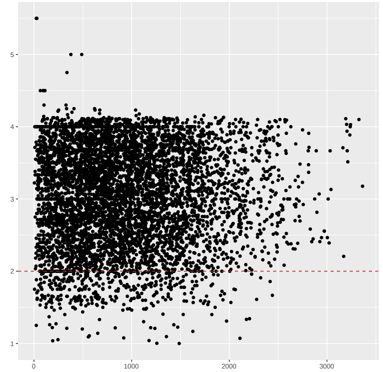
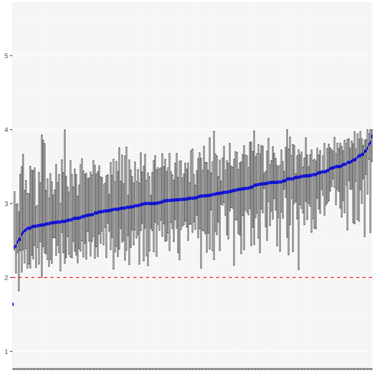

Supplement: Supplementary file 4 [file Table_4.pdf]
