# Supplementary material for: A Target Capture-Based Method to Estimate Ploidy From Herbarium Specimens
Source: Front Plant Sci. 2019 Jul 24;10:937. doi: 10.3389/fpls.2019.00937 (PMC6667659; doi:10.3389/fpls.2019.00937)
Supplement: Supplementary file 5 [file Table_5.pdf]

Supplementary Table 5. Allelic ratio (AR), allelic frequencies (AF) of the less frequent allele and allele ratio values expected for ploidy levels between 2x and 16x. In red, are those allelic ratios and frequencies that would be discarded during the analysis step that removes background noise step (see Materials and Methods).

Ploidy	AR	AF	AR	AF	AR	AF	AR	AF	AR	AF	AR	AF	AR	AF	AR	AF	Ploidy	Expected allele ratios						
2x	1:1	0.5															**2x**	**1**						
3x	2:1	0.3															3x				2			
4x	3:1	0.3	2:2	0.5													**4x**	**1**				3		
5x	4:1	0.2	3:2	0.4													5x	1.5					4	
6x	5:1	0.2	4:2	0.3	3:3	0.5											**6x**	**1**			**2**			**5**
7x	6:1	0.1	5:2	0.3	4:3	0.4											7x	1.3			2.5			
8x	7:1	0.1	6:2	0.3	5:3	0.4	4:4	0.5									**8x**	**1**	**1.7**			**3**		
9x	8:1	0.1	7:2	0.2	6:3	0.3	5:4	0.4									9x	1.3			2	3.5		
10x	9:1	0.1	8:2	0.2	7:3	0.3	6:4	0.4	5:5	0.5							**10x**	**1**	**1.5**		**2.3**		**4**	
11x	10:1	0.1	9:2	0.2	8:3	0.3	7:4	0.4	6:5	0.5							11x	1.2	1.8		2.7		4.5	
12x	11:1	0.1	10:2	0.2	9:3	0.3	8:4	0.3	7:5	0.4	6:6	0.5					**12x**	**1**	**1.4**		**2**	**3**		**5**
13x	12:1	0.1	11:2	0.2	10:3	0.2	9:4	0.3	8:5	0.4	7:6	0.5					13x	1.2	1.6		2.3	3.3		5.5
14x	13:1	0.1	12:2	0.1	11:3	0.2	10:4	0.3	9:5	0.4	8:6	0.4	7:7	0.5			**14x**	**1**	**1.3**	**1.8**	**2.5**	**3.7**		
15x	14:1	0.1	13:2	0.1	12:3	0.2	11:4	0.3	10:5	0.3	9:6	0.4	8:7	0.5			15x	1.1	1.5		2	2.8	4	
16x	15:1	0.1	14:2	0.1	13:3	0.2	12:4	0.3	11:5	0.3	10:6	0.4	9:7	0.4	8:8	0.5	**16x**	**1**	**1.3**	**1.7**	**2.2**	**3**	**4.3**	
