# Supplementary material for: A Target Capture-Based Method to Estimate Ploidy From Herbarium Specimens
Source: Front Plant Sci. 2019 Jul 24;10:937. doi: 10.3389/fpls.2019.00937 (PMC6667659; doi:10.3389/fpls.2019.00937)

Data Sheet 1. Expected allelic ratio curves from the distribution of 1,000 normalized proportions for each ploidy level ranging from 2x to 16x. In grey, those curves that are expected to be discarded during the removal of sequencing noise (see Materials and Methods).

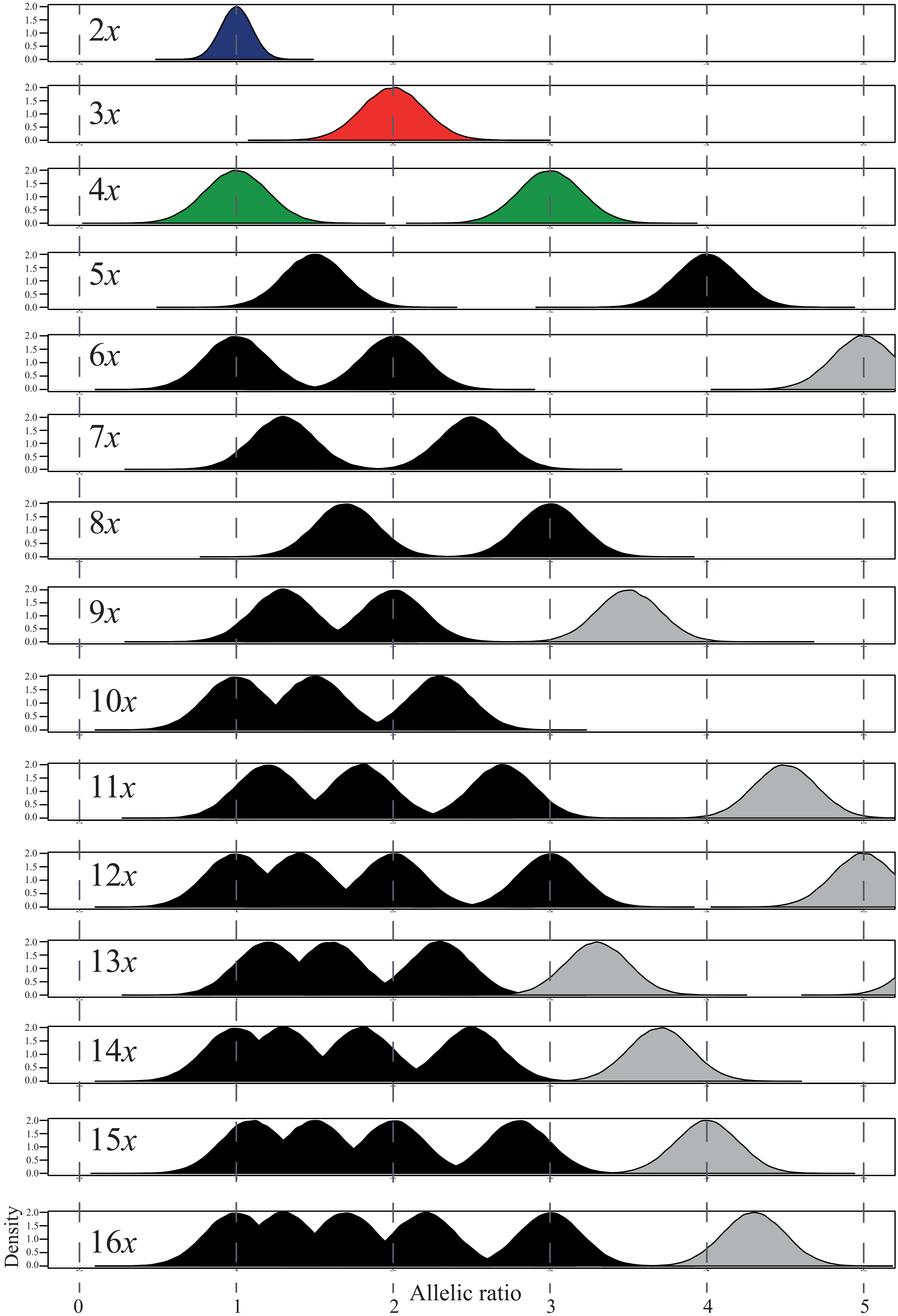

Supplement: Supplementary file 6 [file Data_Sheet_1.pdf]
